# Cardiovascular magnetic resonance normal values in children for biventricular wall thickness and mass

**DOI:** 10.1186/s12968-020-00692-2

**Published:** 2021-01-04

**Authors:** Sylvia Krupickova, Julian Risch, Sabiha Gati, Amke Caliebe, Samir Sarikouch, Philipp Beerbaum, Filippo Puricelli, Piers E. F. Daubeney, Courtney Barth, Rick Wage, Simona Boroni Grazioli, Anselm Uebing, Dudley J. Pennell, Inga Voges

**Affiliations:** 1 CMR Unit, Royal Brompton Hospital, London, UK; 2 National Heart and Lung Institute, Imperial College, London, UK; 3 Department of Congenital Heart Disease and Pediatric Cardiology, University Hospital Schleswig-Holstein, Campus Kiel, Arnold-Heller-Str. 3, 24105 Kiel, Germany; 4 Department of Medical Informatics and Statistics, University Hospital Schleswig-Holstein, Campus Kiel, Kiel, Germany; 5 Medical Faculty, Kiel University, Kiel, Germany; 6 Department of Heart, Thoracic, Transplantation and Vascular Surgery, Hannover Medical School, Hannover, Germany; 7 Department of Pediatric Cardiology and Pediatric Intensive Care Medicine At the Hannover Medical School, Hannover, Germany

**Keywords:** Myocardial thickness, Normal values, Children, Cardiovascular magnetic resonance

## Abstract

**Background:**

Pediatric patients are becoming increasingly referred for cardiovascular magnetic resonance (CMR). Measurement of ventricular wall thickness is typically part of the assessment and can be of diagnostic importance, e.g. in arterial hypertension. However, normal values for left ventricular (LV) and right ventricular (RV) wall thickness in pediatric patients are lacking. The aim of this study was to establish pediatric centile charts for segmental LV and RV myocardial thickness in a retrospective multicenter CMR study.

**Methods:**

CMR was performed in 161 healthy children and adolescents with an age range between 6 and 18 years from two centers in the UK and Germany as well as from a previously published CMR project of the German Competence Network for Congenital Heart Defects. LV myocardial thickness of 16 segments was measured on the short axis stack using the American Heart Association segmentation model. In addition, the thickness of the RV inferior and anterior free wall as well as biventricular mass was measured.

**Results:**

The mean age (standard deviation) of the subjects was 13.6 (2.9) years, 64 (39.7%) were female. Myocardial thickness of the basal septum (basal antero- and inferoseptal wall) was 5.2 (1.1) mm, and the basal lateral wall (basal antero- and inferolateral) measured 5.1 (1.2) mm. Mid-ventricular septum (antero- and inferoseptal wall) measured 5.5 (1.2) mm, and mid-ventricular lateral wall (antero- and inferolateral wall) was 4.7 (1.2) mm. Separate centile charts for boys and girls for all myocardial segments and myocardial mass were created because gender was significantly correlated with LV myocardial thickness (p < 0.001 at basal level, p = 0.001 at midventricular level and p = 0.005 at the apex) and biventricular mass (LV, p < 0.001; RV, p < 0.001).

**Conclusion:**

We established CMR normal values of segmental myocardial thickness and biventricular mass in children and adolescents. Our data are of use for the detection of abnormal myocardial properties and can serve as a reference in future studies and clinical practice.

## Introduction

Cardiovascular magnetic resonance (CMR) is a well-established imaging modality for assessment of cardiac disease in the adult and pediatric population [1]. It is complementary to other modalities such as echocardiography, computed tomography and cardiac catheterization and provides anatomical as well as detailed functional data.

CMR is considered the reference standard for non-invasive biventricular volumetric measurements [2]. The prognostic value of the volumetric analysis is well known and has been shown repeatedly across various cardiac diseases [3–6].

A recent review about normal CMR values in pediatric patients revealed that there are limited data available for children and adolescents with regard to measured parameters, sample sizes and age range [7]. Moreover, to our knowledge none of the studies reporting normal values in pediatric patients focused on myocardial thickness [8–13].

Children with cardiomyopathy or a family history of cardiomyopathy are increasingly being referred for CMR assessment [14]. Hypertrophic cardiomyopathy can exhibit concentric hypertrophy of the whole ventricle or eccentric thickening of only some segments. It is crucial to interpret the measurements correctly as early diagnosis determines further follow up and prognosis [15]. The same also applies for other cardiomyopathies. In addition, assessment of myocardial thickness in congenital heart diseases (e.g. left ventricular (LV) and right ventricular (RV) obstructive diseases, single ventricle physiology) or acquired diseases (e.g. systemic and pulmonary hypertension) can be of importance to assess severity of these conditions [16].

Unlike in the adult population, where the normal cut-off values for myocardial thickness are well defined [17], this does not defined for pediatric patients. Myocardial thickness of both ventricles in children is currently assessed subjectively as age related segmental thickness data throughout all ages is lacking. LV myocardial thickness increases with age and with respect to regional changes it decreases from the base to the apex [18]. Thickness of different RV regions also varies. In addition, gender differences have been found [17].

The aim of this multicentre retrospective study was: (1) to establish centile charts for myocardial thickness of 16 segments of the LV and 6 segments of the RV analyzing previously acquired scans of healthy children, and (2) to assess the impact of demographic parameters including gender on wall thickness measurements.

## Methods

### Study population

Scans of healthy children for this multicenter retrospective study were recruited from Royal Brompton Hospital in London, UK (n = 117), the University Hospital Schleswig–Holstein, Campus in Kiel, Germany (n = 16) and from a previously published CMR project of the German Competence Network for Congenital Heart Defects (n = 28).

The indications for the scans were as follows: (1) non-diagnostic echocardiographic scan, (2) uncertainty about the anatomical structures on echocardiography, (3) syncope or (4) chest pain with low pre-scan probability of being cardiac in origin, (5) participation in a previous study. Exclusion criteria comprised: (1) congenital or acquired heart disease, (2) arterial hypertension (3) medication for arterial hypertension, (3) other types of disease that involve structural and functional abnormalities of the heart and (4) pregnancy.

The study was approved by the Local Research Ethics Committee and by Health Research Authority (HRA, reference number 19/HRA/2041). Parents or guardians signed a written consent.

### Cardiovascular magnetic resonance

All CMR scans were performed at 1.5 T. Contiguous standard short axis cines with full myocardial coverage were acquired using ECG-gated balanced steady-state free-precession (bSSFP) sequences. All images were analyzed with validated software (cvi42, Circle Cardiovascular Imaging, Calgary, Canada). Volumes and mass were calculated excluding the papillary muscles and all measurements were indexed to body surface area (BSA, calculated using DuBois formula). The following parameters were calculated: BSA indexed LV and RV end-diastolic volume (LVEDV/BSA, RVEDV/BSA), indexed LV and RV end-systolic volume (LVESV/BSA, RVESV/BSA), LV and RV ejection fraction (LVEF, RVEF), indexed LV and RV stroke volume (LVSV/BSA, RVSV/BSA), indexed LV and RV mass (LV mass, RV mass) and cardiac index.

**Fig. 1:**
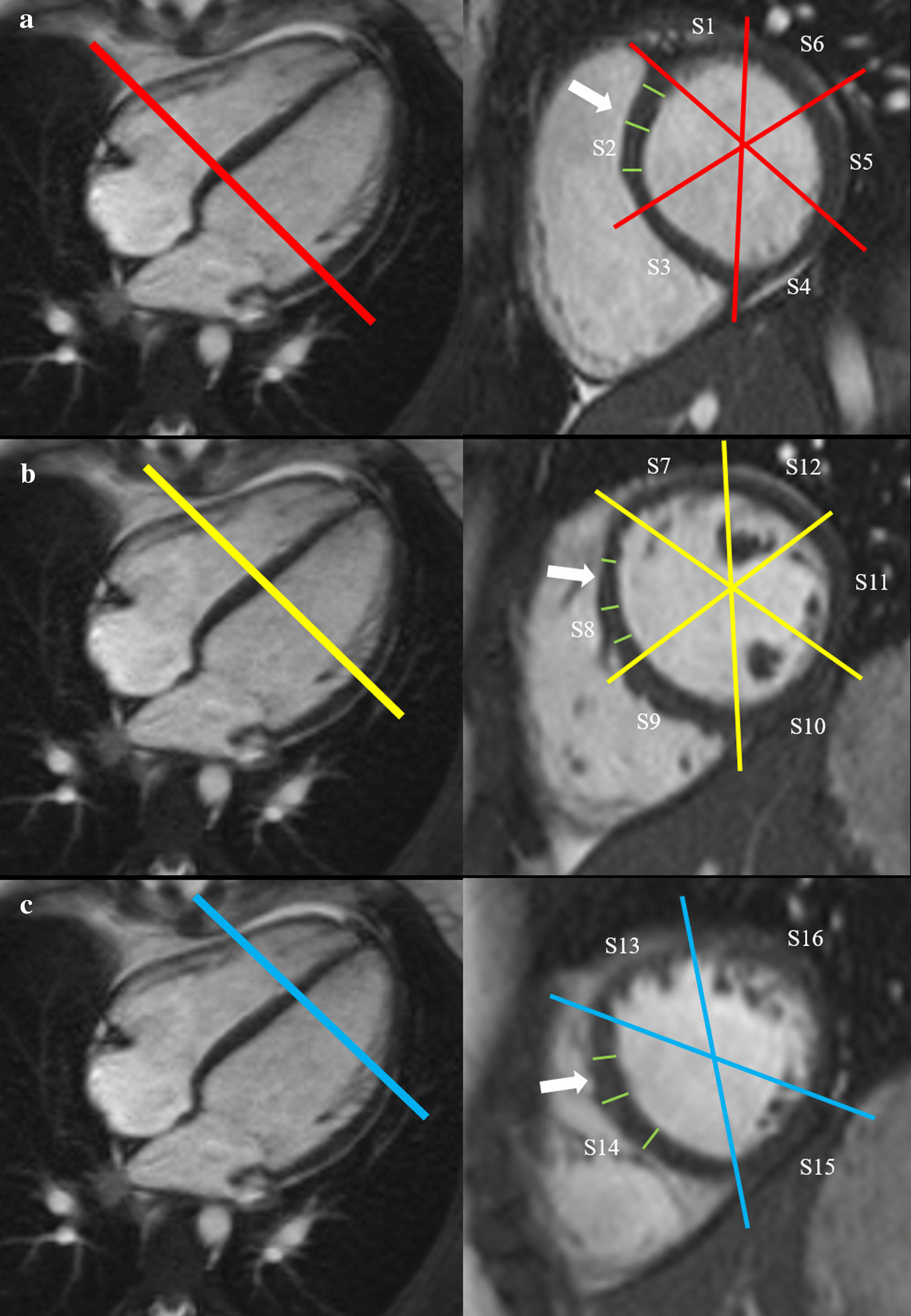
Measurement of left ventricular (LV) myocardial thickness on short axis cine images at the end of diastole. First, the basal (**a**), mid-ventricular (**b**) and apical (**c**) level was defined. Second, all 16 segments (S1-S16) of the American Heart Association segmentation model were determined. And third, myocardial thickness was measured three times for each segment shown for segments S2, S8 and S14 (white arrows). S, segment

LV myocardial wall thickness of 16 segments was measured in the short axis stack at the end of diastole using the American Heart Association segmentation model (Fig. 1).

**Fig. 2:**
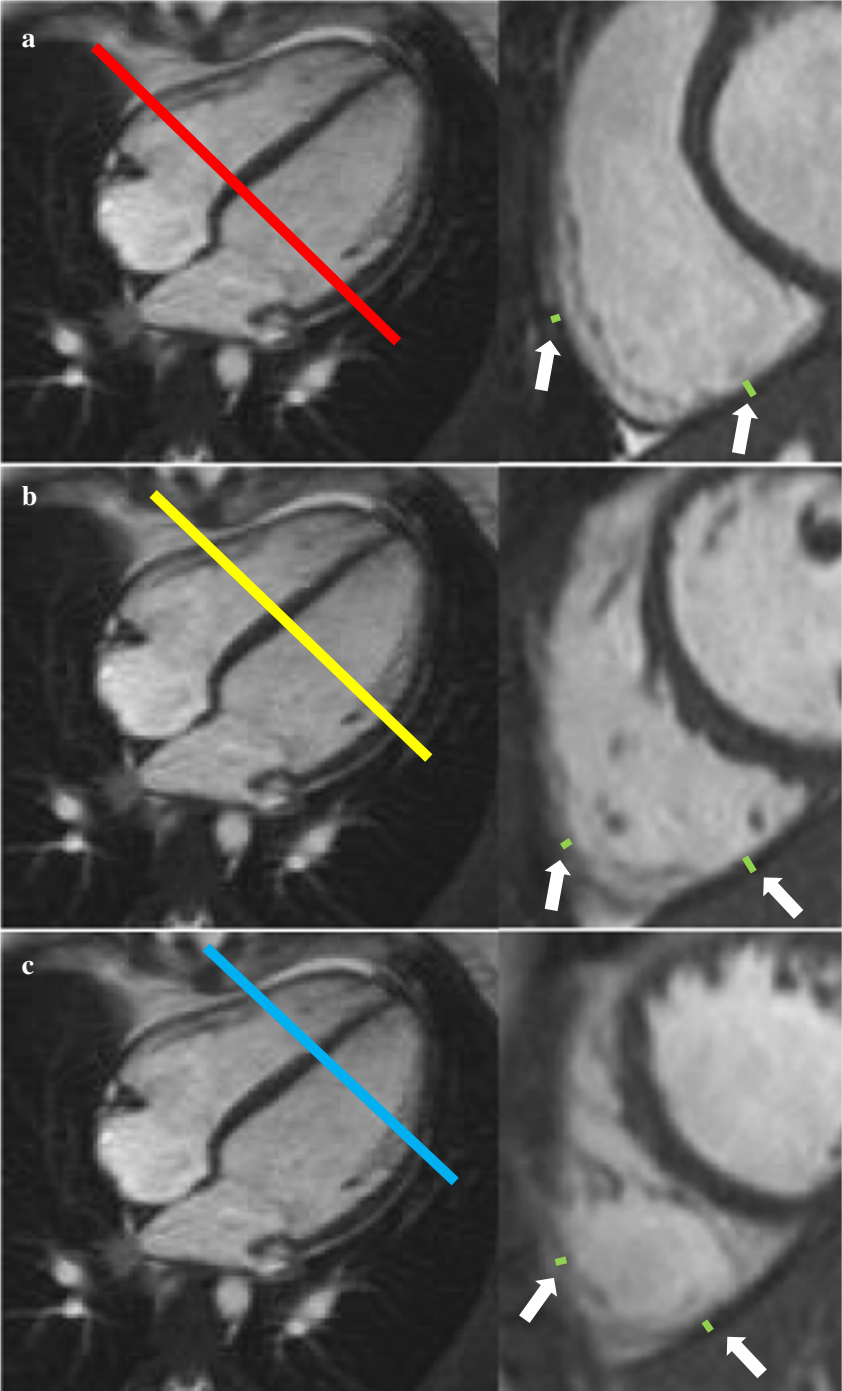
Measurement of right ventricular myocardial thickness of the basal (**a**), mid-ventricular (**b**) and apical (**c**) inferior and anterior free wall (white arrows) from short axis cine images at end of diastole

In addition, the thickness of the RV inferior and anterior free walls was measured at the basal, mid-ventricular and apical level in the short axis stack also at the end-diastole (Fig. 2). Papillary muscles, trabeculations and trabecula septomarginalis were excluded from all myocardial thickness measurements.

### Statistical analysis

The software R (version 3.6.2, R Foundation for Statistical Computing, Vienna, Austria) was used for the statistical analysis [19]. All tests were performed two-sided with a significance level of 0.05. Data were normally distributed and therefore parametric tests were used.

The statistical analysis was performed stratified for girls and boys and separately for the LV and the RV. The mean of three wall thickness measurements for each segment (Fig. 1) was used for the analysis for each segment of the 16 LV segments. For the RV, the mean of two measurements was used for the six segments. Measurements of segments S1 to S6 of the LV were combined by calculating the mean of six values to enable an evaluation of the basal component. The same was done for the mid-ventricular component with segments S7 to S12 and for the apical component with segments S13 to S16. For the RV, the corresponding two segments were combined to generate basal, mid-ventricular and apical component.

Centile graphs and tables were generated according to the LMS-method of Cole and Green [20]. An extended version of this method is implemented in the R package gamlss which was used for the analysis [21].

The impact of demographic factors on wall thickness and myocardial mass was analyzed for each variable separately and in a multiple fashion using linear regression models with and without interactions. Outcome variables were basal, mid-ventricular and apical wall thickness as well as diastolic myocardial mass for the LV and RV, while demographic variables were gender, BSA, age, body height, body weight and average heart rate. Because gender showed an interaction with several of these variables, multiple analyses were stratified for gender. Model selection was performed by backward selection and a p value threshold of 0.05.

Two experienced operators with 14 years (IV) and 3 years (SBG) of experience evaluated the measurements separately for all segments of the LV in 30 children. The inter-observer agreement was evaluated by the intraclass correlation coefficient (ICC). For this, the R package irr with the command icc (parameters: model = twoway, type = agreement, unit = single) was applied [22].

## Results

**Fig. 3:**
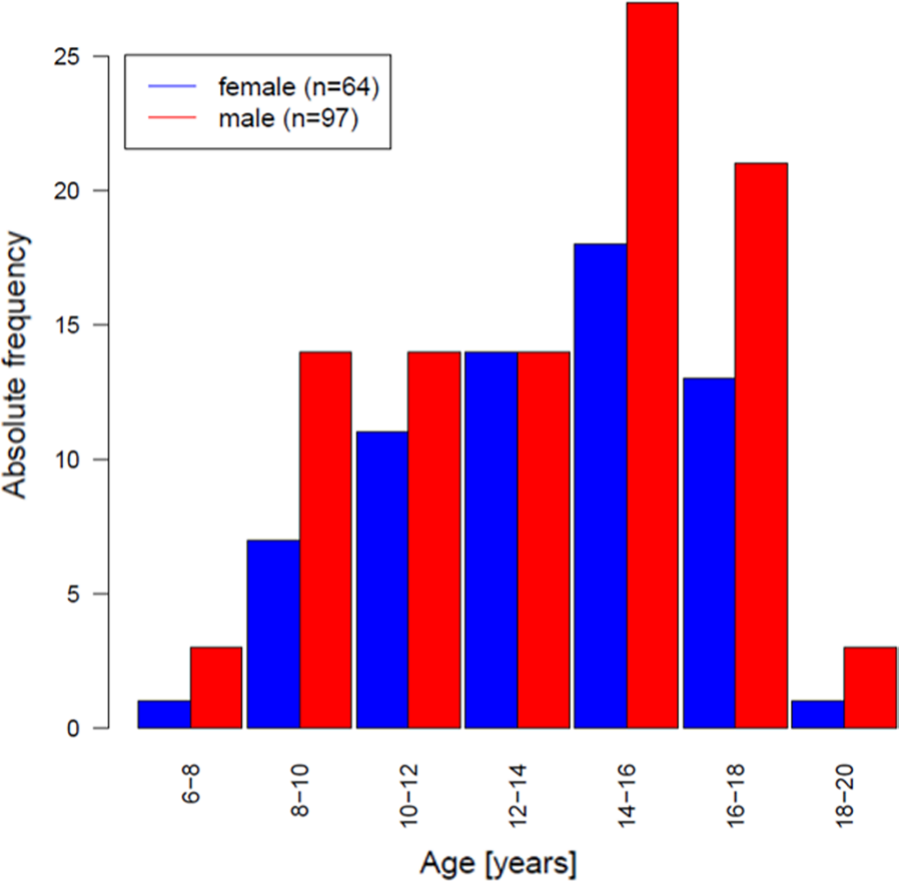
Histogram showing the distribution of included study participants according to their age

CMR scans of 161 healthy subjects of 6–18 years of age fulfilled the criteria and were included into the study (Fig. 3). The demographic data are displayed in Table 1. All scans were performed without any form of sedation. The study was approved by the ethics committee of the Medical faculty of the Christian-Albrechts University.

**Table 1:** Demographic data. Continuous variables are shown as mean (SD), categorical variables as absolute numbers (percentages)

	All study subjects	Girls	Boys
N	161	64 (39.8%)	97 (60.2%)
Age, years	13.6 (2.9)	13.8 (2.9)	13.5 (3.0)
Weight, kg	53 (16.8)	51.9 (15.6)	53.8 (17.6)
Height, cm	160.9 (17.1)	159.1 (14.4)	161.0 (18.7)
Body surface area, m^2^	1.5 (0.3)	1.5 (0.3)	1.6 (0.3)
Heart rate, bpm	79.5 (16.3)	81.8 (15.1)	78.0 (17.0)
LVEDV/BSA, ml	79.3 (14.2)	74.2 (10.1)	82.7 (14.0)
LVESV/BSA, ml	32.2 (7.4)	29.5 (5.2)	32.3 (8.4)
LVSV/BSA, ml	48.2 (7.8)	44.8 (7.2)	50.4 (7.4)
LVEF, %	60.9 (5.0)	60.3 (4.9)	61.3 (5.1)
LV mass, g/m^2^	50.0 (10.4)	44.9 (8.3)	53.3 (10.3)
Cardiac index, l/min/m^2^	3.8 (0.8)	3.6 (0.8)	3.9 (0.9)
RVEDV/BSA, ml	88.0 (15.6)	81.2 (12.3)	92.5 (16.0)
RVESV/BSA, ml	39.4 (9.1)	36.3 (7.3)	41.4 (9.6)
RVSV/BSA, ml	48.5 (9.0)	44.9 (8.1)	50.9 (8.8)
RVEF, %	55.4 (5.3)	55.3 (6.0)	55.4 (4.8)
RV mass, g/m^2^	21.7 (4.1)	20.0 (3.5)	22.8 (4.1)

*N* total number of study objects* BSA* body surface area, *EDV* end-diastolic volume, *EF* ejection fraction, *ESV* end-systolic volume, *LV* left ventricle, *RV* right ventricle

### Myocardial thickness of the LV segments and LV mass

Myocardial thickness of the basal septum (basal antero- and inferoseptal wall) of the whole study group was 5.2 ± 1.1 mm (mean ± SD), and the basal lateral wall (basal antero- and inferolateral) measured 5.1 ± 1.2 mm. Mid-ventricular septum (antero- and inferoseptal wall) measured 5.5 ± 1.2 mm, and mid-ventricular lateral wall (antero- and inferolateral wall) was 4.7 ± 1.2 mm. LV mass at end-diastole ranged between 23.9 to 156.0 g in the entire study group.

Strong correlations were observed between BSA and body weight (r = 0.96, female: r = 0.95, male: r = 0.97, r Pearson correlation coefficient) and between BSA and body height (r = 0.90, female: r = 0.84, male: r = 0.92).

Linear regression (Table 2) showed that, if considered separately, gender, BSA, height, weight and age were strong predictors of the LV basal, mid-ventricular and apical myocardial thickness as well as LV mass. For myocardial mass also average heart rate was a strong predictor. Table 3 shows the multivariable model. For basal wall thickness, BSA was the strongest determinant, while height showed only small additional impact in girls. BSA was also strong determinant for mid-ventricular wall thickness in multivariable analysis. Age showed a small impact in girls. For apical wall thickness, only body weight was a strong predictor in girls and only body height in boys. BSA and average heart rate showed an influence on diastolic myocardial mass in both girls and boys.

**Table 2:** Linear regression analysis showing impact of demographic parameters on LV myocardial thickness and mass separately for each variable

Variable	Regression coefficient	Standard error	P value
Myocardial thickness, LV base			
Age	0.20	0.021	< 2 × 10^–16^
Men	0.55	0.15	0.00036
Body surface area	2.50	0.15	< 2 × 10^–16^
Body height	0.040	0.0032	< 2 × 10^–16^
Body weight	0.044	0.0029	< 2 × 10^–16^
Average heart rate	− 0.011	0.0046	0.018
Myocardial thickness, LV mid-cavity			
Age	0.17	0.024	1.2 × 10^–11^
Men	0.52	0.16	0.0012
Body surface area	2.35	0.19	< 2 × 10^–16^
Body height	0.037	0.0037	< 2 × 10^–16^
Body weight	0.041	0.0035	< 2 × 10^–16^
Average heart rate	− 0.011	0.0049	0.025
Myocardial thickness, LV apex			
Age	0.13	0.019	6.9 × 10^–11^
Men	0.35	0.12	0.0049
Body surface area	1.66	0.16	< 2 × 10^–16^
Body height	0.027	0.0030	5.7 × 10^–16^
Body weight	0.029	0.0029	< 2 × 10^–16^
Average heart rate	− 0.0078	0.0038	0.040
Myocardial mass			
Age	6.58	0.54	< 2 × 10^–16^
Men	16.26	4.33	0.00024
Body surface area	78.73	3.56	< 2 × 10^–16^
Body height	1.33	0.08	< 2 × 10^–16^
Body weight	1.36	0.076	< 2 × 10^–16^
Average heart rate	− 0.63	0.13	1.7 × 10^–6^

**Table 3:** Multiple regression analysis showing impact of demographic parameters on LV myocardial thickness and mass

Variable	Girls			Boys		
	Regression coefficient	Standard error	P value	Regression coefficient	Standard error	P value
Myocardial thickness at basal left ventricle						
Body surface area	3.37	0.42	4.4 × 10^–11^	2.47	0.18	< 2 × 10^–16^
Body height	− 0.021	0.0077	0.0081	–	–	–
Myocardial thickness at mid-ventricular left ventricle						
Body surface area	3.38	0.45	3.9 × 10^–10^	2.26	0.21	< 2 × 10^–16^
Age	− 0.13	0.041	0.0037	–	–	–
Myocardial thickness at apical left ventricle						
Body weight	0.029	0.0039	2.0 × 10^–10^	–	–	–
Body height	–	–	–	0.028	0.0035	3.0 × 10^–12^
Myocardial mass						
Body surface area	78.21	7.61	7.5 × 10^–15^	63.59	7.56	4.6 × 10^–13^
Age	− 1.65	0.71	0.024	1.95	0.86	0.026
Average heart rate	− 0.27	0.094	0.0057	− 0.16	0.078	0.049

Only significant results are displayed

**Fig. 4:**
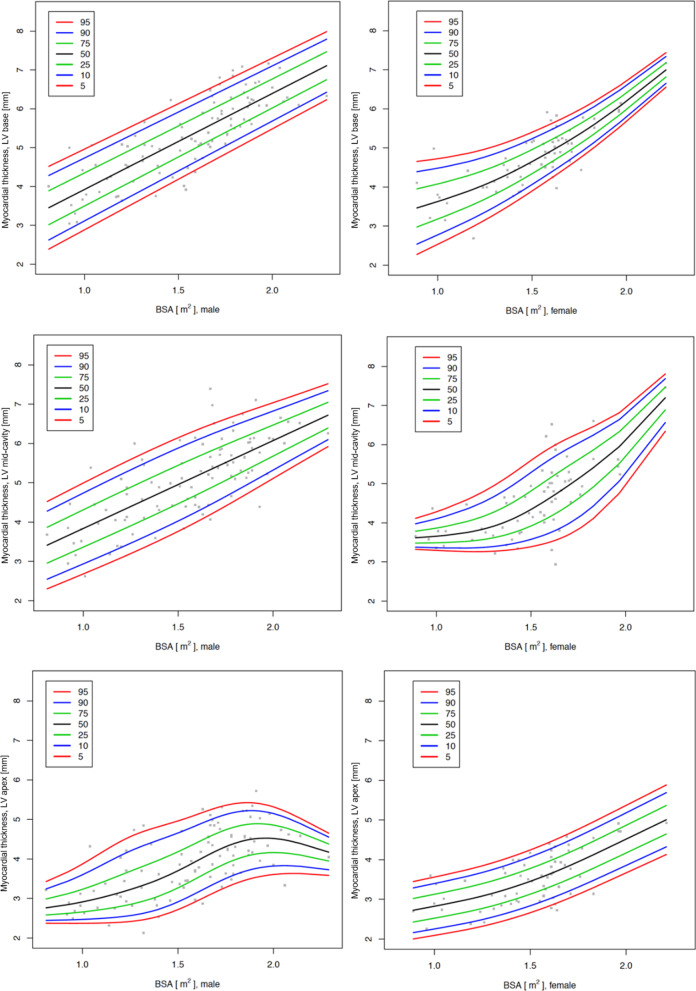
Centile charts showing myocardial thickness at LV base, mid-cavity and apex by body surface area (BSA) and gender. Colors correspond to the given centiles

**Fig. 5:**
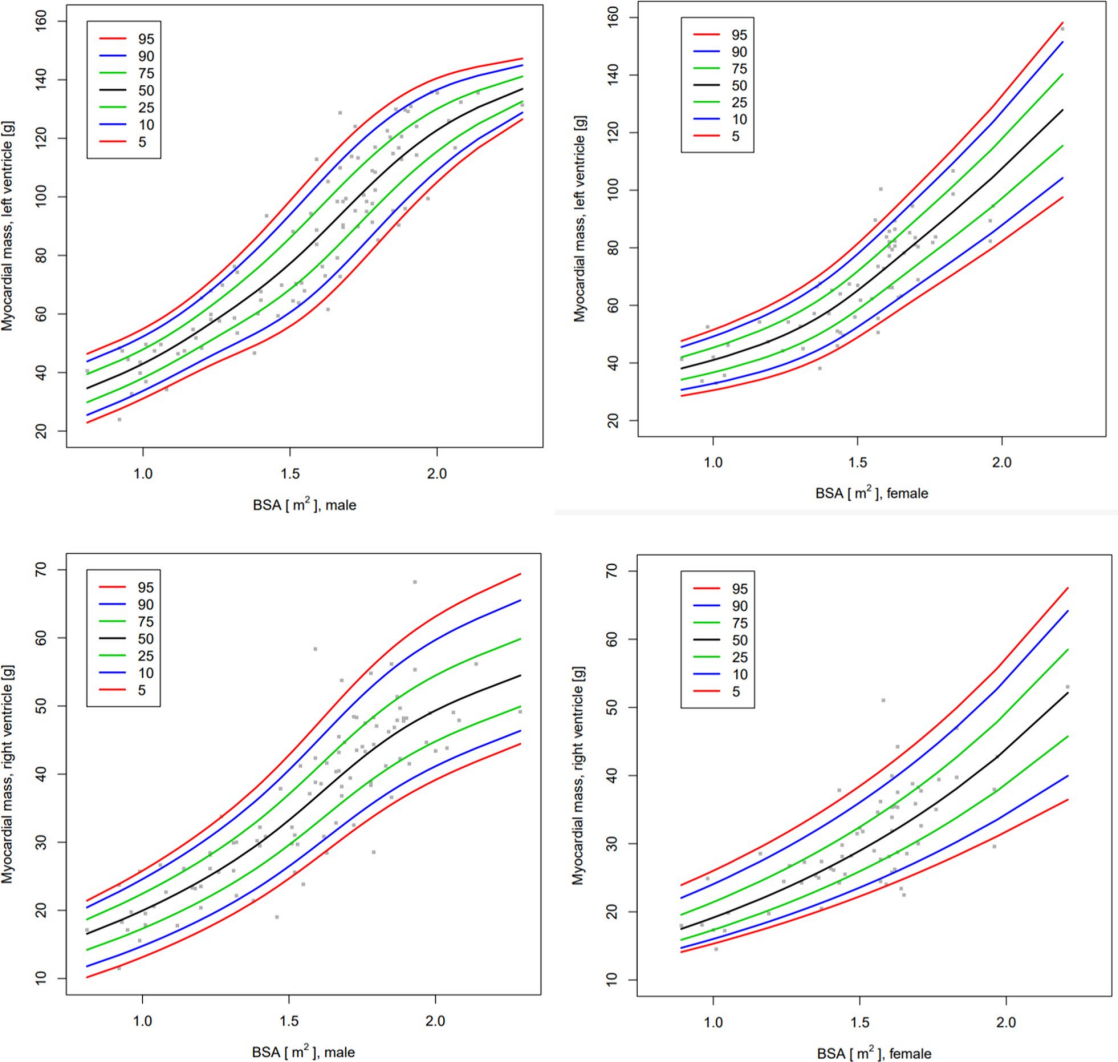
Centile charts showing LV and RV myocardial mass by body surface area (BSA) and gender. Colors correspond to the given centiles

**Fig. 6:**
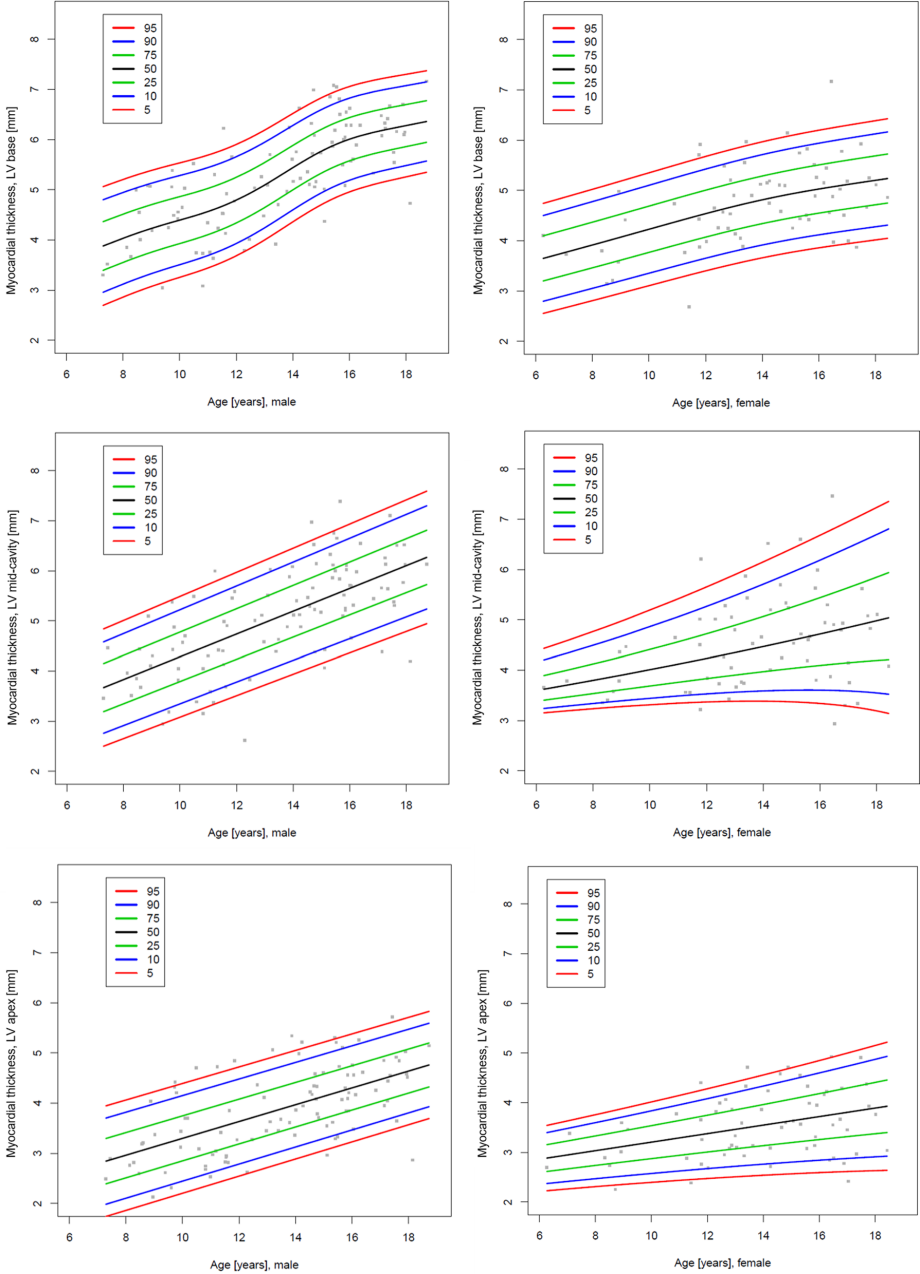
Centile charts showing myocardial thickness at LV base, mid-cavity and apex by age and gender. Colors correspond to the given centiles

**Fig. 7:**
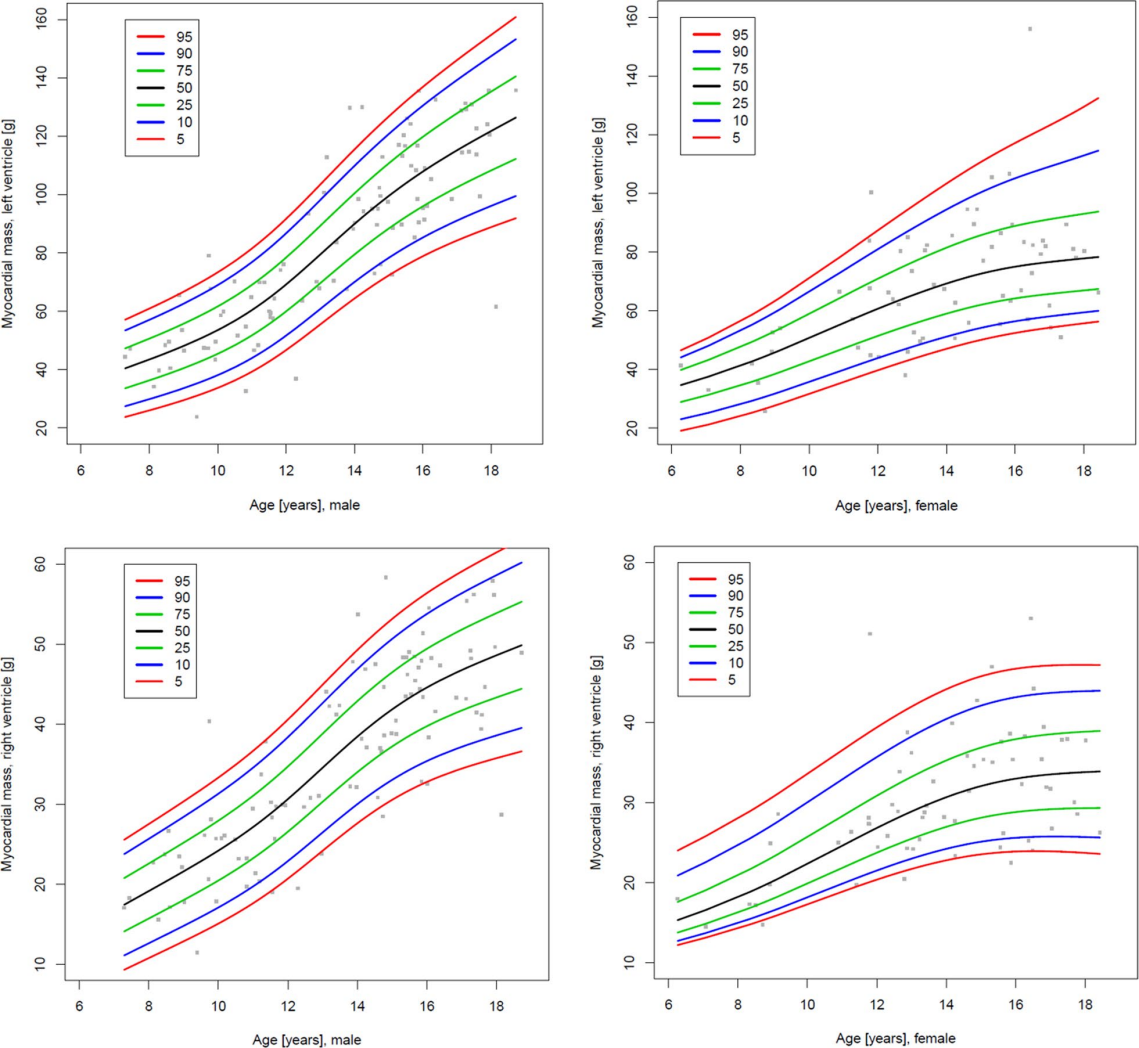
Centile charts showing LV and RV myocardial mass by age and gender. Colors correspond to the given centiles

As a consequence of the regression analysis above, the centile charts and tables in the main manuscript are shown by BSA. Figure 4 shows the centile charts for the myocardial thickness of the LV at the basal, mid and apical level and Fig. 5 shows the centile charts for LV mass. Tables 4, 5, 6 and 7 show the centiles of myocardial thickness at each chamber level and myocardial mass by BSA and gender. Figures and Tables showing myocardial thickness centiles for each individual segment of the 16-segment model by BSA and gender and also age and gender are given in Additional file 1. Centile charts for LV myocardial thickness and mass by age and gender are also displayed for completeness (Figs. 6 and 7).

**Table 4:** Centiles of the myocardial thickness in mm of the LV at the basal level by body surface area (BSA) and gender

BSA (m^2^)	5th centile	10th centile	25th centile	50th centile	75th centile	90th centile	95th centile
Boys							
0.8	2.4	2.6	3.0	3.4	3.9	4.3	4.5
0.9	2.6	2.9	3.2	3.7	4.1	4.5	4.7
1.0	2.9	3.1	3.5	3.9	4.4	4.7	5.0
1.1	3.1	3.4	3.8	4.2	4.6	5.0	5.2
1.2	3.4	3.6	4.0	4.4	4.8	5.2	5.4
1.3	3.7	3.9	4.3	4.7	5.1	5.4	5.7
1.4	3.9	4.1	4.5	4.9	5.3	5.7	5.9
1.5	4.2	4.4	4.8	5.2	5.6	5.9	6.1
1.6	4.4	4.7	5.0	5.4	5.8	6.2	6.4
1.7	4.7	4.9	5.3	5.7	6.0	6.4	6.6
1.8	5.0	5.2	5.5	5.9	6.3	6.6	6.8
1.9	5.2	5.4	5.8	6.2	6.5	6.9	7.1
2.0	5.5	5.7	6.0	6.4	6.8	7.1	7.3
2.1	5.7	5.9	6.3	6.6	7.0	7.3	7.5
2.2	6.0	6.2	6.5	6.9	7.3	7.6	7.8
Girls							
0.8	2.1	2.3	2.8	3.3	3.9	4.3	4.6
0.9	2.3	2.6	3.0	3.5	4.0	4.4	4.7
1.0	2.5	2.8	3.2	3.6	4.1	4.5	4.7
1.1	2.8	3.0	3.4	3.8	4.2	4.6	4.8
1.2	3.0	3.2	3.6	4.0	4.4	4.7	4.9
1.3	3.3	3.5	3.8	4.2	4.5	4.8	5.0
1.4	3.6	3.8	4.1	4.4	4.7	5.0	5.2
1.5	3.9	4.1	4.3	4.7	5.0	5.2	5.4
1.6	4.2	4.4	4.6	4.9	5.2	5.5	5.6
1.7	4.6	4.7	4.9	5.2	5.5	5.7	5.9
1.8	4.9	5.0	5.3	5.5	5.8	6.0	6.1
1.9	5.3	5.4	5.6	5.8	6.1	6.3	6.4
2.0	5.7	5.8	6.0	6.2	6.4	6.6	6.7
2.1	6.1	6.2	6.4	6.6	6.8	6.9	7.1
2.2	6.5	6.6	6.8	7.0	7.1	7.3	7.4

**Table 5:** Centiles of the myocardial thickness in mm of the LV at the mid-ventricular level by BSA and gender

BSA (m^2^)	5th centile	10th centile	25th centile	50th centile	75th centile	90th centile	95th centile
Boys							
0.8	2.3	2.5	2.9	3.4	3.8	4.3	4.5
0.9	2.5	2.7	3.2	3.6	4.1	4.5	4.7
1.0	2.7	2.9	3.4	3.8	4.3	4.7	5.0
1.1	2.9	3.1	3.6	4.1	4.5	5.0	5.2
1.2	3.1	3.4	3.8	4.3	4.8	5.2	5.5
1.3	3.3	3.6	4.0	4.5	5.0	5.4	5.7
1.4	3.5	3.8	4.2	4.7	5.2	5.7	5.9
1.5	3.8	4.0	4.5	5.0	5.4	5.9	6.1
1.6	4.0	4.3	4.7	5.2	5.7	6.1	6.3
1.7	4.3	4.5	4.9	5.4	5.9	6.3	6.5
1.8	4.5	4.8	5.2	5.6	6.1	6.5	6.7
1.9	4.8	5.1	5.4	5.9	6.3	6.7	6.9
2.0	5.1	5.3	5.7	6.1	6.5	6.8	7.0
2.1	5.4	5.6	5.9	6.3	6.7	7.0	7.2
2.2	5.7	5.9	6.2	6.5	6.9	7.2	7.4
Girls							
0.8	3.3	3.4	3.5	3.6	3.7	3.9	4.0
0.9	3.3	3.4	3.5	3.6	3.8	4.0	4.1
1.0	3.3	3.4	3.5	3.7	3.9	4.1	4.3
1.1	3.3	3.4	3.5	3.7	4.0	4.3	4.5
1.2	3.3	3.4	3.5	3.8	4.1	4.4	4.7
1.3	3.3	3.4	3.6	3.9	4.3	4.7	4.9
1.4	3.3	3.5	3.7	4.1	4.5	5.0	5.3
1.5	3.4	3.6	3.9	4.3	4.8	5.3	5.6
1.6	3.5	3.7	4.2	4.6	5.1	5.6	5.9
1.7	3.7	4.0	4.5	5.0	5.5	5.9	6.2
1.8	4.0	4.3	4.8	5.3	5.8	6.2	6.4
1.9	4.4	4.7	5.2	5.7	6.1	6.4	6.6
2.0	5.0	5.3	5.7	6.1	6.5	6.8	6.9
2.1	5.6	5.8	6.2	6.6	6.9	7.2	7.3
2.2	6.3	6.5	6.8	7.1	7.4	7.6	7.8

**Table 6:** Centiles of the myocardial thickness in mm of the LV at the apical level by BSA and gender

BSA (m^2^)	5th centile	10th centile	25th centile	50th centile	75th centile	90th centile	95th centile
Boys							
0.8	2.4	2.4	2.6	2.8	3.0	3.2	3.4
0.9	2.4	2.5	2.6	2.8	3.1	3.4	3.6
1.0	2.4	2.5	2.7	2.9	3.2	3.6	3.9
1.1	2.4	2.5	2.7	3.0	3.4	3.8	4.2
1.2	2.4	2.5	2.8	3.2	3.6	4.1	4.4
1.3	2.5	2.6	2.9	3.3	3.8	4.3	4.7
1.4	2.6	2.7	3.1	3.5	4.0	4.5	4.8
1.5	2.7	2.9	3.3	3.7	4.2	4.7	5.0
1.6	2.9	3.2	3.5	4.0	4.4	4.9	5.1
1.7	3.2	3.4	3.8	4.2	4.6	5.0	5.3
1.8	3.4	3.6	4.0	4.4	4.8	5.2	5.4
1.9	3.5	3.8	4.1	4.5	4.9	5.2	5.4
2.0	3.6	3.8	4.2	4.5	4.9	5.2	5.3
2.1	3.6	3.8	4.1	4.4	4.7	5.0	5.1
2.2	3.6	3.8	4.0	4.3	4.6	4.8	4.9
Girls							
0.8	1.9	2.1	2.4	2.6	2.9	3.2	3.4
0.9	2.0	2.2	2.4	2.7	3.0	3.3	3.5
1.0	2.1	2.3	2.5	2.8	3.1	3.4	3.6
1.1	2.2	2.3	2.6	2.9	3.2	3.5	3.7
1.2	2.3	2.4	2.7	3.0	3.3	3.6	3.8
1.3	2.4	2.6	2.8	3.2	3.5	3.7	3.9
1.4	2.5	2.7	3.0	3.3	3.6	3.9	4.1
1.5	2.7	2.8	3.1	3.5	3.8	4.1	4.2
1.6	2.8	3.0	3.3	3.6	4.0	4.3	4.4
1.7	3.0	3.2	3.5	3.8	4.2	4.5	4.7
1.8	3.2	3.4	3.7	4.1	4.4	4.7	4.9
1.9	3.5	3.6	3.9	4.3	4.6	4.9	5.1
2.0	3.7	3.9	4.2	4.5	4.9	5.2	5.4
2.1	3.9	4.1	4.4	4.8	5.1	5.4	5.6
2.2	4.1	4.3	4.6	5.0	5.3	5.7	5.9

**Table 7:** Centiles of the myocardial mass of the LV by BSA and gender

BSA (m^2^)	5th centile	10th centile	25th centile	50th centile	75th centile	90th centile	95th centile
Boys							
0.8	22.5	25.1	29.4	34.2	39.1	43.4	46.0
0.9	26.6	29.2	33.5	38.3	43.2	47.5	50.1
1.0	31.1	33.7	38.1	43.0	47.9	52.3	54.9
1.1	36.0	38.8	43.4	48.5	53.6	58.2	61.0
1.2	41.0	44.0	49.2	54.8	60.4	65.6	68.6
1.3	45.6	49.2	55.1	61.6	68.1	74.0	77.5
1.4	50.2	54.3	61.2	68.9	76.6	83.5	87.6
1.5	55.8	60.5	68.4	77.2	86.0	93.9	98.7
1.6	63.5	68.6	77.2	86.7	96.2	104.8	109.9
1.7	73.3	78.5	87.2	96.8	106.5	115.1	120.3
1.8	84.2	89.2	974	106.6	115.8	124.1	129.1
1.9	95.1	99.6	107.1	115.5	123.8	131.3	135.8
2.0	105.1	109.0	115.5	122.8	130.1	136.6	140.6
2.1	113.7	117.0	122.5	128.7	134.8	140.3	143.6
2.2	120.9	123.7	128.2	133.3	138.4	143.0	145.7
Girls							
0.8	27.0	28.9	32.2	35.8	39.4	42.6	44.5
0.9	28.7	30.9	34.4	38.3	42.3	45.8	48.0
1.0	30.5	32.8	36.7	41.0	45.3	49.2	51.5
1.1	32.6	35.2	39.4	44.1	48.8	53.0	55.5
1.2	35.2	38.0	42.6	47.7	52.9	57.5	60.2
1.3	38.6	41.6	46.7	52.3	57.9	62.9	65.9
1.4	43.1	46.4	51.9	58.0	64.1	69.6	72.9
1.5	48.9	52.5	58.5	65.2	71.9	77.9	81.5
1.6	55.5	59.4	66.0	73.2	80.5	87.1	91.0
1.7	62.2	66.5	73.6	81.6	89.5	96.7	101.0
1.8	68.8	73.5	81.3	90.0	98.6	106.4	111.1
1.9	75.4	80.5	89.0	98.5	108.0	116.5	121.6
2.0	82.3	87.8	97.1	107.5	117.8	127.1	132.7
2.1	89.5	95.5	105.7	117.0	128.4	138.5	144.6
2.2	96.8	103.5	114.6	126.9	139.2	150.3	157.0

### Myocardial thickness of the RV segments and RV mass

Linear regression (Table 8) showed that BSA, height, weight and age were strong predictors of the RV basal, mid-ventricular and apical myocardial thickness as well as RV mass. In addition, average heart rate was a strong predictor for myocardial mass. Gender did not predict myocardial thickness at any RV chamber level.

**Table 8:** Linear regression analysis showing impact of demographic parameters on RV myocardial thickness and mass separately for each variable

Variable	Regression coefficient	Standard error	P value
Myocardial thickness, RV base			
Age	0.070	0.0054	< 2 × 10^–16^
Men	0.048	0.046	0.30
Body surface area	0.78	0.041	< 2 × 10^–16^
Body height	0.013	0.00088	< 2 × 10^–16^
Body weight	0.014	0.00082	< 2 × 10^–16^
Average heart rate	− 0.0033	0.0014	0.018
Myocardial thickness, RV mid-cavity			
Age	0.065	0.0054	< 2 × 10^–16^
Men	0.072	0.044	0.106
Body surface area	0.73	0.041	< 2 × 10^–16^
Body height	0.012	0.00086	< 2 × 10^–16^
Body weight	0.013	0.00080	< 2 × 10^–16^
Average heart rate	− 0.0025	0.0013	0.060
Myocardial thickness, RV apex			
Age	0.054	0.0050	< 2 × 10^–16^
Men	0.072	0.039	0.066
Body surface area	0.63	0.038	< 2 × 10^–16^
Body height	0.010	0.00079	< 2 × 10^–16^
Body weight	0.011	0.00072	< 2 × 10^–16^
Average heart rate	− 0.0031	0.0012	0.0089
Myocardial mass			
Age	2.52	0.22	< 2 × 10^–16^
Men	5.77	1.70	0.00087
Body surface area	28.79	1.62	< 2 × 10^–16^
Body height	0.50	0.031	< 2 × 10^–16^
Body weight	0.49	0.034	< 2 × 10^–16^
Average heart rate	− 0.24	0.050	4.3 × 10^–6^

In multiple regression analysis (Table 9), BSA showed a strong impact on basal and mid-ventricular wall thickness as well as mass for girls whereas for boys, body height and body weight yielded a better model fit. Body weight showed a strong impact on apical wall thickness in girls, whereas for boys BSA was a better predictor.

**Table 9:** Multiple regression analysis showing impact of demographic parameters on RV myocardial thickness and myocardial mass

Variable	Regression coefficient	Standard error	P value	Regressioncoefficient	Standard error	P value
	Girls			Boys		
Myocardial thickness at basal right ventricle						
Body surface area	0.77	0.075	5.8 × 10^–15^	–	–	–
Body height	–	–	–	0.0074	0.0016	1.5 × 10^–5^
Body weight	–	–	–	0.0077	0.0017	2.5 × 10^–5^
Myocardial thickness at mid-ventricular right ventricle						
Body surface area	0.73	0.077	1.5 × 10^–13^	–	–	–
Body weight	–	–	–	0.0084	0.0017	2.5 × 10^–06^
Body height	–	–	–	0.0057	0.0016	0.00044
Myocardial thickness at apical right ventricle						
Body surface area	–	–	–	0.64	0.044	< 2 × 10^–16^
Body weight	0.0099	0.0012	2.6 × 10^–11^	–	–	–
Myocardial mass						
Body surface area	23.15	2.57	7.6 × 10^–13^	–	–	–
Body height	–	–	–	0.39	0.059	1.9 × 10^–9^
Body weight	–	–	–	0.19	0.063	0.0027

Only significant results are displayed

**Fig. 8:**
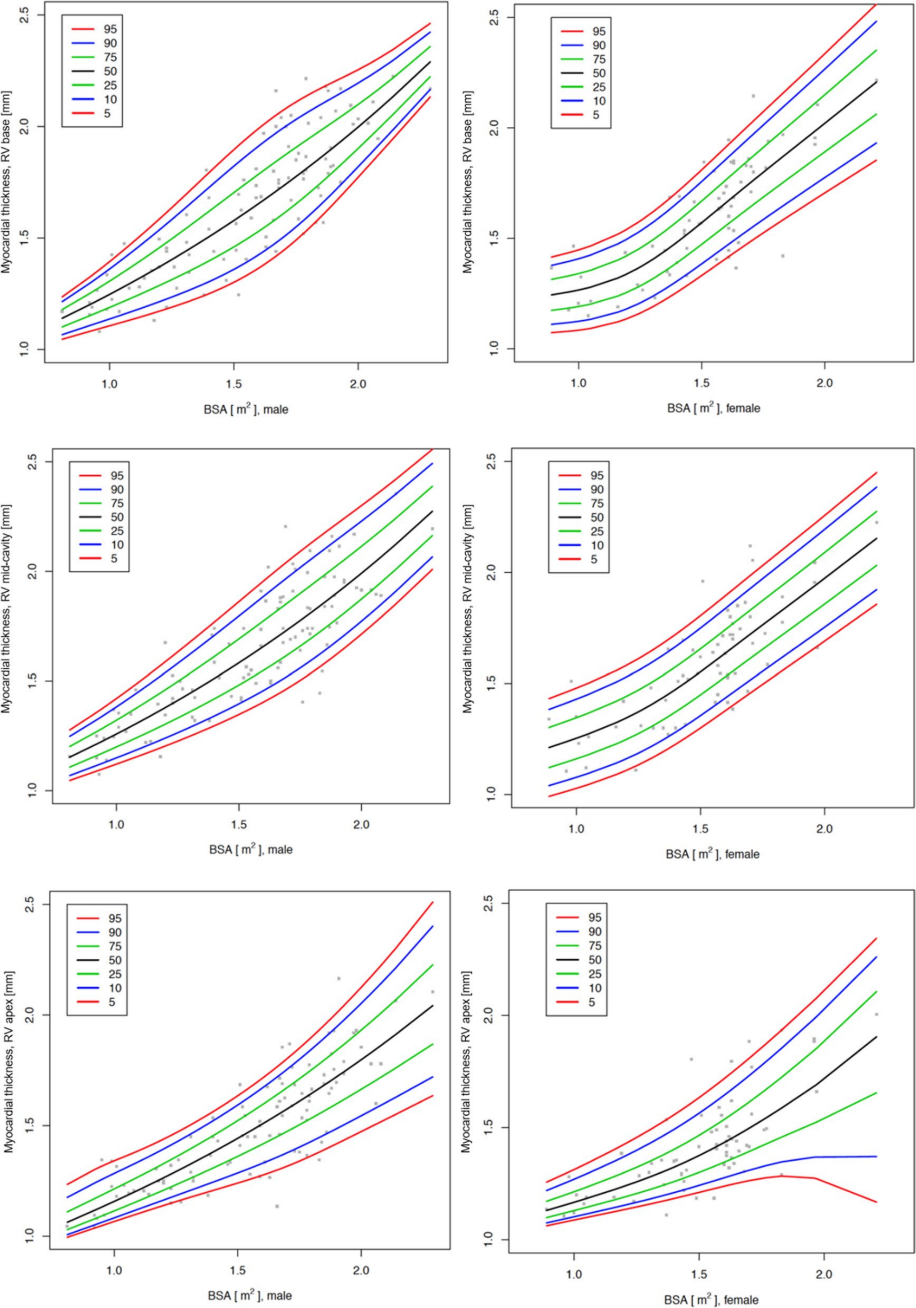
Centile charts showing myocardial thickness at RV base, mid-cavity and apex by body surface area (BSA) and gender. Colors correspond to the given centiles

**Fig. 9:**
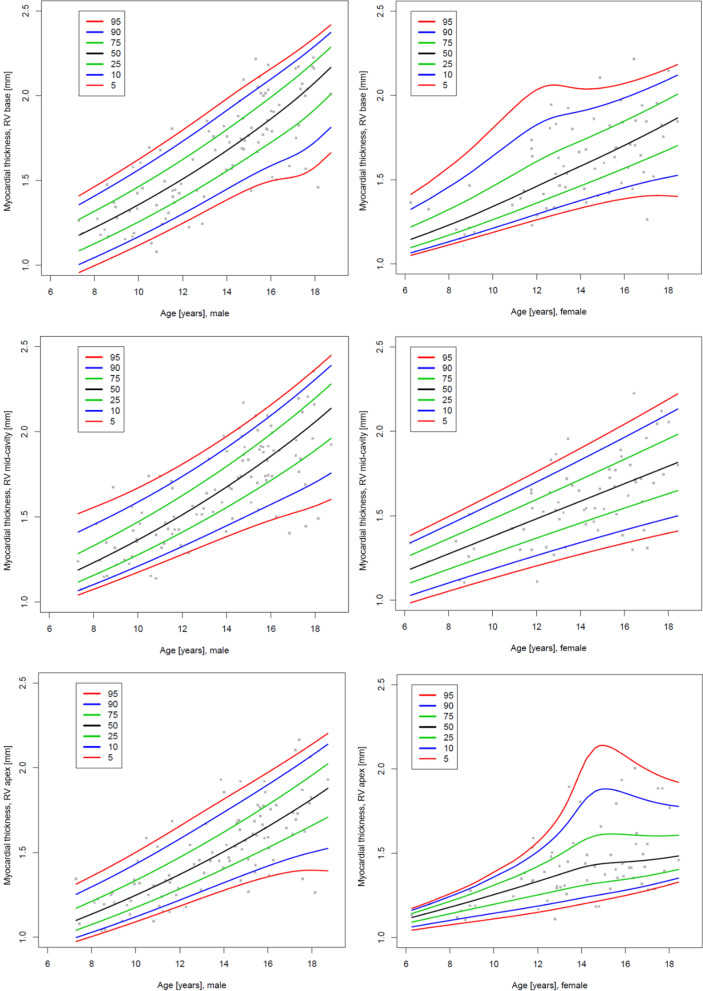
Centile charts showing myocardial thickness at RV base, mid-cavity and apex by age and gender. Colors correspond to the given centiles

Figures 5, 8 and 9 show the centile charts of the myocardial thickness of the RV at the basal, mid and apical level as well of the RV myocardial mass by BSA and gender; Tables 10, 11, 12 and 13 show the centiles of the RV myocardial thickness and mass. Figures and Tables showing myocardial thickness centiles separately for lateral and inferior free wall are in Additional files 1 and 2. Centiles of the myocardial thickness of the RV separately for each measured segment across the ages broken down into boys and girls are also shown in Additional files 1 and 2. We also display centile charts for RV myocardial thickness and mass by age and gender (Additional file 2: Figure S4 and S9).

**Table 10:** Centiles of the myocardial thickness in mm of the RV at the basal level by BSA and gender

BSA (m^2^)	5th centile	10th centile	25th centile	50th centile	75th centile	90th centile	95th centile
Boys							
0.8	1.0	1.1	1.1	1.1	1.2	1.2	1.2
0.9	1.1	1.1	1.1	1.2	1.2	1.3	1.3
1.0	1.1	1.1	1.2	1.3	1.3	1.4	1.4
1.1	1.1	1.2	1.2	1.3	1.4	1.5	1.5
1.2	1.2	1.2	1.3	1.4	1.5	1.5	1.6
1.3	1.2	1.3	1.3	1.4	1.5	1.6	1.7
1.4	1.3	1.3	1.4	1.5	1.6	1.7	1.8
1.5	1.3	1.4	1.5	1.6	1.7	1.8	1.9
1.6	1.4	1.4	1.5	1.7	1.8	1.9	2.0
1.7	1.4	1.5	1.6	1.7	1.9	2.0	2.1
1.8	1.5	1.6	1.7	1.8	1.9	2.1	2.1
1.9	1.7	1.7	1.8	1.9	2.0	2.1	2.2
2.0	1.8	1.8	1.9	2.0	2.1	2.2	2.3
2.1	1.9	1.9	2.0	2.1	2.2	2.3	2.3
2.2	2.0	2.1	2.1	2.2	2.3	2.4	2.4
Girls							
0.8	1.1	1.1	1.2	1.2	1.3	1.4	1.4
0.9	1.1	1.1	1.2	1.3	1.3	1.4	1.4
1.0	1.1	1.1	1.2	1.3	1.3	1.4	1.5
1.1	1.1	1.2	1.2	1.3	1.4	1.4	1.5
1.2	1.1	1.2	1.3	1.3	1.4	1.5	1.5
1.3	1.2	1.2	1.3	1.4	1.5	1.6	1.6
1.4	1.3	1.3	1.4	1.5	1.6	1.7	1.7
1.5	1.3	1.4	1.5	1.6	1.7	1.8	1.8
1.6	1.4	1.5	1.6	1.7	1.8	1.9	1.9
1.7	1.5	1.6	1.6	1.8	1.9	2.0	2.0
1.8	1.6	1.6	1.7	1.8	2.0	2.1	2.1
1.9	1.6	1.7	1.8	1.9	2.1	2.2	2.2
2.0	1.7	1.8	1.9	2.0	2.2	2.3	2.3
2.1	1.8	1.9	2.0	2.1	2.3	2.4	2.4
2.2	1.9	1.9	2.1	2.2	2.3	2.5	2.6

**Table 11:** Centiles of the myocardial thickness in mm of the RV at the mid-ventricular level by BSA and gender

BSA (m^2^)	5th centile	10th centile	25th centile	50th centile	75th centile	90th centile	95th centile
Boys							
0.8	1.0	1.1	1.1	1.2	1.2	1.3	1.3
0.9	1.1	1.1	1.2	1.2	1.3	1.4	1.4
1.0	1.1	1.1	1.2	1.3	1.4	1.4	1.5
1.1	1.2	1.2	1.3	1.3	1.4	1.5	1.6
1.2	1.2	1.2	1.3	1.4	1.5	1.6	1.6
1.3	1.2	1.3	1.4	1.5	1.6	1.7	1.7
1.4	1.3	1.4	1.4	1.5	1.7	1.8	1.8
1.5	1.3	1.4	1.5	1.6	1.7	1.8	1.9
1.6	1.4	1.5	1.6	1.7	1.8	1.9	2.0
1.7	1.5	1.5	1.6	1.8	1.9	2.0	2.1
1.8	1.5	1.6	1.7	1.9	2.0	2.1	2.2
1.9	1.6	1.6	1.8	1.9	2.1	2.3	2.3
2.0	1.6	1.7	1.9	2.0	2.2	2.4	2.5
2.1	1.7	1.8	1.9	2.1	2.3	2.5	2.6
2.2	1.8	1.9	2.0	2.2	2.4	2.6	2.7
Girls							
0.8	1.0	1.1	1.2	1.2	1.3	1.4	1.5
0.9	1.0	1.1	1.2	1.3	1.4	1.4	1.5
1.0	1.0	1.1	1.2	1.3	1.4	1.5	1.5
1.1	1.1	1.1	1.2	1.3	1.4	1.5	1.5
1.2	1.1	1.1	1.2	1.3	1.4	1.5	1.6
1.3	1.1	1.2	1.3	1.4	1.5	1.6	1.7
1.4	1.2	1.3	1.4	1.5	1.6	1.7	1.8
1.5	1.3	1.4	1.5	1.6	1.7	1.8	1.9
1.6	1.4	1.5	1.6	1.7	1.8	1.9	2.0
1.7	1.5	1.6	1.7	1.8	1.9	2.0	2.1
1.8	1.6	1.6	1.7	1.9	2.0	2.1	2.2
1.9	1.6	1.7	1.8	2.0	2.1	2.2	2.3
2.0	1.7	1.8	1.9	2.0	2.2	2.3	2.4
2.1	1.8	1.9	2.0	2.1	2.3	2.4	2.5
2.2	1.9	1.9	2.1	2.2	2.4	2.5	2.6

**Table 12:** Centiles of the myocardial thickness in mm of the RV at the apical level by BSA and gender

BSA (m^2^)	5th centile	10th centile	25th centile	50th centile	75th centile	90th centile	95th centile
Boys							
0.8	1.0	1.0	1.0	1.1	1.1	1.2	1.2
0.9	1.0	1.0	1.1	1.1	1.2	1.2	1.3
1.0	1.1	1.1	1.1	1.2	1.2	1.3	1.3
1.1	1.1	1.1	1.2	1.2	1.3	1.3	1.4
1.2	1.1	1.2	1.2	1.3	1.3	1.4	1.4
1.3	1.2	1.2	1.3	1.3	1.4	1.5	1.5
1.4	1.2	1.2	1.3	1.4	1.5	1.5	1.6
1.5	1.2	1.3	1.4	1.4	1.5	1.6	1.6
1.6	1.3	1.3	1.4	1.5	1.6	1.7	1.7
1.7	1.3	1.4	1.5	1.6	1.7	1.8	1.8
1.8	1.4	1.4	1.5	1.7	1.8	1.9	1.9
1.9	1.4	1.5	1.6	1.7	1.8	2.0	2.0
2.0	1.5	1.5	1.7	1.8	1.9	2.1	2.1
2.1	1.5	1.6	1.7	1.9	2.0	2.2	2.3
2.2	1.6	1.7	1.8	2.0	2.1	2.3	2.4
Girls							
0.8	1.0	1.1	1.1	1.1	1.1	1.2	1.2
0.9	1.1	1.1	1.1	1.1	1.2	1.2	1.3
1.0	1.1	1.1	1.1	1.2	1.2	1.3	1.3
1.1	1.1	1.1	1.2	1.2	1.3	1.3	1.4
1.2	1.1	1.2	1.2	1.2	1.3	1.4	1.4
1.3	1.2	1.2	1.2	1.3	1.4	1.4	1.5
1.4	1.2	1.2	1.3	1.3	1.4	1.5	1.6
1.5	1.2	1.2	1.3	1.4	1.5	1.6	1.6
1.6	1.2	1.3	1.3	1.4	1.5	1.6	1.7
1.7	1.3	1.3	1.4	1.5	1.6	1.7	1.8
1.8	1.3	1.3	1.4	1.6	1.7	1.8	1.9
1.9	1.3	1.4	1.5	1.6	1.8	1.9	2.0
2.0	1.3	1.4	1.5	1.7	1.9	2.0	2.1
2.1	1.2	1.4	1.6	1.8	2.0	2.1	2.2
2.2	1.2	1.4	1.7	1.9	2.1	2.3	2.3

**Table 13:** Centiles of the myocardial mass of the RV by BSA and gender

BSA (m^2^)	5th centile	10th centile	25th centile	50th centile	75th centile	90th centile	95th centile
Boys							
0.8	9.6	10.8	12.8	15.0	17.3	19.3	20.4
0.9	11.6	12.9	15.0	17.4	19.8	22.0	23.3
1.0	13.5	14.9	17.2	19.8	22.4	24.7	26.1
1.1	15.5	17.0	19.5	22.3	25.1	27.6	29.1
1.2	17.6	19.2	21.9	24.9	27.9	30.6	32.2
1.3	19.8	21.5	24.4	27.7	30.9	33.8	35.5
1.4	22.1	24.0	27.2	30.6	34.1	37.2	39.1
1.5	24.7	26.7	30.1	33.8	37.6	40.9	43.0
1.6	27.3	29.5	33.1	37.2	41.2	44.8	47.0
1.7	29.9	32.3	36.2	40.6	44.9	48.8	51.2
1.8	32.4	35.0	39.2	43.9	48.6	52.8	55.3
1.9	34.8	37.5	42.0	47.1	52.1	56.7	59.4
2.0	36.9	39.8	44.7	50.2	55.6	60.5	63.5
2.1	38.9	42.0	47.3	53.2	59.0	64.3	67.5
2.2	40.7	44.1	49.8	56.1	62.5	68.2	71.6
Girls							
0.8	10.2	11.2	12.8	14.6	16.3	18.0	18.9
0.9	12.0	13.0	14.8	16.8	18.7	20.5	21.6
1.0	13.7	14.9	16.8	19.0	21.1	23.1	24.2
1.1	15.5	16.7	18.9	21.2	23.6	25.7	27.0
1.2	17.1	18.5	20.8	23.4	26.0	28.3	29.7
1.3	18.8	20.3	22.8	25.6	28.5	31.0	32.5
1.4	20.3	22.0	24.8	27.9	31.0	33.7	35.4
1.5	21.8	23.6	26.7	30.1	33.5	36.5	38.3
1.6	23.3	25.3	28.6	32.3	36.0	39.4	41.3
1.7	24.6	26.8	30.6	34.5	38.6	42.2	44.4
1.8	25.9	28.3	32.3	36.7	41.2	45.2	47.6
1.9	27.1	29.7	34.1	39.0	43.8	48.2	50.8
2.0	28.2	31.1	35.9	41.2	46.5	51.3	54.2
2.1	29.1	32.3	37.6	43.4	49.2	54.5	57.6
2.2	30.0	33.5	39.2	45.6	52.0	57.8	61.2

### Interobserver variability

The inter-observer ICC for basal myocardial thickness measurements was 0.815 (95% CI 0.638, 0.909), for mid-ventricular 0.883 (95% CI 0.757, 0.944) and for apical measurements 0.860 (95% CI 0.728, 0.930).

## Discussion

Assessment of myocardial thickness is important for many cardiovascular diseases already in childhood. CMR is increasingly used in pediatric patients for detailed global and regional myocardial characterization but normative biventricular data for myocardial thickness in children and adolescents are lacking.

In the current study, we present normal segmental myocardial thickness values of 16 LV and 6 RV segments as well as normal values for myocardial mass in children and adolescents between 6 and 18 years. BSA was found to be the major determinant factor of the segmental myocardial thickness in childhood. Therefore, centile charts and tables for all segments of both ventricles and for myocardial mass were established primarily with respect to BSA and gender and only for completeness with respect to age and gender.

Linear regression showed associations of the segmental myocardial thickness with all studied variables (except average heart frequency), i.e. with age, weight, height and gender for LV segments and the same, except for gender, for RV segments. This difference might be caused by the overall much thinner RV thickness (0.9–2.7 mm) and the inability to distinguish between very small differences given the spatial resolution of the bSSFP cine images (voxel size 1.6 × 0.6 × 8.0 mm). In multiple regression analysis, body surface area was the strongest determinant for the majority of the segments and BSA correlated strongly with weight or height. We, therefore, include centile charts and tables that show wall thickness as a function of BSA.

LV myocardial thickness has been measured in a large study of healthy middle-aged adults. Similar to our study, the mean LV myocardial thickness was found to be positively associated with BSA and also weight. No relationship was detected between mean LV myocardial thickness and age or height [17]. In another study, the myocardial thickness increased after the fourth decade. This study measured also the size of the trabeculated layer in all segments and total LV myocardial thickness and it was found that the size of the trabeculated layer decreased with age whereas the thickness of the total myocardial layer remained unchanged [23]. Our study does not only provide normal values for myocardial thickness but also for myocardial mass in the pediatric age group. Compared to previous CMR studies about normal myocardial mass ranges, we included a larger cohort [9–11, 13].

Tables and charts were created by the Lambda-Mu-Sigma (LMS) model introduced by Cole and Green [20]. This model can be applied, and is frequently used, when the centiles change according to some covariate, which is often age, but can also be another variable like BSA for our charts and tables. The parameters of the LMS model, which capture the variation of the centiles, are median, coefficient of variation and skewness. All measurements in this study were performed from conventional bSSFP cine sequences widely used in the pediatric CMR imaging. This study did not compare images from gradient echo (GRE) sequences or real time sequences with bSSFP images and therefore, measurements of myocardial thickness using those sequences cannot directly be transferred to our centile charts and tables. Type of the sequence (bSSFP vs GRE) has been shown to cause variation in volumes and mass measurements in adult population. In particular, EDV and ESV are larger and mass is smaller when analyzed from bSSFP images when compared to GRE sequence and a linear relationship exists for these parameters between both sequences [24, 25]. This can be explained by much more distinct endo- and epicardial borders in both ventricles when bSSFP sequence is used. GRE and especially real time sequences might cause higher measurements and much higher intra- and interobserver variability and therefore, another study analyzing myocardial wall thickness using these sequences would be necessary. Interestingly, no difference has been found for measurements of volumes and mass when comparing results from 1.5 T and 3 T scanners using the same type of sequence [26]. This could potentially be the same for myocardial thickness but needs to be validated. However, from our experience GRE sequences are often necessary in children after congenital heart disease surgery and after interventional cardiac catheterization procedures due to frequent artefacts when using bSSFP sequences.

Interobserver variability demonstrated good agreement for basal, midventricular and apical and this was comparable with a previous published study [17].

All measurements in the current study were performed on short axis images. Comparison has been made between measurements of myocardial thickness in different planes in the adult population and the myocardial thickness was found to be 6% higher at basal level, 10% higher at mid-ventricular level and 20% lower at apical level on long axis images compared to short axis images [17]. Therefore, normal values presented in the current study should not be used as standard for any other but short axis views.

### Study limitations

This is a retrospective study with associated limitations. The study includes only children from the age of 6 and 18 years as there were not enough younger healthy children who underwent CMR. This is typically because children younger than 6 years usually require general anesthesia or sedation and therefore, the indication for the scan is much stricter than for awake scans performed in older children. In addition, the numbers of included children and adolescents with an age of 6–8 years and an age of 18 years are small. However, the applied statistical methods model a general trend over the whole age range. Therefore, good results can even be achieved for age groups with low sample size. Nevertheless, the estimation accuracy is smaller in those groups.

The provided normal values are those for European population and cannot necessarily be used for children from other populations.

With development of real time imaging, which is more often used in uncooperative children, another problem arises with regard to interpretation of the myocardial thickness measurements. Further studies would be needed to investigate if segmental myocardial thickness can be measured reliably from these sequences.

### Conclusions

We provide normal values for segmental myocardial thickness and mass of both ventricles, which can serve as a reference standard for the diagnosis of acquired and congenital heart disease in children and adolescents. BSA was the major determinant of the myocardial thickness and mass for both ventricles.

## Supplementary Information

**Additional file 1.** Additional tables.

**Additional file 2.** Additional figures.

## Data Availability

The datasets used and analysed during the current study are available from the corresponding author on reasonable request.

## Abbreviations

BSA: Body surface area
bSSFP: Balanced steady state free precession
CMR: Cardiovascular magnetic resonance
EDV: End-diastolic volume
EDVI: End-diastolic volume index
EF: Ejection fraction
ESV: End-systolic volume
ESVI: End-systolic volume index
GRE: Gradient recalled echo
LV: Left ventricle/left ventricular
LVEDV: Left ventricular end-diastolic volume
LVEF: Left ventricular ejection fraction
LVESV: Left ventricular end-systolic volume
LVSV: Left ventricular stroke volume
RV: Right ventricle/right ventricular
RVEDV: Right ventricular end-diastolic volume
RVEF: Right ventricular ejection fraction
RVESV: Right ventricular end-systolic volume
RVSV: Right ventricular stroke volume
