# Supplementary material for: Cardiovascular magnetic resonance normal values in children for biventricular wall thickness and mass
Source: J Cardiovasc Magn Reson. 2021 Jan 4;23:1. doi: 10.1186/s12968-020-00692-2 (PMC7780624; doi:10.1186/s12968-020-00692-2)
Supplement: Supplementary file 1 — Additional file 1. Additional tables. [file 12968_2020_692_MOESM1_ESM.docx]

**Table S1.** Centiles of the myocardial (presented in mm) thickness of the LV basal anterior segment (segment 1) in boys and girls by BSA.

|  |  |  | **BOYS** |  |  |  |  |
| --- | --- | --- | --- | --- | --- | --- | --- |
| **BSA (m2)** | **5th centile** | **10th centile** | **25th centile** | **50th centile** | **75th centile** | **90th centile** | **95th centile** |
| 0.8 | 2.4 | 2.6 | 2.9 | 3.4 | 3.8 | 4.1 | 4.4 |
| 0.9 | 2.5 | 2.8 | 3.2 | 3.6 | 4.0 | 4.4 | 4.6 |
| 1.0 | 2.7 | 3.0 | 3.4 | 3.8 | 4.3 | 4.7 | 4.9 |
| 1.1 | 2.9 | 3.2 | 3.6 | 4.0 | 4.5 | 4.9 | 5.2 |
| 1.2 | 3.1 | 3.3 | 3.8 | 4.3 | 4.8 | 5.2 | 5.5 |
| 1.3 | 3.2 | 3.5 | 4.0 | 4.5 | 5.0 | 5.5 | 5.8 |
| 1.4 | 3.4 | 3.7 | 4.2 | 4.7 | 5.3 | 5.8 | 6.0 |
| 1.5 | 3.6 | 3.9 | 4.4 | 5.0 | 5.5 | 6.0 | 6.3 |
| 1.6 | 3.8 | 4.1 | 4.6 | 5.2 | 5.8 | 6.3 | 6.6 |
| 1.7 | 3.9 | 4.2 | 4.8 | 5.4 | 6.0 | 6.6 | 6.9 |
| 1.8 | 4.1 | 4.4 | 5.0 | 5.6 | 6.3 | 6.9 | 7.2 |
| 1.9 | 4.2 | 4.6 | 5.2 | 5.9 | 6.6 | 7.2 | 7.5 |
| 2.0 | 4.4 | 4.8 | 5.4 | 6.1 | 6.8 | 7.5 | 7.8 |
| 2.1 | 4.5 | 4.9 | 5.6 | 6.3 | 7.1 | 7.7 | 8.1 |
| 2.2 | 4.7 | 5.1 | 5.8 | 6.6 | 7.3 | 8.0 | 8.5 |
|  |  |  | **GIRLS** |  |  |  |  |
| **BSA (m2)** | **5th centile** | **10th centile** | **25th centile** | **50th centile** | **75th centile** | **90th centile** | **95th centile** |
| 0.8 | 2.1 | 2.3 | 2.6 | 2.9 | 3.4 | 3.9 | 4.2 |
| 0.9 | 2.3 | 2.5 | 2.7 | 3.1 | 3.6 | 4.1 | 4.4 |
| 1.0 | 2.5 | 2.6 | 2.9 | 3.3 | 3.8 | 4.2 | 4.6 |
| 1.1 | 2.7 | 2.9 | 3.1 | 3.5 | 4.0 | 4.4 | 4.8 |
| 1.2 | 2.9 | 3.1 | 3.4 | 3.7 | 4.2 | 4.7 | 5.0 |
| 1.3 | 3.1 | 3.3 | 3.6 | 4.0 | 4.4 | 4.9 | 5.2 |
| 1.4 | 3.4 | 3.5 | 3.8 | 4.2 | 4.7 | 5.1 | 5.4 |
| 1.5 | 3.6 | 3.8 | 4.1 | 4.5 | 4.9 | 5.4 | 5.7 |
| 1.6 | 3.9 | 4.1 | 4.4 | 4.7 | 5.2 | 5.6 | 6.0 |
| 1.7 | 4.2 | 4.4 | 4.7 | 5.0 | 5.5 | 5.9 | 6.2 |
| 1.8 | 4.5 | 4.7 | 5.0 | 5.3 | 5.8 | 6.2 | 6.5 |
| 1.9 | 4.8 | 5.0 | 5.3 | 5.7 | 6.1 | 6.5 | 6.9 |
| 2.0 | 5.2 | 5.4 | 5.6 | 6.0 | 6.4 | 6.9 | 7.2 |
| 2.1 | 5.6 | 5.7 | 6.0 | 6.4 | 6.8 | 7.3 | 7.6 |
| 2.2 | 6.0 | 6.1 | 6.4 | 6.8 | 7.2 | 7.6 | 7.9 |

**Table S2.** Centiles of the myocardial thickness (presented in mm) of the LV basal anteroseptal segment (segment 2) in boys and girls by BSA.

|  |  |  | **BOYS** |  |  |  |  |
| --- | --- | --- | --- | --- | --- | --- | --- |
| **BSA (m2)** | **5th centile** | **10th centile** | **25th centile** | **50th centile** | **75th centile** | **90th centile** | **95th centile** |
| 0.8 | 2.5 | 2.8 | 3.2 | 3.8 | 4.3 | 4.8 | 5.0 |
| 0.9 | 2.7 | 3.0 | 3.5 | 4.0 | 4.5 | 5.0 | 5.3 |
| 1.0 | 3.0 | 3.3 | 3.7 | 4.3 | 4.8 | 5.3 | 5.5 |
| 1.1 | 3.2 | 3.5 | 4.0 | 4.5 | 5.0 | 5.5 | 5.8 |
| 1.2 | 3.5 | 3.8 | 4.2 | 4.8 | 5.3 | 5.8 | 6.0 |
| 1.3 | 3.7 | 4.0 | 4.5 | 5.0 | 5.5 | 6.0 | 6.3 |
| 1.4 | 4.0 | 4.3 | 4.7 | 5.3 | 5.8 | 6.3 | 6.6 |
| 1.5 | 4.2 | 4.5 | 5.0 | 5.5 | 6.1 | 6.5 | 6.8 |
| 1.6 | 4.5 | 4.8 | 5.3 | 5.8 | 6.3 | 6.8 | 7.1 |
| 1.7 | 4.7 | 5.0 | 5.5 | 6.0 | 6.6 | 7.0 | 7.3 |
| 1.8 | 5.0 | 5.3 | 5.8 | 6.3 | 6.8 | 7.3 | 7.6 |
| 1.9 | 5.2 | 5.5 | 6.0 | 6.5 | 7.1 | 7.6 | 7.8 |
| 2.0 | 5.5 | 5.8 | 6.2 | 6.8 | 7.3 | 7.8 | 8.1 |
| 2.1 | 5.7 | 6.0 | 6.5 | 7.0 | 7.6 | 8.1 | 8.4 |
| 2.2 | 6.0 | 6.3 | 6.7 | 7.3 | 7.8 | 8.3 | 8.6 |
|  |  |  | **GIRLS** |  |  |  |  |
| **BSA (m2)** | **5th centile** | **10th centile** | **25th centile** | **50th centile** | **75th centile** | **90th centile** | **95th centile** |
| 0.8 | 3.0 | 3.3 | 3.7 | 4.2 | 4.7 | 5.1 | 5.4 |
| 0.9 | 3.0 | 3.3 | 3.7 | 4.2 | 4.7 | 5.1 | 5.4 |
| 1.0 | 3.1 | 3.3 | 3.8 | 4.2 | 4.7 | 5.1 | 5.4 |
| 1.1 | 3.2 | 3.4 | 3.8 | 4.3 | 4.8 | 5.2 | 5.4 |
| 1.2 | 3.3 | 3.5 | 3.9 | 4.4 | 4.9 | 5.3 | 5.5 |
| 1.3 | 3.5 | 3.7 | 4.1 | 4.6 | 5.0 | 5.4 | 5.6 |
| 1.4 | 3.7 | 4.0 | 4.4 | 4.8 | 5.2 | 5.6 | 5.9 |
| 1.5 | 4.1 | 4.3 | 4.7 | 5.1 | 5.5 | 5.9 | 6.1 |
| 1.6 | 4.4 | 4.6 | 5.0 | 5.4 | 5.9 | 6.2 | 6.5 |
| 1.7 | 4.8 | 5.0 | 5.4 | 5.8 | 6.2 | 6.6 | 6.8 |
| 1.8 | 5.2 | 5.4 | 5.7 | 6.2 | 6.6 | 6.9 | 7.1 |
| 1.9 | 5.6 | 5.8 | 6.1 | 6.5 | 6.9 | 7.3 | 7.5 |
| 2.0 | 6.0 | 6.2 | 6.6 | 7.0 | 7.4 | 7.7 | 7.9 |
| 2.1 | 6.5 | 6.7 | 7.0 | 7.4 | 7.8 | 8.1 | 8.4 |
| 2.2 | 7.0 | 7.2 | 7.5 | 7.9 | 8.3 | 8.6 | 8.8 |

**Table S3.** Centiles of the myocardial thickness (presented in mm) of the LV basal inferoseptal segment (segment 3) in boys and girls by BSA.

|  |  |  | **BOYS** |  |  |  |  |
| --- | --- | --- | --- | --- | --- | --- | --- |
| **BSA (m2)** | **5th centile** | **10th centile** | **25th centile** | **50th centile** | **75th centile** | **90th centile** | **95th centile** |
| 0.8 | 2.0 | 2.3 | 2.8 | 3.4 | 3.9 | 4.4 | 4.7 |
| 0.9 | 2.4 | 2.6 | 3.1 | 3.6 | 4.1 | 4.5 | 4.8 |
| 1.0 | 2.6 | 2.9 | 3.3 | 3.8 | 4.3 | 4.7 | 5.0 |
| 1.1 | 2.9 | 3.2 | 3.6 | 4.0 | 4.5 | 4.9 | 5.2 |
| 1.2 | 3.2 | 3.4 | 3.8 | 4.3 | 4.7 | 5.1 | 5.3 |
| 1.3 | 3.5 | 3.7 | 4.1 | 4.5 | 4.9 | 5.3 | 5.5 |
| 1.4 | 3.7 | 4.0 | 4.3 | 4.7 | 5.2 | 5.5 | 5.7 |
| 1.5 | 4.0 | 4.2 | 4.6 | 5.0 | 5.4 | 5.7 | 6.0 |
| 1.6 | 4.2 | 4.4 | 4.8 | 5.2 | 5.6 | 6.0 | 6.2 |
| 1.7 | 4.4 | 4.6 | 5.0 | 5.4 | 5.9 | 6.2 | 6.5 |
| 1.8 | 4.6 | 4.8 | 5.2 | 5.7 | 6.1 | 6.5 | 6.8 |
| 1.9 | 4.7 | 5.0 | 5.4 | 5.9 | 6.4 | 6.8 | 7.1 |
| 2.0 | 4.9 | 5.1 | 5.6 | 6.1 | 6.6 | 7.1 | 7.4 |
| 2.1 | 5.0 | 5.3 | 5.8 | 6.4 | 6.9 | 7.4 | 7.7 |
| 2.2 | 5.1 | 5.4 | 6.0 | 6.6 | 7.2 | 7.8 | 8.1 |
|  |  |  | **GIRLS** |  |  |  |  |
| **BSA (m2)** | **5th centile** | **10th centile** | **25th centile** | **50th centile** | **75th centile** | **90th centile** | **95th centile** |
| 0.8 | 2.0 | 2.3 | 2.7 | 3.3 | 3.8 | 4.2 | 4.5 |
| 0.9 | 2.2 | 2.5 | 2.9 | 3.4 | 4.0 | 4.4 | 4.7 |
| 1.0 | 2.4 | 2.7 | 3.1 | 3.6 | 4.2 | 4.6 | 4.9 |
| 1.1 | 2.6 | 2.9 | 3.3 | 3.8 | 4.4 | 4.8 | 5.1 |
| 1.2 | 2.8 | 3.1 | 3.5 | 4.0 | 4.5 | 5.0 | 5.3 |
| 1.3 | 3.0 | 3.3 | 3.7 | 4.2 | 4.7 | 5.2 | 5.5 |
| 1.4 | 3.2 | 3.5 | 3.9 | 4.4 | 4.9 | 5.4 | 5.6 |
| 1.5 | 3.5 | 3.7 | 4.1 | 4.6 | 5.1 | 5.6 | 5.8 |
| 1.6 | 3.7 | 3.9 | 4.4 | 4.8 | 5.3 | 5.7 | 6.0 |
| 1.7 | 3.9 | 4.1 | 4.6 | 5.0 | 5.5 | 5.9 | 6.2 |
| 1.8 | 4.1 | 4.3 | 4.8 | 5.2 | 5.7 | 6.1 | 6.4 |
| 1.9 | 4.3 | 4.5 | 5.0 | 5.4 | 5.9 | 6.3 | 6.6 |
| 2.0 | 4.5 | 4.7 | 5.2 | 5.6 | 6.1 | 6.5 | 6.8 |
| 2.1 | 4.7 | 5.0 | 5.4 | 5.8 | 6.3 | 6.7 | 6.9 |
| 2.2 | 4.9 | 5.2 | 5.6 | 6.0 | 6.5 | 6.9 | 7.1 |

**Table S4.** Centiles of the myocardial thickness (presented in mm) of the LV basal inferior segment (segment 4) in boys and girls by BSA.

|  |  |  | **BOYS** |  |  |  |  |
| --- | --- | --- | --- | --- | --- | --- | --- |
| **BSA (m2)** | **5th centile** | **10th centile** | **25th centile** | **50th centile** | **75th centile** | **90th centile** | **95th centile** |
| 0.8 | 2.3 | 2.5 | 2.9 | 3.4 | 3.8 | 4.2 | 4.5 |
| 0.9 | 2.5 | 2.8 | 3.2 | 3.6 | 4.1 | 4.5 | 4.7 |
| 1.0 | 2.7 | 3.0 | 3.4 | 3.9 | 4.3 | 4.7 | 5.0 |
| 1.1 | 2.9 | 3.2 | 3.6 | 4.1 | 4.6 | 5.0 | 5.3 |
| 1.2 | 3.1 | 3.4 | 3.8 | 4.3 | 4.8 | 5.3 | 5.5 |
| 1.3 | 3.3 | 3.6 | 4.0 | 4.6 | 5.1 | 5.5 | 5.8 |
| 1.4 | 3.5 | 3.8 | 4.3 | 4.8 | 5.3 | 5.8 | 6.1 |
| 1.5 | 3.7 | 4.0 | 4.5 | 5.0 | 5.6 | 6.1 | 6.3 |
| 1.6 | 3.9 | 4.2 | 4.7 | 5.3 | 5.8 | 6.3 | 6.6 |
| 1.7 | 4.1 | 4.4 | 4.9 | 5.5 | 6.1 | 6.6 | 6.9 |
| 1.8 | 4.3 | 4.6 | 5.1 | 5.7 | 6.3 | 6.8 | 7.2 |
| 1.9 | 4.5 | 4.8 | 5.4 | 6.0 | 6.6 | 7.1 | 7.4 |
| 2.0 | 4.7 | 5.0 | 5.6 | 6.2 | 6.8 | 7.4 | 7.7 |
| 2.1 | 4.9 | 5.2 | 5.8 | 6.4 | 7.1 | 7.7 | 8.0 |
| 2.2 | 5.0 | 5.4 | 6.0 | 6.7 | 7.3 | 7.9 | 8.3 |
|  |  |  | **GIRLS** |  |  |  |  |
| **BSA (m2)** | **5th centile** | **10th centile** | **25th centile** | **50th centile** | **75th centile** | **90th centile** | **95th centile** |
| 0.8 | 1.2 | 1.6 | 2.2 | 2.9 | 3.6 | 4.2 | 4.5 |
| 0.9 | 1.5 | 1.9 | 2.5 | 3.2 | 3.8 | 4.4 | 4.8 |
| 1.0 | 1.8 | 2.2 | 2.8 | 3.4 | 4.1 | 4.7 | 5.0 |
| 1.1 | 2.1 | 2.5 | 3.1 | 3.7 | 4.4 | 4.9 | 5.3 |
| 1.2 | 2.4 | 2.8 | 3.4 | 4.0 | 4.6 | 5.2 | 5.5 |
| 1.3 | 2.8 | 3.1 | 3.6 | 4.3 | 4.9 | 5.4 | 5.8 |
| 1.4 | 3.1 | 3.4 | 3.9 | 4.5 | 5.1 | 5.7 | 6.0 |
| 1.5 | 3.4 | 3.7 | 4.2 | 4.8 | 5.4 | 5.9 | 6.3 |
| 1.6 | 3.7 | 4.0 | 4.5 | 5.1 | 5.7 | 6.2 | 6.5 |
| 1.7 | 4.0 | 4.3 | 4.8 | 5.4 | 5.9 | 6.5 | 6.8 |
| 1.8 | 4.3 | 4.6 | 5.1 | 5.6 | 6.2 | 6.7 | 7.0 |
| 1.9 | 4.6 | 4.9 | 5.4 | 5.9 | 6.5 | 7.0 | 7.3 |
| 2.0 | 4.9 | 5.2 | 5.6 | 6.2 | 6.7 | 7.2 | 7.5 |
| 2.1 | 5.2 | 5.5 | 5.9 | 6.5 | 7.0 | 7.5 | 7.8 |
| 2.2 | 5.5 | 5.7 | 6.2 | 6.7 | 7.3 | 7.7 | 8.0 |

**Table S5.** Centiles of the myocardial thickness (presented in mm) of the LV basal inferolateral segment (segment 5) in boys and girls by BSA.

|  |  |  | **BOYS** |  |  |  |  |
| --- | --- | --- | --- | --- | --- | --- | --- |
| **BSA (m2)** | **5th centile** | **10th centile** | **25th centile** | **50th centile** | **75th centile** | **90th centile** | **95th centile** |
| 0.8 | 1.8 | 2.1 | 2.7 | 3.4 | 4.0 | 4.6 | 5.0 |
| 0.9 | 2.1 | 2.5 | 3.0 | 3.7 | 4.4 | 4.9 | 5.3 |
| 1.0 | 2.4 | 2.8 | 3.4 | 4.0 | 4.7 | 5.3 | 5.6 |
| 1.1 | 2.7 | 3.1 | 3.7 | 4.3 | 5.0 | 5.6 | 5.9 |
| 1.2 | 3.0 | 3.4 | 3.9 | 4.6 | 5.2 | 5.8 | 6.2 |
| 1.3 | 3.3 | 3.6 | 4.2 | 4.8 | 5.5 | 6.1 | 6.4 |
| 1.4 | 3.5 | 3.9 | 4.5 | 5.1 | 5.8 | 6.3 | 6.7 |
| 1.5 | 3.8 | 4.2 | 4.8 | 5.4 | 6.1 | 6.6 | 7.0 |
| 1.6 | 4.2 | 4.5 | 5.1 | 5.8 | 6.4 | 7.0 | 7.3 |
| 1.7 | 4.6 | 4.9 | 5.5 | 6.1 | 6.8 | 7.4 | 7.7 |
| 1.8 | 4.9 | 5.2 | 5.8 | 6.4 | 7.1 | 7.7 | 8.0 |
| 1.9 | 5.1 | 5.4 | 6.0 | 6.6 | 7.3 | 7.9 | 8.2 |
| 2.0 | 5.2 | 5.5 | 6.1 | 6.7 | 7.4 | 8.0 | 8.3 |
| 2.1 | 5.2 | 5.5 | 6.1 | 6.8 | 7.4 | 8.0 | 8.3 |
| 2.2 | 5.2 | 5.5 | 6.1 | 6.7 | 7.4 | 7.9 | 8.3 |
|  |  |  | **GIRLS** |  |  |  |  |
| **BSA (m2)** | **5th centile** | **10th centile** | **25th centile** | **50th centile** | **75th centile** | **90th centile** | **95th centile** |
| 0.8 | 1.2 | 1.6 | 2.2 | 2.9 | 3.6 | 4.2 | 4.5 |
| 0.9 | 1.5 | 1.9 | 2.5 | 3.2 | 3.8 | 4.4 | 4.8 |
| 1.0 | 1.8 | 2.2 | 2.8 | 3.4 | 4.1 | 4.7 | 5.0 |
| 1.1 | 2.1 | 2.5 | 3.1 | 3.7 | 4.4 | 4.9 | 5.3 |
| 1.2 | 2.4 | 2.8 | 3.4 | 4.0 | 4.6 | 5.2 | 5.5 |
| 1.3 | 2.8 | 3.1 | 3.6 | 4.3 | 4.9 | 5.4 | 5.8 |
| 1.4 | 3.1 | 3.4 | 3.9 | 4.5 | 5.1 | 5.7 | 6.0 |
| 1.5 | 3.4 | 3.7 | 4.2 | 4.8 | 5.4 | 5.9 | 6.3 |
| 1.6 | 3.7 | 4.0 | 4.5 | 5.1 | 5.7 | 6.2 | 6.5 |
| 1.7 | 4.0 | 4.3 | 4.8 | 5.4 | 5.9 | 6.5 | 6.8 |
| 1.8 | 4.3 | 4.6 | 5.1 | 5.6 | 6.2 | 6.7 | 7.0 |
| 1.9 | 4.6 | 4.9 | 5.4 | 5.9 | 6.5 | 7.0 | 7.3 |
| 2.0 | 4.9 | 5.2 | 5.6 | 6.2 | 6.7 | 7.2 | 7.5 |
| 2.1 | 5.2 | 5.5 | 5.9 | 6.5 | 7.0 | 7.5 | 7.8 |
| 2.2 | 5.5 | 5.7 | 6.2 | 6.7 | 7.3 | 7.7 | 8.0 |

**Table S6.** Centiles of the myocardial thickness (presented in mm) of the LV basal anterolateral segment (segment 6) in boys by BSA. Of note, it was not possible to create a model for girls by BSA, however age related Table Sand graph for this segment is available in Supplement.

|  |  |  | **BOYS** |  |  |  |  |
| --- | --- | --- | --- | --- | --- | --- | --- |
| **BSA (m2)** | **5th centile** | **10th centile** | **25th centile** | **50th centile** | **75th centile** | **90th centile** | **95th centile** |
| 0.8 | 2.0 | 2.3 | 2.7 | 3.3 | 3.8 | 4.3 | 4.5 |
| 0.9 | 2.2 | 2.5 | 3.0 | 3.5 | 4.0 | 4.5 | 4.8 |
| 1.0 | 2.5 | 2.8 | 3.2 | 3.8 | 4.3 | 4.8 | 5.0 |
| 1.1 | 2.7 | 3.0 | 3.5 | 4.0 | 4.5 | 5.0 | 5.3 |
| 1.2 | 3.0 | 3.3 | 3.7 | 4.3 | 4.8 | 5.3 | 5.5 |
| 1.3 | 3.2 | 3.5 | 4.0 | 4.5 | 5.0 | 5.5 | 5.8 |
| 1.4 | 3.5 | 3.8 | 4.2 | 4.8 | 5.3 | 5.8 | 6.1 |
| 1.5 | 3.7 | 4.0 | 4.5 | 5.0 | 5.6 | 6.0 | 6.3 |
| 1.6 | 4.0 | 4.3 | 4.8 | 5.3 | 5.8 | 6.3 | 6.6 |
| 1.7 | 4.3 | 4.5 | 5.0 | 5.5 | 6.1 | 6.5 | 6.8 |
| 1.8 | 4.5 | 4.8 | 5.3 | 5.8 | 6.3 | 6.8 | 7.1 |
| 1.9 | 4.8 | 5.0 | 5.5 | 6.0 | 6.6 | 7.0 | 7.3 |
| 2.0 | 5.0 | 5.3 | 5.8 | 6.3 | 6.8 | 7.3 | 7.6 |
| 2.1 | 5.3 | 5.6 | 6.0 | 6.5 | 7.1 | 7.5 | 7.8 |
| 2.2 | 5.5 | 5.8 | 6.3 | 6.8 | 7.3 | 7.8 | 8.1 |

**Table S7.** Centiles of the myocardial thickness (presented in mm) of the LV midventricular anterior segment (segment 7) in boys and girls by BSA.

|  |  |  | **BOYS** |  |  |  |  |
| --- | --- | --- | --- | --- | --- | --- | --- |
| **BSA (m2)** | **5th centile** | **10th centile** | **25th centile** | **50th centile** | **75th centile** | **90th centile** | **95th centile** |
| 0.8 | 2.0 | 2.1 | 2.3 | 2.5 | 2.8 | 3.0 | 3.1 |
| 0.9 | 2.1 | 2.2 | 2.5 | 2.7 | 3.0 | 3.3 | 3.4 |
| 1.0 | 2.2 | 2.3 | 2.6 | 2.9 | 3.2 | 3.5 | 3.7 |
| 1.1 | 2.3 | 2.5 | 2.8 | 3.1 | 3.5 | 3.8 | 4.0 |
| 1.2 | 2.4 | 2.6 | 2.9 | 3.3 | 3.7 | 4.0 | 4.2 |
| 1.3 | 2.5 | 2.7 | 3.1 | 3.5 | 3.9 | 4.3 | 4.5 |
| 1.4 | 2.6 | 2.8 | 3.2 | 3.7 | 4.2 | 4.6 | 4.8 |
| 1.5 | 2.7 | 2.9 | 3.4 | 3.9 | 4.4 | 4.8 | 5.1 |
| 1.6 | 2.8 | 3.1 | 3.6 | 4.1 | 4.6 | 5.0 | 5.3 |
| 1.7 | 3.0 | 3.3 | 3.7 | 4.3 | 4.8 | 5.2 | 5.5 |
| 1.8 | 3.2 | 3.5 | 3.9 | 4.4 | 5.0 | 5.4 | 5.7 |
| 1.9 | 3.5 | 3.7 | 4.2 | 4.6 | 5.1 | 5.6 | 5.8 |
| 2.0 | 3.7 | 4.0 | 4.4 | 4.8 | 5.3 | 5.7 | 5.9 |
| 2.1 | 4.0 | 4.2 | 4.6 | 5.0 | 5.4 | 5.8 | 6.1 |
| 2.2 | 4.2 | 4.5 | 4.8 | 5.2 | 5.6 | 6.0 | 6.2 |
|  |  |  | **GIRLS** |  |  |  |  |
| **BSA (m2)** | **5th centile** | **10th centile** | **25th centile** | **50th centile** | **75th centile** | **90th centile** | **95th centile** |
| 0.8 | 1.8 | 2.1 | 2.6 | 3.1 | 3.6 | 4.0 | 4.3 |
| 0.9 | 2.0 | 2.3 | 2.7 | 3.2 | 3.7 | 4.2 | 4.5 |
| 1.0 | 2.1 | 2.4 | 2.9 | 3.4 | 3.9 | 4.4 | 4.7 |
| 1.1 | 2.3 | 2.6 | 3.1 | 3.6 | 4.1 | 4.6 | 4.9 |
| 1.2 | 2.4 | 2.7 | 3.2 | 3.8 | 4.3 | 4.8 | 5.1 |
| 1.3 | 2.6 | 2.9 | 3.4 | 3.9 | 4.5 | 5.0 | 5.3 |
| 1.4 | 2.7 | 3.0 | 3.5 | 4.1 | 4.7 | 5.2 | 5.5 |
| 1.5 | 2.8 | 3.2 | 3.7 | 4.3 | 4.9 | 5.4 | 5.8 |
| 1.6 | 3.0 | 3.3 | 3.9 | 4.5 | 5.1 | 5.6 | 6.0 |
| 1.7 | 3.1 | 3.5 | 4.0 | 4.7 | 5.3 | 5.9 | 6.2 |
| 1.8 | 3.3 | 3.6 | 4.2 | 4.8 | 5.5 | 6.1 | 6.4 |
| 1.9 | 3.4 | 3.8 | 4.3 | 5.0 | 5.7 | 6.3 | 6.6 |
| 2.0 | 3.5 | 3.9 | 4.5 | 5.2 | 5.9 | 6.5 | 6.8 |
| 2.1 | 3.7 | 4.0 | 4.7 | 5.4 | 6.1 | 6.7 | 7.1 |
| 2.2 | 3.8 | 4.2 | 4.8 | 5.5 | 6.3 | 6.9 | 7.3 |

**Table S8.** Centiles of the myocardial thickness (presented in mm) of the LV midventricular anteroseptal segment (segment 8) in boys and girls by BSA.

|  |  |  | **BOYS** |  |  |  |  |
| --- | --- | --- | --- | --- | --- | --- | --- |
| **BSA (m2)** | **5th centile** | **10th centile** | **25th centile** | **50th centile** | **75th centile** | **90th centile** | **95th centile** |
| 0.8 | 3.5 | 3.6 | 3.8 | 4.0 | 4.3 | 4.5 | 4.6 |
| 0.9 | 3.5 | 3.6 | 3.9 | 4.1 | 4.4 | 4.6 | 4.7 |
| 1.0 | 3.5 | 3.6 | 3.9 | 4.2 | 4.5 | 4.7 | 4.9 |
| 1.1 | 3.5 | 3.6 | 3.9 | 4.2 | 4.6 | 4.9 | 5.0 |
| 1.2 | 3.4 | 3.6 | 4.0 | 4.3 | 4.7 | 5.0 | 5.2 |
| 1.3 | 3.4 | 3.7 | 4.0 | 4.4 | 4.8 | 5.2 | 5.4 |
| 1.4 | 3.5 | 3.7 | 4.1 | 4.6 | 5.0 | 5.4 | 5.7 |
| 1.5 | 3.6 | 3.9 | 4.3 | 4.8 | 5.3 | 5.8 | 6.0 |
| 1.6 | 3.8 | 4.1 | 4.6 | 5.1 | 5.7 | 6.2 | 6.4 |
| 1.7 | 4.0 | 4.3 | 4.9 | 5.4 | 6.0 | 6.6 | 6.9 |
| 1.8 | 4.2 | 4.6 | 5.2 | 5.8 | 6.4 | 7.0 | 7.3 |
| 1.9 | 4.5 | 4.9 | 5.5 | 6.2 | 6.9 | 7.5 | 7.8 |
| 2.0 | 4.8 | 5.2 | 5.8 | 6.6 | 7.3 | 8.0 | 8.4 |
| 2.1 | 5.0 | 5.5 | 6.2 | 7.0 | 7.8 | 8.5 | 8.9 |
| 2.2 | 5.3 | 5.8 | 6.5 | 7.4 | 8.3 | 9.1 | 9.5 |
|  |  |  | **GIRLS** |  |  |  |  |
| **BSA (m2)** | **5th centile** | **10th centile** | **25th centile** | **50th centile** | **75th centile** | **90th centile** | **95th centile** |
| 0.8 | 2.5 | 2.8 | 3.3 | 3.9 | 4.4 | 4.9 | 5.2 |
| 0.9 | 2.7 | 3.0 | 3.5 | 4.1 | 4.6 | 5.2 | 5.5 |
| 1.0 | 2.9 | 3.2 | 3.7 | 4.3 | 4.9 | 5.4 | 5.7 |
| 1.1 | 3.1 | 3.4 | 4.0 | 4.5 | 5.1 | 5.6 | 5.9 |
| 1.2 | 3.4 | 3.7 | 4.2 | 4.8 | 5.4 | 5.9 | 6.2 |
| 1.3 | 3.6 | 3.9 | 4.4 | 5.0 | 5.6 | 6.1 | 6.4 |
| 1.4 | 3.8 | 4.1 | 4.6 | 5.2 | 5.8 | 6.4 | 6.7 |
| 1.5 | 4.0 | 4.3 | 4.9 | 5.5 | 6.1 | 6.6 | 6.9 |
| 1.6 | 4.2 | 4.5 | 5.1 | 5.7 | 6.3 | 6.8 | 7.2 |
| 1.7 | 4.4 | 4.8 | 5.3 | 5.9 | 6.5 | 7.1 | 7.4 |
| 1.8 | 4.6 | 5.0 | 5.5 | 6.1 | 6.8 | 7.3 | 7.6 |
| 1.9 | 4.9 | 5.2 | 5.8 | 6.4 | 7.0 | 7.6 | 7.9 |
| 2.0 | 5.1 | 5.4 | 6.0 | 6.6 | 7.2 | 7.8 | 8.1 |
| 2.1 | 5.3 | 5.6 | 6.2 | 6.8 | 7.5 | 8.0 | 8.4 |
| 2.2 | 5.5 | 5.8 | 6.4 | 7.1 | 7.7 | 8.3 | 8.6 |

**Table S9.** Centiles of the myocardial thickness (presented in mm) of the LV midventricular inferoseptal segment (segment 9) in boys and girls by BSA.

|  |  |  | **BOYS** |  |  |  |  |
| --- | --- | --- | --- | --- | --- | --- | --- |
| **BSA (m2)** | **5th centile** | **10th centile** | **25th centile** | **50th centile** | **75th centile** | **90th centile** | **95th centile** |
| 0.8 | 3.7 | 4.0 | 4.3 | 4.6 | 4.8 | 5.0 | 5.0 |
| 0.9 | 3.6 | 3.9 | 4.3 | 4.6 | 4.8 | 4.9 | 5.0 |
| 1.0 | 3.5 | 3.8 | 4.2 | 4.5 | 4.7 | 4.9 | 5.0 |
| 1.1 | 3.4 | 3.8 | 4.2 | 4.5 | 4.8 | 4.9 | 5.0 |
| 1.2 | 3.4 | 3.8 | 4.2 | 4.6 | 4.9 | 5.1 | 5.2 |
| 1.3 | 3.5 | 3.8 | 4.3 | 4.7 | 5.0 | 5.3 | 5.4 |
| 1.4 | 3.6 | 4.0 | 4.5 | 4.9 | 5.3 | 5.6 | 5.7 |
| 1.5 | 3.7 | 4.2 | 4.7 | 5.2 | 5.7 | 6.0 | 6.2 |
| 1.6 | 4.0 | 4.4 | 5.0 | 5.6 | 6.1 | 6.5 | 6.7 |
| 1.7 | 4.3 | 4.7 | 5.3 | 5.9 | 6.4 | 6.9 | 7.2 |
| 1.8 | 4.6 | 4.9 | 5.5 | 6.2 | 6.8 | 7.4 | 7.8 |
| 1.9 | 4.9 | 5.2 | 5.8 | 6.5 | 7.3 | 8.0 | 8.5 |
| 2.0 | 5.2 | 5.5 | 6.1 | 6.8 | 7.7 | 8.7 | 9.4 |
| 2.1 | 5.6 | 5.9 | 6.5 | 7.3 | 8.3 | 9.7 | 10.8 |
| 2.2 | 6.0 | 6.3 | 6.8 | 7.7 | 9.0 | 10.9 | 12.8 |
|  |  |  | **GIRLS** |  |  |  |  |
| **BSA (m2)** | **5th centile** | **10th centile** | **25th centile** | **50th centile** | **75th centile** | **90th centile** | **95th centile** |
| 0.8 | 2.4 | 2.7 | 3.2 | 3.8 | 4.4 | 4.9 | 5.2 |
| 0.9 | 2.7 | 3.0 | 3.5 | 4.1 | 4.6 | 5.1 | 5.4 |
| 1.0 | 3.0 | 3.3 | 3.8 | 4.3 | 4.9 | 5.4 | 5.7 |
| 1.1 | 3.2 | 3.5 | 4.0 | 4.6 | 5.2 | 5.7 | 6.0 |
| 1.2 | 3.5 | 3.8 | 4.3 | 4.9 | 5.4 | 5.9 | 6.3 |
| 1.3 | 3.8 | 4.1 | 4.6 | 5.1 | 5.7 | 6.2 | 6.5 |
| 1.4 | 4.0 | 4.3 | 4.8 | 5.4 | 6.0 | 6.5 | 6.8 |
| 1.5 | 4.3 | 4.6 | 5.1 | 5.7 | 6.2 | 6.7 | 7.0 |
| 1.6 | 4.6 | 4.9 | 5.4 | 5.9 | 6.5 | 7.0 | 7.3 |
| 1.7 | 4.9 | 5.2 | 5.7 | 6.2 | 6.7 | 7.2 | 7.5 |
| 1.8 | 5.2 | 5.5 | 6.0 | 6.5 | 7.0 | 7.4 | 7.7 |
| 1.9 | 5.6 | 5.8 | 6.3 | 6.7 | 7.2 | 7.7 | 7.9 |
| 2.0 | 5.9 | 6.1 | 6.5 | 7.0 | 7.5 | 7.9 | 8.1 |
| 2.1 | 6.2 | 6.4 | 6.8 | 7.3 | 7.7 | 8.1 | 8.4 |
| 2.2 | 6.5 | 6.7 | 7.1 | 7.5 | 8.0 | 8.4 | 8.6 |

**Table S10.** Centiles of the myocardial thickness (presented in mm) of the LV midventricular inferior segment (segment 10) in boys by BSA. Of note, it was not possible to create a model for girls by BSA, however age related Table Sand graph for this segment is available in Supplement.

|  |  |  | **BOYS** |  |  |  |  |
| --- | --- | --- | --- | --- | --- | --- | --- |
| **BSA (m2)** | **5th centile** | **10th centile** | **25th centile** | **50th centile** | **75th centile** | **90th centile** | **95th centile** |
| 0.8 | 2.3 | 2.4 | 2.7 | 3.2 | 3.9 | 4.9 | 6.0 |
| 0.9 | 2.4 | 2.6 | 2.9 | 3.4 | 4.0 | 5.0 | 5.9 |
| 1.0 | 2.6 | 2.7 | 3.1 | 3.5 | 4.2 | 5.1 | 5.8 |
| 1.1 | 2.7 | 2.9 | 3.2 | 3.7 | 4.4 | 5.2 | 5.8 |
| 1.2 | 2.9 | 3.1 | 3.4 | 3.9 | 4.6 | 5.3 | 5.9 |
| 1.3 | 3.1 | 3.3 | 3.6 | 4.1 | 4.8 | 5.5 | 6.0 |
| 1.4 | 3.3 | 3.5 | 3.9 | 4.4 | 5.0 | 5.6 | 6.1 |
| 1.5 | 3.5 | 3.7 | 4.1 | 4.6 | 5.2 | 5.8 | 6.3 |
| 1.6 | 3.7 | 3.9 | 4.3 | 4.8 | 5.4 | 6.0 | 6.4 |
| 1.7 | 4.0 | 4.2 | 4.6 | 5.1 | 5.7 | 6.3 | 6.6 |
| 1.8 | 4.2 | 4.4 | 4.9 | 5.4 | 5.9 | 6.5 | 6.9 |
| 1.9 | 4.5 | 4.7 | 5.2 | 5.7 | 6.2 | 6.8 | 7.1 |
| 2.0 | 4.8 | 5.0 | 5.5 | 6.0 | 6.5 | 7.1 | 7.4 |
| 2.1 | 5.1 | 5.3 | 5.8 | 6.3 | 6.8 | 7.4 | 7.7 |
| 2.2 | 5.4 | 5.6 | 6.1 | 6.6 | 7.2 | 7.7 | 8.0 |

**Table S11.** Centiles of the myocardial thickness (presented in mm) of the LV midventricular inferolateral segment (segment 11) in boys by BSA. Of note, it was not possible to create a model for girls by BSA, however age related Table Sand graph for this segment is available in Supplement.

|  |  |  | **BOYS** |  |  |  |  |
| --- | --- | --- | --- | --- | --- | --- | --- |
| **BSA (m2)** | **5th centile** | **10th centile** | **25th centile** | **50th centile** | **75th centile** | **90th centile** | **95th centile** |
| 0.8 | 1.6 | 1.9 | 2.5 | 3.1 | 3.8 | 4.3 | 4.7 |
| 0.9 | 1.8 | 2.2 | 2.8 | 3.4 | 4.0 | 4.6 | 5.0 |
| 1.0 | 2.1 | 2.4 | 3.0 | 3.7 | 4.3 | 4.9 | 5.2 |
| 1.1 | 2.4 | 2.7 | 3.3 | 3.9 | 4.6 | 5.2 | 5.5 |
| 1.2 | 2.6 | 3.0 | 3.6 | 4.2 | 4.9 | 5.4 | 5.8 |
| 1.3 | 2.9 | 3.2 | 3.8 | 4.5 | 5.1 | 5.7 | 6.1 |
| 1.4 | 3.1 | 3.5 | 4.1 | 4.7 | 5.4 | 6.0 | 6.3 |
| 1.5 | 3.4 | 3.7 | 4.3 | 5.0 | 5.6 | 6.2 | 6.6 |
| 1.6 | 3.6 | 4.0 | 4.6 | 5.2 | 5.9 | 6.5 | 6.8 |
| 1.7 | 3.9 | 4.2 | 4.8 | 5.5 | 6.1 | 6.7 | 7.1 |
| 1.8 | 4.1 | 4.4 | 5.0 | 5.7 | 6.4 | 7.0 | 7.3 |
| 1.9 | 4.3 | 4.6 | 5.2 | 5.9 | 6.6 | 7.2 | 7.5 |
| 2.0 | 4.5 | 4.9 | 5.5 | 6.1 | 6.8 | 7.4 | 7.8 |
| 2.1 | 4.7 | 5.1 | 5.7 | 6.3 | 7.0 | 7.6 | 8.0 |
| 2.2 | 4.9 | 5.3 | 5.9 | 6.5 | 7.2 | 7.8 | 8.2 |

**Table S12.** Centiles of the myocardial thickness (presented in mm) of the LV midventricular anterolateral segment (segment 12) in boys by BSA. Of note, it was not possible to create a model for girls by BSA, however age related Table Sand graph for this segment is available here in Supplement.

|  |  |  | **BOYS** |  |  |  |  |
| --- | --- | --- | --- | --- | --- | --- | --- |
| **BSA (m2)** | **5th centile** | **10th centile** | **25th centile** | **50th centile** | **75th centile** | **90th centile** | **95th centile** |
| 0.8 | 1.7 | 2.0 | 2.5 | 3.1 | 3.6 | 4.1 | 4.4 |
| 0.9 | 2.0 | 2.3 | 2.8 | 3.3 | 3.9 | 4.4 | 4.7 |
| 1.0 | 2.2 | 2.5 | 3.0 | 3.5 | 4.1 | 4.6 | 4.9 |
| 1.1 | 2.4 | 2.7 | 3.2 | 3.8 | 4.3 | 4.8 | 5.2 |
| 1.2 | 2.6 | 2.9 | 3.4 | 4.0 | 4.6 | 5.1 | 5.4 |
| 1.3 | 2.8 | 3.1 | 3.6 | 4.2 | 4.8 | 5.3 | 5.6 |
| 1.4 | 3.0 | 3.3 | 3.8 | 4.4 | 5.0 | 5.6 | 5.9 |
| 1.5 | 3.2 | 3.5 | 4.0 | 4.6 | 5.2 | 5.8 | 6.1 |
| 1.6 | 3.4 | 3.7 | 4.2 | 4.8 | 5.5 | 6.0 | 6.3 |
| 1.7 | 3.6 | 3.9 | 4.4 | 5.0 | 5.7 | 6.2 | 6.5 |
| 1.8 | 3.7 | 4.1 | 4.6 | 5.2 | 5.9 | 6.4 | 6.8 |
| 1.9 | 3.9 | 4.2 | 4.8 | 5.4 | 6.1 | 6.6 | 7.0 |
| 2.0 | 4.1 | 4.4 | 5.0 | 5.6 | 6.3 | 6.8 | 7.2 |
| 2.1 | 4.2 | 4.6 | 5.2 | 5.8 | 6.4 | 7.0 | 7.4 |
| 2.2 | 4.4 | 4.7 | 5.3 | 6.0 | 6.6 | 7.2 | 7.6 |

**Table S13.** Centiles of the myocardial thickness (presented in mm) of the LV apical anterior segment (segment 13) in boys and girls by BSA.

|  |  |  | **BOYS** |  |  |  |  |
| --- | --- | --- | --- | --- | --- | --- | --- |
| **BSA (m2)** | **5th centile** | **10th centile** | **25th centile** | **50th centile** | **75th centile** | **90th centile** | **95th centile** |
| 0.8 | 1.9 | 2.1 | 2.3 | 2.5 | 2.7 | 3.0 | 3.1 |
| 0.9 | 2.0 | 2.1 | 2.3 | 2.6 | 2.8 | 3.0 | 3.2 |
| 1.0 | 2.1 | 2.2 | 2.4 | 2.7 | 2.9 | 3.1 | 3.3 |
| 1.1 | 2.1 | 2.3 | 2.5 | 2.8 | 3.0 | 3.3 | 3.4 |
| 1.2 | 2.2 | 2.3 | 2.6 | 2.9 | 3.1 | 3.4 | 3.5 |
| 1.3 | 2.3 | 2.4 | 2.7 | 3.0 | 3.3 | 3.5 | 3.7 |
| 1.4 | 2.4 | 2.6 | 2.8 | 3.1 | 3.4 | 3.7 | 3.9 |
| 1.5 | 2.5 | 2.7 | 3.0 | 3.3 | 3.6 | 3.9 | 4.0 |
| 1.6 | 2.7 | 2.8 | 3.1 | 3.5 | 3.8 | 4.1 | 4.3 |
| 1.7 | 2.8 | 3.0 | 3.3 | 3.7 | 4.0 | 4.3 | 4.5 |
| 1.8 | 3.0 | 3.2 | 3.5 | 3.9 | 4.2 | 4.5 | 4.7 |
| 1.9 | 3.2 | 3.4 | 3.7 | 4.1 | 4.4 | 4.8 | 5.0 |
| 2.0 | 3.3 | 3.5 | 3.9 | 4.3 | 4.7 | 5.0 | 5.2 |
| 2.1 | 3.5 | 3.7 | 4.1 | 4.5 | 4.9 | 5.2 | 5.5 |
| 2.2 | 3.7 | 3.9 | 4.3 | 4.7 | 5.1 | 5.5 | 5.7 |
|  |  |  | **GIRLS** |  |  |  |  |
| **BSA (m2)** | **5th centile** | **10th centile** | **25th centile** | **50th centile** | **75th centile** | **90th centile** | **95th centile** |
| 0.8 | 1.7 | 1.9 | 2.3 | 2.6 | 3.0 | 3.3 | 3.5 |
| 0.9 | 1.8 | 2.1 | 2.4 | 2.8 | 3.2 | 3.5 | 3.7 |
| 1.0 | 1.9 | 2.2 | 2.5 | 2.9 | 3.3 | 3.7 | 3.9 |
| 1.1 | 2.0 | 2.3 | 2.6 | 3.1 | 3.5 | 3.8 | 4.1 |
| 1.2 | 2.1 | 2.4 | 2.8 | 3.2 | 3.6 | 4.0 | 4.2 |
| 1.3 | 2.2 | 2.5 | 2.9 | 3.3 | 3.8 | 4.2 | 4.4 |
| 1.4 | 2.3 | 2.6 | 3.0 | 3.5 | 3.9 | 4.4 | 4.6 |
| 1.5 | 2.4 | 2.7 | 3.1 | 3.6 | 4.1 | 4.6 | 4.8 |
| 1.6 | 2.5 | 2.8 | 3.2 | 3.8 | 4.3 | 4.7 | 5.0 |
| 1.7 | 2.6 | 2.9 | 3.4 | 3.9 | 4.4 | 4.9 | 5.2 |
| 1.8 | 2.7 | 3.0 | 3.5 | 4.0 | 4.6 | 5.1 | 5.4 |
| 1.9 | 2.7 | 3.1 | 3.6 | 4.2 | 4.8 | 5.3 | 5.6 |
| 2.0 | 2.8 | 3.1 | 3.7 | 4.3 | 4.9 | 5.5 | 5.8 |
| 2.1 | 2.9 | 3.2 | 3.8 | 4.5 | 5.1 | 5.7 | 6.0 |
| 2.2 | 2.9 | 3.3 | 3.9 | 4.6 | 5.3 | 5.9 | 6.2 |

**Table S14.** Centiles of the myocardial thickness (presented in mm) of the LV apical septal segment (segment 14) in boys and girls by BSA.

|  |  |  | **BOYS** |  |  |  |  |
| --- | --- | --- | --- | --- | --- | --- | --- |
| **BSA (m2)** | **5th centile** | **10th centile** | **25th centile** | **50th centile** | **75th centile** | **90th centile** | **95th centile** |
| 0.8 | 1.6 | 1.8 | 2.3 | 2.7 | 3.2 | 3.6 | 3.8 |
| 0.9 | 1.8 | 2.0 | 2.4 | 2.9 | 3.4 | 3.8 | 4.0 |
| 1.0 | 1.9 | 2.2 | 2.6 | 3.1 | 3.6 | 4.0 | 4.3 |
| 1.1 | 2.1 | 2.3 | 2.8 | 3.3 | 3.8 | 4.2 | 4.5 |
| 1.2 | 2.2 | 2.5 | 3.0 | 3.5 | 4.0 | 4.4 | 4.7 |
| 1.3 | 2.4 | 2.7 | 3.1 | 3.7 | 4.2 | 4.6 | 4.9 |
| 1.4 | 2.6 | 2.8 | 3.3 | 3.8 | 4.4 | 4.8 | 5.1 |
| 1.5 | 2.7 | 3.0 | 3.5 | 4.0 | 4.6 | 5.0 | 5.3 |
| 1.6 | 2.9 | 3.2 | 3.7 | 4.2 | 4.8 | 5.2 | 5.5 |
| 1.7 | 3.0 | 3.3 | 3.8 | 4.4 | 5.0 | 5.5 | 5.8 |
| 1.8 | 3.2 | 3.5 | 4.0 | 4.6 | 5.2 | 5.7 | 6.0 |
| 1.9 | 3.4 | 3.7 | 4.2 | 4.8 | 5.4 | 5.9 | 6.2 |
| 2.0 | 3.5 | 3.8 | 4.4 | 5.0 | 5.6 | 6.1 | 6.4 |
| 2.1 | 3.7 | 4.0 | 4.5 | 5.1 | 5.8 | 6.3 | 6.6 |
| 2.2 | 3.8 | 4.2 | 4.7 | 5.3 | 6.0 | 6.5 | 6.8 |
|  |  |  | **GIRLS** |  |  |  |  |
| **BSA (m2)** | **5th centile** | **10th centile** | **25th centile** | **50th centile** | **75th centile** | **90th centile** | **95th centile** |
| 0.8 | 2.0 | 2.2 | 2.4 | 2.7 | 3.0 | 3.3 | 3.4 |
| 0.9 | 2.1 | 2.3 | 2.5 | 2.9 | 3.2 | 3.5 | 3.6 |
| 1.0 | 2.1 | 2.3 | 2.6 | 3.0 | 3.3 | 3.7 | 3.9 |
| 1.1 | 2.2 | 2.4 | 2.7 | 3.1 | 3.5 | 3.9 | 4.1 |
| 1.2 | 2.2 | 2.4 | 2.8 | 3.2 | 3.7 | 4.1 | 4.3 |
| 1.3 | 2.3 | 2.5 | 2.9 | 3.4 | 3.8 | 4.2 | 4.5 |
| 1.4 | 2.3 | 2.6 | 3.0 | 3.5 | 4.0 | 4.4 | 4.6 |
| 1.5 | 2.5 | 2.7 | 3.2 | 3.6 | 4.1 | 4.5 | 4.8 |
| 1.6 | 2.6 | 2.9 | 3.3 | 3.8 | 4.3 | 4.7 | 4.9 |
| 1.7 | 2.9 | 3.1 | 3.5 | 4.0 | 4.4 | 4.8 | 5.1 |
| 1.8 | 3.2 | 3.4 | 3.8 | 4.2 | 4.7 | 5.1 | 5.3 |
| 1.9 | 3.6 | 3.8 | 4.1 | 4.6 | 5.0 | 5.3 | 5.6 |
| 2.0 | 4.0 | 4.2 | 4.5 | 4.9 | 5.3 | 5.6 | 5.9 |
| 2.1 | 4.4 | 4.6 | 4.9 | 5.3 | 5.7 | 6.0 | 6.2 |
| 2.2 | 4.9 | 5.0 | 5.4 | 5.7 | 6.1 | 6.4 | 6.6 |

**Table S15.** Centiles of the myocardial thickness (presented in mm) of the LV apical inferior segment (segment 15) in boys and girls by BSA.

|  |  |  | **BOYS** |  |  |  |  |
| --- | --- | --- | --- | --- | --- | --- | --- |
| **BSA (m2)** | **5th centile** | **10th centile** | **25th centile** | **50th centile** | **75th centile** | **90th centile** | **95th centile** |
| 0.8 | 1.6 | 1.8 | 2.2 | 2.7 | 3.1 | 3.5 | 3.7 |
| 0.9 | 1.7 | 1.9 | 2.3 | 2.7 | 3.2 | 3.5 | 3.8 |
| 1.0 | 1.8 | 2.0 | 2.4 | 2.8 | 3.2 | 3.6 | 3.8 |
| 1.1 | 1.9 | 2.1 | 2.5 | 2.9 | 3.3 | 3.7 | 3.9 |
| 1.2 | 1.9 | 2.2 | 2.6 | 3.0 | 3.4 | 3.8 | 4.0 |
| 1.3 | 2.1 | 2.3 | 2.7 | 3.1 | 3.5 | 3.9 | 4.2 |
| 1.4 | 2.2 | 2.5 | 2.8 | 3.3 | 3.7 | 4.1 | 4.3 |
| 1.5 | 2.4 | 2.6 | 3.0 | 3.5 | 3.9 | 4.3 | 4.5 |
| 1.6 | 2.6 | 2.9 | 3.2 | 3.7 | 4.1 | 4.5 | 4.7 |
| 1.7 | 2.9 | 3.1 | 3.5 | 3.9 | 4.3 | 4.7 | 5.0 |
| 1.8 | 3.1 | 3.3 | 3.7 | 4.1 | 4.6 | 5.0 | 5.2 |
| 1.9 | 3.3 | 3.6 | 4.0 | 4.4 | 4.8 | 5.2 | 5.4 |
| 2.0 | 3.6 | 3.8 | 4.2 | 4.6 | 5.1 | 5.5 | 5.7 |
| 2.1 | 3.8 | 4.1 | 4.5 | 4.9 | 5.3 | 5.7 | 5.9 |
| 2.2 | 4.1 | 4.3 | 4.7 | 5.1 | 5.6 | 6.0 | 6.2 |
|  |  |  | **GIRLS** |  |  |  |  |
| **BSA (m2)** | **5th centile** | **10th centile** | **25th centile** | **50th centile** | **75th centile** | **90th centile** | **95th centile** |
| 0.8 | 2.0 | 2.1 | 2.3 | 2.6 | 2.9 | 3.1 | 3.3 |
| 0.9 | 2.0 | 2.1 | 2.4 | 2.7 | 3.0 | 3.3 | 3.5 |
| 1.0 | 2.0 | 2.2 | 2.5 | 2.9 | 3.2 | 3.5 | 3.7 |
| 1.1 | 2.1 | 2.3 | 2.7 | 3.1 | 3.4 | 3.8 | 4.0 |
| 1.2 | 2.2 | 2.4 | 2.8 | 3.2 | 3.7 | 4.1 | 4.3 |
| 1.3 | 2.3 | 2.5 | 2.9 | 3.4 | 3.9 | 4.3 | 4.5 |
| 1.4 | 2.4 | 2.7 | 3.1 | 3.6 | 4.1 | 4.5 | 4.8 |
| 1.5 | 2.7 | 2.9 | 3.4 | 3.8 | 4.3 | 4.7 | 5.0 |
| 1.6 | 3.0 | 3.2 | 3.6 | 4.1 | 4.5 | 5.0 | 5.2 |
| 1.7 | 3.3 | 3.5 | 3.9 | 4.3 | 4.8 | 5.2 | 5.4 |
| 1.8 | 3.5 | 3.7 | 4.1 | 4.5 | 4.9 | 5.3 | 5.5 |
| 1.9 | 3.6 | 3.8 | 4.1 | 4.5 | 4.9 | 5.3 | 5.5 |
| 2.0 | 3.5 | 3.7 | 4.1 | 4.5 | 4.8 | 5.2 | 5.4 |
| 2.1 | 3.4 | 3.6 | 3.9 | 4.3 | 4.7 | 5.0 | 5.2 |
| 2.2 | 3.3 | 3.4 | 3.8 | 4.1 | 4.4 | 4.8 | 4.9 |

**Table S16.** Centiles of the myocardial thickness (presented in mm) of the LV apical lateral segment (segment 16) in boys and girls by BSA.

|  |  |  | **BOYS** |  |  |  |  |
| --- | --- | --- | --- | --- | --- | --- | --- |
| **BSA (m2)** | **5th centile** | **10th centile** | **25th centile** | **50th centile** | **75th centile** | **90th centile** | **95th centile** |
| 0.8 | 2.2 | 2.3 | 2.5 | 2.7 | 3.0 | 3.1 | 3.3 |
| 0.9 | 2.2 | 2.3 | 2.6 | 2.8 | 3.1 | 3.4 | 3.5 |
| 1.0 | 2.1 | 2.3 | 2.6 | 3.0 | 3.3 | 3.6 | 3.8 |
| 1.1 | 2.1 | 2.3 | 2.7 | 3.1 | 3.5 | 3.9 | 4.1 |
| 1.2 | 2.0 | 2.3 | 2.8 | 3.3 | 3.8 | 4.2 | 4.5 |
| 1.3 | 2.1 | 2.4 | 2.9 | 3.4 | 4.0 | 4.5 | 4.8 |
| 1.4 | 2.2 | 2.5 | 3.0 | 3.6 | 4.2 | 4.8 | 5.1 |
| 1.5 | 2.3 | 2.6 | 3.2 | 3.8 | 4.4 | 5.0 | 5.3 |
| 1.6 | 2.5 | 2.8 | 3.4 | 4.0 | 4.6 | 5.2 | 5.5 |
| 1.7 | 2.7 | 3.1 | 3.6 | 4.2 | 4.8 | 5.3 | 5.6 |
| 1.8 | 2.9 | 3.2 | 3.7 | 4.3 | 4.8 | 5.3 | 5.6 |
| 1.9 | 3.1 | 3.3 | 3.8 | 4.3 | 4.8 | 5.3 | 5.6 |
| 2.0 | 3.1 | 3.4 | 3.8 | 4.3 | 4.8 | 5.2 | 5.4 |
| 2.1 | 3.2 | 3.4 | 3.8 | 4.2 | 4.6 | 5.0 | 5.2 |
| 2.2 | 3.2 | 3.4 | 3.7 | 4.0 | 4.4 | 4.7 | 4.9 |
|  |  |  | **GIRLS** |  |  |  |  |
| **BSA (m2)** | **5th centile** | **10th centile** | **25th centile** | **50th centile** | **75th centile** | **90th centile** | **95th centile** |
| 0.8 | 1.6 | 1.8 | 2.2 | 2.6 | 2.9 | 3.3 | 3.5 |
| 0.9 | 1.7 | 1.9 | 2.3 | 2.7 | 3.1 | 3.4 | 3.7 |
| 1.0 | 1.8 | 2.0 | 2.4 | 2.8 | 3.2 | 3.6 | 3.8 |
| 1.1 | 1.9 | 2.1 | 2.5 | 2.9 | 3.3 | 3.7 | 3.9 |
| 1.2 | 2.0 | 2.2 | 2.6 | 3.1 | 3.5 | 3.9 | 4.1 |
| 1.3 | 2.1 | 2.4 | 2.8 | 3.2 | 3.6 | 4.0 | 4.2 |
| 1.4 | 2.3 | 2.5 | 2.9 | 3.3 | 3.8 | 4.2 | 4.4 |
| 1.5 | 2.4 | 2.6 | 3.0 | 3.5 | 3.9 | 4.3 | 4.6 |
| 1.6 | 2.5 | 2.8 | 3.2 | 3.6 | 4.1 | 4.5 | 4.7 |
| 1.7 | 2.7 | 2.9 | 3.3 | 3.8 | 4.3 | 4.7 | 4.9 |
| 1.8 | 2.8 | 3.1 | 3.5 | 4.0 | 4.4 | 4.9 | 5.1 |
| 1.9 | 3.0 | 3.2 | 3.7 | 4.1 | 4.6 | 5.0 | 5.3 |
| 2.0 | 3.1 | 3.4 | 3.8 | 4.3 | 4.8 | 5.2 | 5.5 |
| 2.1 | 3.3 | 3.5 | 4.0 | 4.5 | 5.0 | 5.4 | 5.7 |
| 2.2 | 3.4 | 3.7 | 4.1 | 4.6 | 5.1 | 5.6 | 5.9 |

**Table S17.** Centiles of the myocardial thickness of the LV basal anterior segment (segment 1) in boys and girls age 6-18 years (presented in mm).

| **BOYS** | | | | | | | |
| --- | --- | --- | --- | --- | --- | --- | --- |
| **Age (years)** | **5^th^ centile** | **10^th^ centile** | **25^th^ centile** | **50^th^ centile** | **75^th^ centile** | **90^th^ centile** | **95^th^ centile** |
| 6 | 2.59 | 2.75 | 3.04 | 3.39 | 3.78 | 4.16 | 4.40 |
| 7 | 2.72 | 2.89 | 3.21 | 3.58 | 3.99 | 4.39 | 4.64 |
| 8 | 2.84 | 3.03 | 3.37 | 3.78 | 4.21 | 4.62 | 4.87 |
| 9 | 2.97 | 3.18 | 3.55 | 3.98 | 4.43 | 4.86 | 5.12 |
| 10 | 3.11 | 3.34 | 3.73 | 4.19 | 4.67 | 5.11 | 5.39 |
| 11 | 3.25 | 3.50 | 3.93 | 4.42 | 4.92 | 5.38 | 5.66 |
| 12 | 3.39 | 3.67 | 4.13 | 4.66 | 5.19 | 5.67 | 5.95 |
| 13 | 3.54 | 3.84 | 4.35 | 4.91 | 5.46 | 5.96 | 6.26 |
| 14 | 3.69 | 4.02 | 4.57 | 5.17 | 5.76 | 6.28 | 6.58 |
| 15 | 3.85 | 4.21 | 4.81 | 5.45 | 6.07 | 6.61 | 6.92 |
| 16 | 4.00 | 4.41 | 5.06 | 5.74 | 6.39 | 6.95 | 7.28 |
| 17 | 4.16 | 4.62 | 5.32 | 6.05 | 6.73 | 7.32 | 7.66 |
| 18 | 4.33 | 4.83 | 5.60 | 6.37 | 7.10 | 7.71 | 8.06 |
| **GIRLS** | | | | | | | |
| **Age (years)** | **5^th^ centile** | **10^th^ centile** | **25^th^ centile** | **50^th^ centile** | **75^th^ centile** | **90^th^ centile** | **95^th^ centile** |
| 6 | 2.15 | 2.43 | 2.88 | 3.38 | 3.87 | 4.31 | 4.57 |
| 7 | 2.33 | 2.59 | 3.02 | 3.51 | 4.02 | 4.48 | 4.75 |
| 8 | 2.51 | 2.75 | 3.17 | 3.65 | 4.17 | 4.65 | 4.95 |
| 9 | 2.69 | 2.92 | 3.32 | 3.80 | 4.33 | 4.84 | 5.16 |
| 10 | 2.87 | 3.09 | 3.48 | 3.96 | 4.50 | 5.04 | 5.38 |
| 11 | 3.06 | 3.27 | 3.65 | 4.13 | 4.68 | 5.24 | 5.62 |
| 12 | 3.24 | 3.44 | 3.82 | 4.29 | 4.86 | 5.45 | 5.85 |
| 13 | 3.42 | 3.61 | 3.97 | 4.45 | 5.02 | 5.64 | 6.08 |
| 14 | 3.57 | 3.76 | 4.12 | 4.59 | 5.16 | 5.81 | 6.27 |
| 15 | 3.71 | 3.89 | 4.23 | 4.70 | 5.28 | 5.94 | 6.43 |
| 16 | 3.82 | 4.00 | 4.33 | 4.78 | 5.36 | 6.04 | 6.55 |
| 17 | 3.93 | 4.09 | 4.41 | 4.86 | 5.43 | 6.13 | 6.66 |
| 18 | 4.03 | 4.19 | 4.50 | 4.93 | 5.50 | 6.20 | 6.77 |

**Table S18.** Centiles of the myocardial thickness of the LV basal anteroseptal segment (segment 2) in boys and girls age 6-18 years (presented in mm).

| **BOYS** | | | | | | | |
| --- | --- | --- | --- | --- | --- | --- | --- |
| **Age (years)** | **5th centile** | **10th centile** | **25th centile** | **50th centile** | **75th centile** | **90th centile** | **95th centile** |
| 6 | 2.26 | 2.61 | 3.19 | 3.84 | 4.48 | 5.06 | 5.41 |
| 7 | 2.52 | 2.87 | 3.44 | 4.08 | 4.72 | 5.29 | 5.64 |
| 8 | 2.78 | 3.12 | 3.69 | 4.32 | 4.96 | 5.53 | 5.87 |
| 9 | 3.04 | 3.38 | 3.94 | 4.57 | 5.19 | 5.76 | 6.09 |
| 10 | 3.30 | 3.63 | 4.19 | 4.81 | 5.43 | 5.99 | 6.32 |
| 11 | 3.56 | 3.89 | 4.44 | 5.06 | 5.67 | 6.22 | 6.55 |
| 12 | 3.82 | 4.14 | 4.69 | 5.30 | 5.91 | 6.45 | 6.78 |
| 13 | 4.07 | 4.40 | 4.94 | 5.54 | 6.14 | 6.69 | 7.01 |
| 14 | 4.33 | 4.65 | 5.19 | 5.79 | 6.38 | 6.92 | 7.24 |
| 15 | 4.59 | 4.91 | 5.44 | 6.03 | 6.62 | 7.15 | 7.47 |
| 16 | 4.85 | 5.16 | 5.69 | 6.27 | 6.86 | 7.38 | 7.70 |
| 17 | 5.11 | 5.42 | 5.94 | 6.52 | 7.10 | 7.62 | 7.93 |
| 18 | 5.36 | 5.67 | 6.19 | 6.76 | 7.33 | 7.85 | 8.16 |
| **GIRLS** | | | | | | | |
| **Age (years)** | **5th centile** | **10th centile** | **25th centile** | **50th centile** | **75th centile** | **90th centile** | **95th centile** |
| 6 | 2.76 | 3.06 | 3.57 | 4.14 | 4.71 | 5.23 | 5.53 |
| 7 | 2.89 | 3.20 | 3.72 | 4.29 | 4.86 | 5.38 | 5.69 |
| 8 | 3.03 | 3.34 | 3.86 | 4.43 | 5.01 | 5.53 | 5.84 |
| 9 | 3.16 | 3.47 | 4.00 | 4.58 | 5.16 | 5.68 | 5.99 |
| 10 | 3.30 | 3.61 | 4.14 | 4.72 | 5.30 | 5.83 | 6.14 |
| 11 | 3.43 | 3.75 | 4.28 | 4.86 | 5.45 | 5.98 | 6.29 |
| 12 | 3.57 | 3.89 | 4.42 | 5.01 | 5.60 | 6.13 | 6.45 |
| 13 | 3.70 | 4.02 | 4.56 | 5.15 | 5.74 | 6.28 | 6.60 |
| 14 | 3.84 | 4.16 | 4.70 | 5.30 | 5.89 | 6.43 | 6.75 |
| 15 | 3.98 | 4.30 | 4.84 | 5.44 | 6.04 | 6.58 | 6.90 |
| 16 | 4.11 | 4.44 | 4.98 | 5.58 | 6.19 | 6.73 | 7.05 |
| 17 | 4.25 | 4.57 | 5.12 | 5.73 | 6.33 | 6.88 | 7.21 |
| 18 | 4.38 | 4.71 | 5.26 | 5.87 | 6.48 | 7.03 | 7.36 |

**Table S19.** Centiles of the myocardial thickness of the LV basal inferoseptal segment (segment 3) in boys and girls age 6-18 years (presented in mm).

|  |  |  | **BOYS** |  |  |  |  |
| --- | --- | --- | --- | --- | --- | --- | --- |
| **Age (years)** | **5th centile** | **10th centile** | **25th centile** | **50th centile** | **75th centile** | **90th centile** | **95th centile** |
| 6 | 2.45 | 2.76 | 3.26 | 3.82 | 4.38 | 4.89 | 5.19 |
| 7 | 2.61 | 2.91 | 3.40 | 3.96 | 4.51 | 5.00 | 5.30 |
| 8 | 2.77 | 3.06 | 3.54 | 4.08 | 4.62 | 5.11 | 5.40 |
| 9 | 2.88 | 3.17 | 3.65 | 4.18 | 4.71 | 5.19 | 5.47 |
| 10 | 2.98 | 3.26 | 3.73 | 4.25 | 4.78 | 5.24 | 5.53 |
| 11 | 3.12 | 3.40 | 3.86 | 4.37 | 4.88 | 5.34 | 5.62 |
| 12 | 3.36 | 3.63 | 4.08 | 4.58 | 5.09 | 5.54 | 5.81 |
| 13 | 3.69 | 3.95 | 4.40 | 4.89 | 5.38 | 5.83 | 6.09 |
| 14 | 4.07 | 4.33 | 4.77 | 5.25 | 5.74 | 6.18 | 6.44 |
| 15 | 4.43 | 4.69 | 5.11 | 5.59 | 6.06 | 6.49 | 6.75 |
| 16 | 4.67 | 4.92 | 5.34 | 5.81 | 6.27 | 6.69 | 6.95 |
| 17 | 4.75 | 5.00 | 5.41 | 5.87 | 6.33 | 6.74 | 6.99 |
| 18 | 4.73 | 4.97 | 5.38 | 5.83 | 6.28 | 6.68 | 6.92 |
|  |  |  | **GIRLS** |  |  |  |  |
| **Age (years)** | **5th centile** | **10th centile** | **25th centile** | **50th centile** | **75th centile** | **90th centile** | **95th centile** |
| 6 | 2.33 | 2.64 | 3.14 | 3.71 | 4.27 | 4.77 | 5.08 |
| 7 | 2.44 | 2.74 | 3.25 | 3.82 | 4.39 | 4.91 | 5.21 |
| 8 | 2.54 | 2.85 | 3.37 | 3.94 | 4.52 | 5.04 | 5.35 |
| 9 | 2.64 | 2.95 | 3.48 | 4.06 | 4.64 | 5.17 | 5.48 |
| 10 | 2.74 | 3.06 | 3.59 | 4.18 | 4.77 | 5.30 | 5.62 |
| 11 | 2.84 | 3.16 | 3.70 | 4.30 | 4.90 | 5.43 | 5.76 |
| 12 | 2.94 | 3.27 | 3.81 | 4.42 | 5.02 | 5.57 | 5.89 |
| 13 | 3.05 | 3.37 | 3.93 | 4.54 | 5.15 | 5.70 | 6.03 |
| 14 | 3.15 | 3.48 | 4.04 | 4.66 | 5.27 | 5.83 | 6.16 |
| 15 | 3.25 | 3.58 | 4.15 | 4.77 | 5.40 | 5.96 | 6.30 |
| 16 | 3.35 | 3.69 | 4.26 | 4.89 | 5.53 | 6.10 | 6.44 |
| 17 | 3.45 | 3.79 | 4.37 | 5.01 | 5.65 | 6.23 | 6.58 |
| 18 | 3.55 | 3.90 | 4.48 | 5.13 | 5.78 | 6.36 | 6.71 |

**Table S20.** Centiles of the myocardial thickness of the LV basal inferior segment (segment 4) in boys and girls age 6-18 years (presented in mm).

|  |  |  | **BOYS** |  |  |  |  |
| --- | --- | --- | --- | --- | --- | --- | --- |
| **Age (years)** | **5th centile** | **10th centile** | **25th centile** | **50th centile** | **75th centile** | **90th centile** | **95th centile** |
| 6 | 2.39 | 2.64 | 3.05 | 3.51 | 3.96 | 4.38 | 4.62 |
| 7 | 2.54 | 2.80 | 3.22 | 3.69 | 4.16 | 4.58 | 4.84 |
| 8 | 2.70 | 2.96 | 3.39 | 3.87 | 4.36 | 4.79 | 5.05 |
| 9 | 2.85 | 3.12 | 3.56 | 4.06 | 4.55 | 4.99 | 5.26 |
| 10 | 3.01 | 3.28 | 3.73 | 4.24 | 4.75 | 5.20 | 5.47 |
| 11 | 3.17 | 3.45 | 3.92 | 4.44 | 4.96 | 5.42 | 5.70 |
| 12 | 3.37 | 3.66 | 4.14 | 4.67 | 5.20 | 5.68 | 5.96 |
| 13 | 3.60 | 3.89 | 4.38 | 4.93 | 5.47 | 5.96 | 6.26 |
| 14 | 3.86 | 4.16 | 4.66 | 5.22 | 5.78 | 6.28 | 6.58 |
| 15 | 4.13 | 4.44 | 4.95 | 5.52 | 6.10 | 6.61 | 6.92 |
| 16 | 4.39 | 4.71 | 5.24 | 5.82 | 6.41 | 6.94 | 7.25 |
| 17 | 4.64 | 4.97 | 5.51 | 6.11 | 6.71 | 7.25 | 7.58 |
| 18 | 4.88 | 5.22 | 5.77 | 6.39 | 7.00 | 7.56 | 7.89 |
|  |  |  | **GIRLS** |  |  |  |  |
| **Age (years)** | **5th centile** | **10th centile** | **25th centile** | **50th centile** | **75th centile** | **90th centile** | **95th centile** |
| 6 | 2.45 | 2.71 | 3.15 | 3.64 | 4.12 | 4.56 | 4.82 |
| 7 | 2.57 | 2.83 | 3.28 | 3.77 | 4.26 | 4.71 | 4.97 |
| 8 | 2.68 | 2.95 | 3.40 | 3.91 | 4.41 | 4.86 | 5.13 |
| 9 | 2.80 | 3.07 | 3.53 | 4.04 | 4.55 | 5.01 | 5.28 |
| 10 | 2.91 | 3.19 | 3.66 | 4.17 | 4.69 | 5.16 | 5.44 |
| 11 | 3.03 | 3.31 | 3.78 | 4.31 | 4.83 | 5.31 | 5.59 |
| 12 | 3.14 | 3.42 | 3.90 | 4.44 | 4.97 | 5.45 | 5.74 |
| 13 | 3.24 | 3.53 | 4.02 | 4.56 | 5.11 | 5.59 | 5.89 |
| 14 | 3.34 | 3.64 | 4.13 | 4.69 | 5.24 | 5.73 | 6.03 |
| 15 | 3.44 | 3.74 | 4.24 | 4.80 | 5.36 | 5.87 | 6.17 |
| 16 | 3.53 | 3.84 | 4.35 | 4.92 | 5.49 | 6.00 | 6.30 |
| 17 | 3.62 | 3.93 | 4.45 | 5.03 | 5.61 | 6.13 | 6.44 |
| 18 | 3.71 | 4.03 | 4.56 | 5.14 | 5.73 | 6.26 | 6.57 |

**Table S21.** Centiles of the myocardial thickness of the LV basal inferolateral segment (segment 5) in boys and girls age 6-18 years (presented in mm).

|  |  |  | **BOYS** |  |  |  |  |
| --- | --- | --- | --- | --- | --- | --- | --- |
| **Age (years)** | **5th centile** | **10th centile** | **25th centile** | **50th centile** | **75th centile** | **90th centile** | **95th centile** |
| 6 | 1.55 | 1.95 | 2.61 | 3.34 | 4.08 | 4.74 | 5.13 |
| 7 | 1.88 | 2.28 | 2.94 | 3.67 | 4.41 | 5.07 | 5.46 |
| 8 | 2.22 | 2.61 | 3.27 | 4.00 | 4.73 | 5.39 | 5.78 |
| 9 | 2.54 | 2.94 | 3.59 | 4.32 | 5.05 | 5.71 | 6.10 |
| 10 | 2.85 | 3.24 | 3.90 | 4.63 | 5.36 | 6.01 | 6.40 |
| 11 | 3.15 | 3.54 | 4.20 | 4.92 | 5.65 | 6.30 | 6.69 |
| 12 | 3.46 | 3.85 | 4.50 | 5.23 | 5.95 | 6.60 | 6.99 |
| 13 | 3.78 | 4.16 | 4.82 | 5.54 | 6.26 | 6.91 | 7.30 |
| 14 | 4.09 | 4.48 | 5.12 | 5.85 | 6.57 | 7.22 | 7.60 |
| 15 | 4.36 | 4.75 | 5.40 | 6.12 | 6.84 | 7.48 | 7.87 |
| 16 | 4.58 | 4.97 | 5.62 | 6.33 | 7.05 | 7.70 | 8.08 |
| 17 | 4.75 | 5.14 | 5.78 | 6.50 | 7.22 | 7.86 | 8.25 |
| 18 | 4.90 | 5.28 | 5.93 | 6.64 | 7.35 | 8.00 | 8.38 |
|  |  |  | **GIRLS** |  |  |  |  |
| **Age (years)** | **5th centile** | **10th centile** | **25th centile** | **50th centile** | **75th centile** | **90th centile** | **95th centile** |
| 6 | 1.58 | 1.94 | 2.54 | 3.21 | 3.88 | 4.48 | 4.84 |
| 7 | 1.82 | 2.18 | 2.78 | 3.45 | 4.12 | 4.72 | 5.08 |
| 8 | 2.05 | 2.41 | 3.02 | 3.69 | 4.36 | 4.96 | 5.33 |
| 9 | 2.29 | 2.65 | 3.26 | 3.93 | 4.60 | 5.21 | 5.57 |
| 10 | 2.53 | 2.89 | 3.50 | 4.17 | 4.85 | 5.45 | 5.82 |
| 11 | 2.77 | 3.13 | 3.74 | 4.42 | 5.09 | 5.70 | 6.07 |
| 12 | 3.01 | 3.37 | 3.98 | 4.66 | 5.34 | 5.95 | 6.31 |
| 13 | 3.23 | 3.60 | 4.21 | 4.89 | 5.57 | 6.18 | 6.54 |
| 14 | 3.40 | 3.77 | 4.38 | 5.06 | 5.74 | 6.35 | 6.72 |
| 15 | 3.50 | 3.87 | 4.48 | 5.16 | 5.85 | 6.46 | 6.83 |
| 16 | 3.52 | 3.89 | 4.50 | 5.19 | 5.87 | 6.48 | 6.85 |
| 17 | 3.47 | 3.84 | 4.45 | 5.14 | 5.82 | 6.44 | 6.81 |
| 18 | 3.38 | 3.75 | 4.37 | 5.05 | 5.74 | 6.36 | 6.73 |

**Table S22.** Centiles of the myocardial thickness of the LV basal anterolateral segment (segment 6) in boys and girls age 6-18 years (presented in mm).

|  |  |  | **BOYS** |  |  |  |  |
| --- | --- | --- | --- | --- | --- | --- | --- |
| **Age (years)** | **5th centile** | **10th centile** | **25th centile** | **50th centile** | **75th centile** | **90th centile** | **95th centile** |
| 6 | 1.89 | 2.18 | 2.67 | 3.21 | 3.75 | 4.23 | 4.53 |
| 7 | 2.15 | 2.44 | 2.94 | 3.48 | 4.03 | 4.52 | 4.82 |
| 8 | 2.41 | 2.71 | 3.20 | 3.76 | 4.31 | 4.81 | 5.11 |
| 9 | 2.66 | 2.96 | 3.47 | 4.03 | 4.59 | 5.09 | 5.39 |
| 10 | 2.90 | 3.21 | 3.72 | 4.29 | 4.85 | 5.36 | 5.67 |
| 11 | 3.15 | 3.46 | 3.97 | 4.55 | 5.12 | 5.64 | 5.95 |
| 12 | 3.40 | 3.71 | 4.24 | 4.82 | 5.40 | 5.92 | 6.23 |
| 13 | 3.66 | 3.98 | 4.51 | 5.10 | 5.68 | 6.21 | 6.53 |
| 14 | 3.92 | 4.24 | 4.77 | 5.37 | 5.96 | 6.50 | 6.82 |
| 15 | 4.14 | 4.46 | 5.00 | 5.61 | 6.21 | 6.75 | 7.07 |
| 16 | 4.31 | 4.64 | 5.19 | 5.80 | 6.41 | 6.96 | 7.28 |
| 17 | 4.45 | 4.79 | 5.34 | 5.96 | 6.57 | 7.13 | 7.46 |
| 18 | 4.58 | 4.92 | 5.48 | 6.10 | 6.72 | 7.28 | 7.62 |
|  |  |  | **GIRLS** |  |  |  |  |
| **Age (years)** | **5th centile** | **10th centile** | **25th centile** | **50th centile** | **75th centile** | **90th centile** | **95th centile** |
| 6 | 2.79 | 2.96 | 3.24 | 3.56 | 3.87 | 4.15 | 4.32 |
| 7 | 2.80 | 2.99 | 3.31 | 3.67 | 4.03 | 4.35 | 4.55 |
| 8 | 2.80 | 3.02 | 3.38 | 3.79 | 4.19 | 4.56 | 4.78 |
| 9 | 2.80 | 3.05 | 3.45 | 3.91 | 4.36 | 4.77 | 5.01 |
| 10 | 2.81 | 3.08 | 3.53 | 4.03 | 4.52 | 4.97 | 5.24 |
| 11 | 2.84 | 3.13 | 3.61 | 4.14 | 4.68 | 5.16 | 5.45 |
| 12 | 2.89 | 3.19 | 3.70 | 4.26 | 4.82 | 5.32 | 5.62 |
| 13 | 2.98 | 3.28 | 3.80 | 4.37 | 4.94 | 5.46 | 5.76 |
| 14 | 3.08 | 3.39 | 3.91 | 4.48 | 5.05 | 5.57 | 5.88 |
| 15 | 3.20 | 3.51 | 4.02 | 4.59 | 5.16 | 5.67 | 5.98 |
| 16 | 3.32 | 3.62 | 4.13 | 4.69 | 5.25 | 5.76 | 6.06 |
| 17 | 3.44 | 3.74 | 4.24 | 4.80 | 5.35 | 5.85 | 6.15 |
| 18 | 3.57 | 3.86 | 4.35 | 4.90 | 5.44 | 5.93 | 6.22 |

**Table S23.** Centiles of the myocardial thickness of the LV midventricular anterior segment (segment 7) in boys and girls age 6-18 years (presented in mm).

|  |  |  | **BOYS** |  |  |  |  |
| --- | --- | --- | --- | --- | --- | --- | --- |
| **Age (years)** | **5th centile** | **10th centile** | **25th centile** | **50th centile** | **75th centile** | **90th centile** | **95th centile** |
| 6 | 1.96 | 2.19 | 2.57 | 3.00 | 3.43 | 3.81 | 4.04 |
| 7 | 2.08 | 2.33 | 2.73 | 3.19 | 3.64 | 4.05 | 4.29 |
| 8 | 2.20 | 2.46 | 2.89 | 3.37 | 3.85 | 4.28 | 4.54 |
| 9 | 2.32 | 2.60 | 3.05 | 3.56 | 4.06 | 4.52 | 4.79 |
| 10 | 2.44 | 2.73 | 3.21 | 3.74 | 4.28 | 4.76 | 5.05 |
| 11 | 2.56 | 2.86 | 3.37 | 3.93 | 4.49 | 5.00 | 5.30 |
| 12 | 2.68 | 3.00 | 3.53 | 4.12 | 4.71 | 5.24 | 5.55 |
| 13 | 2.81 | 3.14 | 3.69 | 4.30 | 4.92 | 5.47 | 5.80 |
| 14 | 2.94 | 3.28 | 3.85 | 4.49 | 5.12 | 5.69 | 6.03 |
| 15 | 3.08 | 3.44 | 4.02 | 4.67 | 5.33 | 5.91 | 6.26 |
| 16 | 3.24 | 3.60 | 4.20 | 4.86 | 5.52 | 6.12 | 6.48 |
| 17 | 3.41 | 3.77 | 4.37 | 5.05 | 5.72 | 6.32 | 6.69 |
| 18 | 3.58 | 3.94 | 4.55 | 5.23 | 5.91 | 6.52 | 6.88 |
|  |  |  | **GIRLS** |  |  |  |  |
| **Age (years)** | **5th centile** | **10th centile** | **25th centile** | **50th centile** | **75th centile** | **90th centile** | **95th centile** |
| 6 | 2.34 | 2.40 | 2.53 | 2.73 | 3.06 | 3.53 | 3.95 |
| 7 | 2.40 | 2.46 | 2.61 | 2.85 | 3.22 | 3.79 | 4.30 |
| 8 | 2.45 | 2.53 | 2.70 | 2.97 | 3.42 | 4.10 | 4.74 |
| 9 | 2.51 | 2.60 | 2.80 | 3.12 | 3.64 | 4.48 | 5.29 |
| 10 | 2.57 | 2.68 | 2.91 | 3.28 | 3.90 | 4.93 | 5.98 |
| 11 | 2.62 | 2.75 | 3.02 | 3.45 | 4.15 | 5.34 | 6.60 |
| 12 | 2.67 | 2.82 | 3.13 | 3.60 | 4.32 | 5.41 | 6.48 |
| 13 | 2.71 | 2.88 | 3.23 | 3.72 | 4.38 | 5.20 | 5.85 |
| 14 | 2.72 | 2.93 | 3.33 | 3.84 | 4.45 | 5.10 | 5.54 |
| 15 | 2.72 | 2.98 | 3.43 | 3.97 | 4.54 | 5.08 | 5.41 |
| 16 | 2.69 | 3.02 | 3.54 | 4.10 | 4.63 | 5.10 | 5.37 |
| 17 | 2.64 | 3.06 | 3.66 | 4.23 | 4.74 | 5.15 | 5.38 |
| 18 | 2.71 | 3.17 | 3.81 | 4.39 | 4.86 | 5.23 | 5.43 |

**Table S24.** Centiles of the myocardial thickness of the LV midventricular anteroseptal segment (segment 8) in boys and girls age 6-18 years (presented in mm).

|  |  |  | **BOYS** |  |  |  |  |
| --- | --- | --- | --- | --- | --- | --- | --- |
| **Age (years)** | **5th centile** | **10th centile** | **25th centile** | **50th centile** | **75th centile** | **90th centile** | **95th centile** |
| 6 | 2.68 | 2.96 | 3.44 | 3.97 | 4.51 | 4.99 | 5.27 |
| 7 | 2.85 | 3.15 | 3.64 | 4.19 | 4.74 | 5.23 | 5.52 |
| 8 | 3.03 | 3.33 | 3.84 | 4.40 | 4.97 | 5.47 | 5.78 |
| 9 | 3.20 | 3.52 | 4.04 | 4.62 | 5.20 | 5.72 | 6.03 |
| 10 | 3.38 | 3.70 | 4.24 | 4.83 | 5.43 | 5.96 | 6.28 |
| 11 | 3.55 | 3.88 | 4.43 | 5.05 | 5.66 | 6.21 | 6.54 |
| 12 | 3.72 | 4.06 | 4.63 | 5.26 | 5.89 | 6.46 | 6.80 |
| 13 | 3.89 | 4.24 | 4.83 | 5.47 | 6.12 | 6.70 | 7.05 |
| 14 | 4.06 | 4.42 | 5.02 | 5.69 | 6.35 | 6.95 | 7.31 |
| 15 | 4.23 | 4.60 | 5.22 | 5.90 | 6.59 | 7.20 | 7.57 |
| 16 | 4.40 | 4.78 | 5.41 | 6.12 | 6.82 | 7.45 | 7.83 |
| 17 | 4.56 | 4.95 | 5.60 | 6.33 | 7.05 | 7.71 | 8.10 |
| 18 | 4.73 | 5.13 | 5.80 | 6.54 | 7.29 | 7.96 | 8.36 |
|  |  |  | **GIRLS** |  |  |  |  |
| **Age (years)** | **5th centile** | **10th centile** | **25th centile** | **50th centile** | **75th centile** | **90th centile** | **95th centile** |
| 6 | 3.53 | 3.64 | 3.85 | 4.12 | 4.44 | 4.78 | 5.01 |
| 7 | 3.56 | 3.69 | 3.92 | 4.21 | 4.56 | 4.94 | 5.20 |
| 8 | 3.60 | 3.73 | 3.98 | 4.31 | 4.69 | 5.11 | 5.39 |
| 9 | 3.62 | 3.77 | 4.05 | 4.40 | 4.83 | 5.28 | 5.60 |
| 10 | 3.65 | 3.81 | 4.11 | 4.50 | 4.97 | 5.47 | 5.82 |
| 11 | 3.66 | 3.84 | 4.17 | 4.60 | 5.11 | 5.66 | 6.04 |
| 12 | 3.67 | 3.87 | 4.23 | 4.70 | 5.26 | 5.87 | 6.29 |
| 13 | 3.68 | 3.89 | 4.29 | 4.81 | 5.42 | 6.09 | 6.54 |
| 14 | 3.67 | 3.91 | 4.34 | 4.91 | 5.59 | 6.31 | 6.81 |
| 15 | 3.66 | 3.91 | 4.40 | 5.02 | 5.76 | 6.55 | 7.09 |
| 16 | 3.63 | 3.91 | 4.44 | 5.13 | 5.95 | 6.80 | 7.38 |
| 17 | 3.59 | 3.90 | 4.49 | 5.25 | 6.14 | 7.07 | 7.69 |
| 18 | 3.53 | 3.88 | 4.53 | 5.36 | 6.34 | 7.34 | 8.01 |

**Table S25.** Centiles of the myocardial thickness of the LV midventricular inferoseptal segment (segment 9) girls age 6-18 years (presented in mm). Of note, it was not possible to create a model for boys by BSA, however age related Table Sand graph for this segment is available here in the Supplement.

|  |  |  | **GIRLS** |  |  |  |  |
| --- | --- | --- | --- | --- | --- | --- | --- |
| **Age (years)** | **5th centile** | **10th centile** | **25th centile** | **50th centile** | **75th centile** | **90th centile** | **95th centile** |
| 6 | 3.29 | 3.61 | 4.04 | 4.39 | 4.66 | 4.86 | 4.97 |
| 7 | 3.30 | 3.65 | 4.11 | 4.49 | 4.79 | 5.02 | 5.14 |
| 8 | 3.32 | 3.69 | 4.18 | 4.60 | 4.93 | 5.19 | 5.33 |
| 9 | 3.34 | 3.73 | 4.25 | 4.71 | 5.08 | 5.37 | 5.53 |
| 10 | 3.38 | 3.78 | 4.32 | 4.82 | 5.23 | 5.56 | 5.75 |
| 11 | 3.45 | 3.84 | 4.40 | 4.93 | 5.40 | 5.78 | 5.99 |
| 12 | 3.56 | 3.92 | 4.48 | 5.05 | 5.57 | 6.01 | 6.26 |
| 13 | 3.68 | 4.02 | 4.57 | 5.17 | 5.76 | 6.27 | 6.57 |
| 14 | 3.80 | 4.12 | 4.66 | 5.30 | 5.96 | 6.57 | 6.94 |
| 15 | 3.92 | 4.22 | 4.76 | 5.43 | 6.17 | 6.92 | 7.40 |
| 16 | 4.05 | 4.33 | 4.86 | 5.56 | 6.41 | 7.34 | 7.98 |
| 17 | 4.17 | 4.44 | 4.96 | 5.70 | 6.67 | 7.88 | 8.82 |
| 18 | 4.30 | 4.55 | 5.06 | 5.83 | 6.97 | 8.61 | 10.16 |

**Table S26.** Centiles of the myocardial thickness of the LV midventricular inferior segment (segment 10) in boys and girls age 6-18 years (presented in mm).

|  |  |  | **BOYS** |  |  |  |  |
| --- | --- | --- | --- | --- | --- | --- | --- |
| **Age (years)** | **5th centile** | **10th centile** | **25th centile** | **50th centile** | **75th centile** | **90th centile** | **95th centile** |
| 6 | 1.63 | 1.96 | 2.51 | 3.12 | 3.73 | 4.28 | 4.61 |
| 7 | 1.87 | 2.20 | 2.75 | 3.36 | 3.97 | 4.52 | 4.85 |
| 8 | 2.10 | 2.43 | 2.98 | 3.60 | 4.21 | 4.76 | 5.09 |
| 9 | 2.34 | 2.67 | 3.22 | 3.83 | 4.45 | 5.00 | 5.33 |
| 10 | 2.58 | 2.91 | 3.46 | 4.07 | 4.68 | 5.23 | 5.56 |
| 11 | 2.81 | 3.14 | 3.69 | 4.31 | 4.92 | 5.47 | 5.80 |
| 12 | 3.05 | 3.38 | 3.93 | 4.54 | 5.16 | 5.71 | 6.04 |
| 13 | 3.28 | 3.61 | 4.17 | 4.78 | 5.39 | 5.95 | 6.28 |
| 14 | 3.52 | 3.85 | 4.40 | 5.02 | 5.63 | 6.18 | 6.52 |
| 15 | 3.75 | 4.08 | 4.64 | 5.25 | 5.87 | 6.42 | 6.75 |
| 16 | 3.99 | 4.32 | 4.87 | 5.49 | 6.11 | 6.66 | 6.99 |
| 17 | 4.22 | 4.56 | 5.11 | 5.73 | 6.34 | 6.90 | 7.23 |
| 18 | 4.46 | 4.79 | 5.35 | 5.96 | 6.58 | 7.13 | 7.47 |
|  |  |  | **GIRLS** |  |  |  |  |
| **Age (years)** | **5th centile** | **10th centile** | **25th centile** | **50th centile** | **75th centile** | **90th centile** | **95th centile** |
| 6 | 3.04 | 3.13 | 3.29 | 3.51 | 3.79 | 4.11 | 4.35 |
| 7 | 3.08 | 3.18 | 3.36 | 3.60 | 3.91 | 4.27 | 4.54 |
| 8 | 3.11 | 3.22 | 3.43 | 3.70 | 4.04 | 4.44 | 4.73 |
| 9 | 3.14 | 3.26 | 3.49 | 3.80 | 4.18 | 4.61 | 4.93 |
| 10 | 3.16 | 3.30 | 3.56 | 3.90 | 4.33 | 4.79 | 5.13 |
| 11 | 3.16 | 3.32 | 3.62 | 4.01 | 4.48 | 4.98 | 5.33 |
| 12 | 3.15 | 3.34 | 3.68 | 4.12 | 4.63 | 5.18 | 5.55 |
| 13 | 3.12 | 3.34 | 3.73 | 4.23 | 4.80 | 5.38 | 5.76 |
| 14 | 3.06 | 3.32 | 3.78 | 4.34 | 4.97 | 5.58 | 5.98 |
| 15 | 2.97 | 3.28 | 3.82 | 4.46 | 5.14 | 5.79 | 6.19 |
| 16 | 2.81 | 3.19 | 3.85 | 4.58 | 5.33 | 6.00 | 6.41 |
| 17 | 2.56 | 3.06 | 3.86 | 4.71 | 5.52 | 6.22 | 6.64 |
| 18 | 2.31 | 2.93 | 3.89 | 4.85 | 5.72 | 6.45 | 6.86 |

**Table S27.** Centiles of the myocardial thickness of the LV midventricular inferolateral segment (segment 11) in boys and girls age 6-18 years (presented in mm).

|  |  |  | **BOYS** |  |  |  |  |
| --- | --- | --- | --- | --- | --- | --- | --- |
| **Age (years)** | **5th centile** | **10th centile** | **25th centile** | **50th centile** | **75th centile** | **90th centile** | **95th centile** |
| 6 | 1.46 | 1.81 | 2.39 | 3.04 | 3.69 | 4.27 | 4.62 |
| 7 | 1.74 | 2.09 | 2.68 | 3.33 | 3.98 | 4.57 | 4.92 |
| 8 | 2.01 | 2.37 | 2.96 | 3.62 | 4.28 | 4.87 | 5.23 |
| 9 | 2.28 | 2.64 | 3.24 | 3.91 | 4.57 | 5.17 | 5.53 |
| 10 | 2.55 | 2.91 | 3.52 | 4.19 | 4.86 | 5.47 | 5.83 |
| 11 | 2.82 | 3.18 | 3.80 | 4.47 | 5.15 | 5.76 | 6.13 |
| 12 | 3.09 | 3.45 | 4.07 | 4.76 | 5.44 | 6.06 | 6.43 |
| 13 | 3.35 | 3.72 | 4.34 | 5.03 | 5.72 | 6.35 | 6.72 |
| 14 | 3.59 | 3.97 | 4.60 | 5.29 | 5.99 | 6.62 | 7.00 |
| 15 | 3.81 | 4.19 | 4.82 | 5.53 | 6.23 | 6.86 | 7.24 |
| 16 | 3.98 | 4.37 | 5.01 | 5.72 | 6.43 | 7.07 | 7.45 |
| 17 | 4.13 | 4.51 | 5.16 | 5.88 | 6.60 | 7.24 | 7.63 |
| 18 | 4.25 | 4.64 | 5.30 | 6.02 | 6.74 | 7.40 | 7.79 |
|  |  |  | **GIRLS** |  |  |  |  |
| **Age (years)** | **5th centile** | **10th centile** | **25th centile** | **50th centile** | **75th centile** | **90th centile** | **95th centile** |
| 6 | 2.58 | 2.78 | 3.12 | 3.49 | 3.87 | 4.20 | 4.40 |
| 7 | 2.65 | 2.87 | 3.23 | 3.63 | 4.05 | 4.42 | 4.65 |
| 8 | 2.72 | 2.95 | 3.34 | 3.78 | 4.23 | 4.65 | 4.90 |
| 9 | 2.79 | 3.03 | 3.44 | 3.92 | 4.43 | 4.89 | 5.18 |
| 10 | 2.86 | 3.11 | 3.55 | 4.07 | 4.62 | 5.15 | 5.47 |
| 11 | 2.93 | 3.19 | 3.66 | 4.22 | 4.83 | 5.42 | 5.79 |
| 12 | 3.00 | 3.27 | 3.77 | 4.38 | 5.05 | 5.71 | 6.13 |
| 13 | 3.06 | 3.35 | 3.88 | 4.53 | 5.27 | 6.02 | 6.50 |
| 14 | 3.10 | 3.39 | 3.94 | 4.64 | 5.45 | 6.29 | 6.84 |
| 15 | 3.07 | 3.37 | 3.92 | 4.65 | 5.52 | 6.44 | 7.07 |
| 16 | 2.97 | 3.25 | 3.81 | 4.55 | 5.46 | 6.45 | 7.15 |
| 17 | 2.81 | 3.09 | 3.62 | 4.36 | 5.30 | 6.36 | 7.12 |
| 18 | 2.64 | 2.90 | 3.41 | 4.14 | 5.09 | 6.22 | 7.06 |

**Table S28.** Centiles of the myocardial thickness of the LV midventricular anterolateral segment (segment 12) in boys and girls age 6-18 years (presented in mm).

|  |  |  | **BOYS** |  |  |  |  |
| --- | --- | --- | --- | --- | --- | --- | --- |
| **Age (years)** | **5th centile** | **10th centile** | **25th centile** | **50th centile** | **75th centile** | **90th centile** | **95th centile** |
| 6 | 1.94 | 2.19 | 2.61 | 3.08 | 3.54 | 3.96 | 4.22 |
| 7 | 2.10 | 2.37 | 2.81 | 3.31 | 3.80 | 4.25 | 4.51 |
| 8 | 2.26 | 2.54 | 3.01 | 3.54 | 4.06 | 4.53 | 4.81 |
| 9 | 2.42 | 2.72 | 3.22 | 3.77 | 4.32 | 4.82 | 5.12 |
| 10 | 2.58 | 2.89 | 3.42 | 4.00 | 4.58 | 5.11 | 5.42 |
| 11 | 2.74 | 3.07 | 3.62 | 4.23 | 4.85 | 5.40 | 5.73 |
| 12 | 2.92 | 3.26 | 3.83 | 4.47 | 5.10 | 5.67 | 6.01 |
| 13 | 3.10 | 3.45 | 4.04 | 4.69 | 5.34 | 5.93 | 6.28 |
| 14 | 3.28 | 3.64 | 4.23 | 4.90 | 5.56 | 6.16 | 6.51 |
| 15 | 3.44 | 3.80 | 4.41 | 5.08 | 5.75 | 6.35 | 6.71 |
| 16 | 3.58 | 3.94 | 4.55 | 5.23 | 5.91 | 6.51 | 6.88 |
| 17 | 3.70 | 4.07 | 4.68 | 5.36 | 6.04 | 6.65 | 7.02 |
| 18 | 3.81 | 4.18 | 4.80 | 5.48 | 6.17 | 6.79 | 7.15 |
|  |  |  | **GIRLS** |  |  |  |  |
| **Age (years)** | **5th centile** | **10th centile** | **25th centile** | **50th centile** | **75th centile** | **90th centile** | **95th centile** |
| 6 | 2.27 | 2.41 | 2.68 | 3.00 | 3.37 | 3.75 | 3.99 |
| 7 | 2.34 | 2.50 | 2.78 | 3.13 | 3.54 | 3.95 | 4.22 |
| 8 | 2.42 | 2.58 | 2.89 | 3.27 | 3.71 | 4.16 | 4.46 |
| 9 | 2.51 | 2.69 | 3.02 | 3.44 | 3.92 | 4.42 | 4.75 |
| 10 | 2.61 | 2.81 | 3.16 | 3.62 | 4.15 | 4.71 | 5.08 |
| 11 | 2.72 | 2.93 | 3.32 | 3.82 | 4.41 | 5.02 | 5.43 |
| 12 | 2.82 | 3.05 | 3.47 | 4.02 | 4.66 | 5.34 | 5.80 |
| 13 | 2.90 | 3.14 | 3.59 | 4.18 | 4.88 | 5.63 | 6.14 |
| 14 | 2.93 | 3.18 | 3.66 | 4.28 | 5.04 | 5.84 | 6.40 |
| 15 | 2.89 | 3.15 | 3.64 | 4.29 | 5.08 | 5.93 | 6.52 |
| 16 | 2.80 | 3.06 | 3.55 | 4.21 | 5.02 | 5.91 | 6.52 |
| 17 | 2.68 | 2.94 | 3.43 | 4.10 | 4.92 | 5.83 | 6.47 |
| 18 | 2.56 | 2.81 | 3.30 | 3.97 | 4.81 | 5.74 | 6.40 |

**Table S29.** Centiles of the myocardial thickness of the LV apical anterior segment (segment 13) in boys and girls age 6-18 years (presented in mm).

|  |  |  | **BOYS** |  |  |  |  |
| --- | --- | --- | --- | --- | --- | --- | --- |
| **Age (years)** | **5th centile** | **10th centile** | **25th centile** | **50th centile** | **75th centile** | **90th centile** | **95th centile** |
| 6 | 1.57 | 1.76 | 2.10 | 2.46 | 2.83 | 3.16 | 3.36 |
| 7 | 1.69 | 1.90 | 2.24 | 2.62 | 3.01 | 3.35 | 3.56 |
| 8 | 1.82 | 2.03 | 2.39 | 2.79 | 3.19 | 3.54 | 3.76 |
| 9 | 1.94 | 2.16 | 2.54 | 2.95 | 3.36 | 3.74 | 3.96 |
| 10 | 2.06 | 2.30 | 2.68 | 3.11 | 3.54 | 3.93 | 4.16 |
| 11 | 2.18 | 2.43 | 2.83 | 3.28 | 3.73 | 4.13 | 4.37 |
| 12 | 2.30 | 2.55 | 2.97 | 3.44 | 3.91 | 4.33 | 4.58 |
| 13 | 2.42 | 2.68 | 3.12 | 3.60 | 4.09 | 4.53 | 4.79 |
| 14 | 2.54 | 2.81 | 3.26 | 3.77 | 4.27 | 4.73 | 5.00 |
| 15 | 2.65 | 2.93 | 3.40 | 3.93 | 4.45 | 4.93 | 5.21 |
| 16 | 2.76 | 3.05 | 3.55 | 4.09 | 4.64 | 5.13 | 5.43 |
| 17 | 2.87 | 3.18 | 3.69 | 4.26 | 4.82 | 5.34 | 5.64 |
| 18 | 2.98 | 3.29 | 3.83 | 4.42 | 5.01 | 5.54 | 5.86 |
|  |  |  | **GIRLS** |  |  |  |  |
| **Age (years)** | **5th centile** | **10th centile** | **25th centile** | **50th centile** | **75th centile** | **90th centile** | **95th centile** |
| 6 | 2.08 | 2.20 | 2.38 | 2.59 | 2.80 | 2.99 | 3.10 |
| 7 | 2.14 | 2.26 | 2.46 | 2.69 | 2.91 | 3.11 | 3.24 |
| 8 | 2.20 | 2.33 | 2.54 | 2.79 | 3.03 | 3.25 | 3.38 |
| 9 | 2.25 | 2.39 | 2.62 | 2.88 | 3.14 | 3.38 | 3.52 |
| 10 | 2.29 | 2.44 | 2.70 | 2.98 | 3.26 | 3.52 | 3.67 |
| 11 | 2.34 | 2.50 | 2.77 | 3.08 | 3.38 | 3.66 | 3.82 |
| 12 | 2.37 | 2.55 | 2.85 | 3.18 | 3.50 | 3.80 | 3.98 |
| 13 | 2.41 | 2.60 | 2.92 | 3.27 | 3.63 | 3.95 | 4.14 |
| 14 | 2.44 | 2.64 | 2.99 | 3.37 | 3.75 | 4.10 | 4.30 |
| 15 | 2.46 | 2.68 | 3.05 | 3.47 | 3.88 | 4.25 | 4.47 |
| 16 | 2.48 | 2.72 | 3.12 | 3.56 | 4.01 | 4.41 | 4.65 |
| 17 | 2.49 | 2.75 | 3.18 | 3.66 | 4.14 | 4.58 | 4.83 |
| 18 | 2.49 | 2.77 | 3.24 | 3.76 | 4.28 | 4.75 | 5.03 |

**Table S30.** Centiles of the myocardial thickness of the LV apical septal segment (segment 14) in boys and girls age 6-18 years (presented in mm).

|  |  |  | **BOYS** |  |  |  |  |
| --- | --- | --- | --- | --- | --- | --- | --- |
| **Age (years)** | **5th centile** | **10th centile** | **25th centile** | **50th centile** | **75th centile** | **90th centile** | **95th centile** |
| 6 | 2.25 | 2.38 | 2.58 | 2.78 | 2.96 | 3.11 | 3.19 |
| 7 | 2.21 | 2.39 | 2.66 | 2.93 | 3.17 | 3.38 | 3.50 |
| 8 | 2.20 | 2.40 | 2.73 | 3.07 | 3.40 | 3.68 | 3.85 |
| 9 | 2.21 | 2.43 | 2.80 | 3.22 | 3.66 | 4.06 | 4.30 |
| 10 | 2.30 | 2.51 | 2.89 | 3.38 | 3.94 | 4.51 | 4.89 |
| 11 | 2.44 | 2.64 | 3.02 | 3.55 | 4.23 | 5.02 | 5.60 |
| 12 | 2.62 | 2.81 | 3.18 | 3.73 | 4.49 | 5.47 | 6.29 |
| 13 | 2.79 | 2.98 | 3.36 | 3.91 | 4.67 | 5.65 | 6.46 |
| 14 | 2.95 | 3.15 | 3.56 | 4.11 | 4.80 | 5.58 | 6.14 |
| 15 | 3.07 | 3.32 | 3.76 | 4.31 | 4.91 | 5.51 | 5.89 |
| 16 | 3.19 | 3.49 | 3.98 | 4.52 | 5.05 | 5.52 | 5.80 |
| 17 | 3.32 | 3.69 | 4.23 | 4.75 | 5.21 | 5.60 | 5.81 |
| 18 | 3.55 | 3.95 | 4.50 | 4.99 | 5.40 | 5.71 | 5.88 |
|  |  |  | **GIRLS** |  |  |  |  |
| **Age (years)** | **5th centile** | **10th centile** | **25th centile** | **50th centile** | **75th centile** | **90th centile** | **95th centile** |
| 6 | 2.36 | 2.42 | 2.54 | 2.72 | 3.01 | 3.45 | 3.85 |
| 7 | 2.42 | 2.48 | 2.61 | 2.82 | 3.14 | 3.64 | 4.10 |
| 8 | 2.47 | 2.54 | 2.69 | 2.92 | 3.28 | 3.85 | 4.39 |
| 9 | 2.53 | 2.61 | 2.77 | 3.03 | 3.44 | 4.08 | 4.72 |
| 10 | 2.58 | 2.67 | 2.85 | 3.14 | 3.60 | 4.33 | 5.06 |
| 11 | 2.62 | 2.73 | 2.94 | 3.26 | 3.76 | 4.55 | 5.35 |
| 12 | 2.67 | 2.78 | 3.02 | 3.38 | 3.92 | 4.71 | 5.49 |
| 13 | 2.70 | 2.83 | 3.10 | 3.49 | 4.05 | 4.79 | 5.43 |
| 14 | 2.72 | 2.88 | 3.18 | 3.61 | 4.16 | 4.83 | 5.34 |
| 15 | 2.72 | 2.91 | 3.26 | 3.72 | 4.28 | 4.89 | 5.32 |
| 16 | 2.70 | 2.93 | 3.34 | 3.84 | 4.41 | 4.97 | 5.34 |
| 17 | 2.64 | 2.92 | 3.41 | 3.97 | 4.54 | 5.07 | 5.39 |
| 18 | 2.51 | 2.89 | 3.48 | 4.10 | 4.68 | 5.17 | 5.46 |

**Table S31.** Centiles of the myocardial thickness of the LV apical inferior segment (segment 15) in boys and girls age 6-18 years (presented in mm).

|  |  |  | **BOYS** |  |  |  |  |
| --- | --- | --- | --- | --- | --- | --- | --- |
| **Age (years)** | **5th centile** | **10th centile** | **25th centile** | **50th centile** | **75th centile** | **90th centile** | **95th centile** |
| 6 | 2.14 | 2.22 | 2.36 | 2.50 | 2.65 | 2.79 | 2.86 |
| 7 | 2.13 | 2.25 | 2.46 | 2.69 | 2.92 | 3.13 | 3.25 |
| 8 | 2.11 | 2.28 | 2.56 | 2.88 | 3.19 | 3.48 | 3.65 |
| 9 | 2.06 | 2.28 | 2.65 | 3.06 | 3.47 | 3.85 | 4.07 |
| 10 | 2.04 | 2.31 | 2.75 | 3.25 | 3.74 | 4.19 | 4.46 |
| 11 | 2.12 | 2.41 | 2.89 | 3.44 | 3.98 | 4.46 | 4.75 |
| 12 | 2.30 | 2.59 | 3.08 | 3.62 | 4.16 | 4.65 | 4.95 |
| 13 | 2.56 | 2.84 | 3.30 | 3.81 | 4.32 | 4.78 | 5.05 |
| 14 | 2.87 | 3.12 | 3.53 | 3.99 | 4.46 | 4.87 | 5.12 |
| 15 | 3.14 | 3.37 | 3.75 | 4.18 | 4.61 | 4.99 | 5.22 |
| 16 | 3.36 | 3.59 | 3.96 | 4.37 | 4.78 | 5.15 | 5.37 |
| 17 | 3.52 | 3.75 | 4.13 | 4.55 | 4.98 | 5.36 | 5.58 |
| 18 | 3.63 | 3.87 | 4.28 | 4.74 | 5.19 | 5.61 | 5.85 |
|  |  |  | **GIRLS** |  |  |  |  |
| **Age (years)** | **5th centile** | **10th centile** | **25th centile** | **50th centile** | **75th centile** | **90th centile** | **95th centile** |
| 6 | 2.01 | 2.20 | 2.52 | 2.87 | 3.22 | 3.54 | 3.73 |
| 7 | 2.06 | 2.26 | 2.59 | 2.96 | 3.33 | 3.66 | 3.86 |
| 8 | 2.10 | 2.31 | 2.66 | 3.05 | 3.43 | 3.78 | 3.99 |
| 9 | 2.14 | 2.36 | 2.73 | 3.13 | 3.54 | 3.91 | 4.12 |
| 10 | 2.18 | 2.41 | 2.79 | 3.22 | 3.65 | 4.03 | 4.26 |
| 11 | 2.22 | 2.46 | 2.86 | 3.31 | 3.75 | 4.16 | 4.40 |
| 12 | 2.25 | 2.50 | 2.93 | 3.40 | 3.86 | 4.29 | 4.54 |
| 13 | 2.28 | 2.55 | 2.99 | 3.48 | 3.97 | 4.42 | 4.68 |
| 14 | 2.31 | 2.59 | 3.05 | 3.57 | 4.09 | 4.55 | 4.83 |
| 15 | 2.34 | 2.63 | 3.12 | 3.66 | 4.20 | 4.68 | 4.98 |
| 16 | 2.36 | 2.67 | 3.18 | 3.74 | 4.31 | 4.82 | 5.13 |
| 17 | 2.38 | 2.70 | 3.24 | 3.83 | 4.43 | 4.96 | 5.28 |
| 18 | 2.40 | 2.73 | 3.30 | 3.92 | 4.54 | 5.10 | 5.44 |

**Table S32.** Centiles of the myocardial thickness of the LV apical lateral segment (segment 16) in boys and girls age 6-18 years (presented in mm).

|  |  |  | **BOYS** |  |  |  |  |
| --- | --- | --- | --- | --- | --- | --- | --- |
| **Age (years)** | **5th centile** | **10th centile** | **25th centile** | **50th centile** | **75th centile** | **90th centile** | **95th centile** |
| 6 | 2.11 | 2.16 | 2.27 | 2.44 | 2.70 | 3.09 | 3.42 |
| 7 | 2.19 | 2.25 | 2.38 | 2.58 | 2.90 | 3.37 | 3.80 |
| 8 | 2.27 | 2.34 | 2.49 | 2.73 | 3.12 | 3.73 | 4.29 |
| 9 | 2.34 | 2.43 | 2.61 | 2.91 | 3.39 | 4.18 | 4.94 |
| 10 | 2.41 | 2.52 | 2.74 | 3.10 | 3.69 | 4.67 | 5.70 |
| 11 | 2.48 | 2.61 | 2.88 | 3.30 | 3.95 | 5.00 | 6.09 |
| 12 | 2.55 | 2.70 | 3.01 | 3.47 | 4.10 | 4.96 | 5.69 |
| 13 | 2.61 | 2.79 | 3.14 | 3.63 | 4.24 | 4.93 | 5.44 |
| 14 | 2.67 | 2.88 | 3.29 | 3.80 | 4.39 | 5.00 | 5.40 |
| 15 | 2.73 | 2.99 | 3.44 | 3.98 | 4.55 | 5.11 | 5.46 |
| 16 | 2.80 | 3.10 | 3.60 | 4.16 | 4.73 | 5.26 | 5.57 |
| 17 | 2.86 | 3.21 | 3.76 | 4.36 | 4.92 | 5.42 | 5.71 |
| 18 | 2.91 | 3.32 | 3.94 | 4.56 | 5.12 | 5.60 | 5.87 |
|  |  |  | **GIRLS** |  |  |  |  |
| **Age (years)** | **5th centile** | **10th centile** | **25th centile** | **50th centile** | **75th centile** | **90th centile** | **95th centile** |
| 6 | 1.92 | 2.15 | 2.54 | 2.96 | 3.39 | 3.78 | 4.01 |
| 7 | 1.97 | 2.20 | 2.60 | 3.03 | 3.47 | 3.86 | 4.10 |
| 8 | 2.02 | 2.26 | 2.66 | 3.11 | 3.55 | 3.95 | 4.19 |
| 9 | 2.07 | 2.32 | 2.73 | 3.18 | 3.64 | 4.05 | 4.30 |
| 10 | 2.13 | 2.39 | 2.80 | 3.27 | 3.74 | 4.15 | 4.41 |
| 11 | 2.20 | 2.46 | 2.89 | 3.36 | 3.84 | 4.26 | 4.52 |
| 12 | 2.27 | 2.53 | 2.97 | 3.45 | 3.94 | 4.37 | 4.64 |
| 13 | 2.32 | 2.59 | 3.04 | 3.53 | 4.03 | 4.47 | 4.74 |
| 14 | 2.36 | 2.63 | 3.09 | 3.59 | 4.10 | 4.55 | 4.83 |
| 15 | 2.36 | 2.64 | 3.11 | 3.62 | 4.14 | 4.61 | 4.89 |
| 16 | 2.34 | 2.63 | 3.10 | 3.63 | 4.16 | 4.64 | 4.92 |
| 17 | 2.31 | 2.60 | 3.08 | 3.62 | 4.16 | 4.65 | 4.94 |
| 18 | 2.26 | 2.56 | 3.06 | 3.61 | 4.16 | 4.65 | 4.95 |

**Table S33.** Centiles of the myocardial thickness (presented in mm) of the RV basal lateral segment in boys and girls by BSA.

|  |  |  | **BOYS** |  |  |  |  |
| --- | --- | --- | --- | --- | --- | --- | --- |
| **BSA (m2)** | **5th centile** | **10th centile** | **25th centile** | **50th centile** | **75th centile** | **90th centile** | **95th centile** |
| 0.8 | 1.1 | 1.1 | 1.1 | 1.2 | 1.2 | 1.2 | 1.2 |
| 0.9 | 1.1 | 1.1 | 1.2 | 1.2 | 1.3 | 1.3 | 1.4 |
| 1.0 | 1.1 | 1.1 | 1.2 | 1.3 | 1.4 | 1.4 | 1.5 |
| 1.1 | 1.1 | 1.2 | 1.3 | 1.4 | 1.4 | 1.5 | 1.6 |
| 1.2 | 1.1 | 1.2 | 1.3 | 1.4 | 1.5 | 1.6 | 1.7 |
| 1.3 | 1.2 | 1.2 | 1.4 | 1.5 | 1.6 | 1.7 | 1.8 |
| 1.4 | 1.2 | 1.3 | 1.4 | 1.6 | 1.7 | 1.8 | 1.9 |
| 1.5 | 1.3 | 1.4 | 1.5 | 1.6 | 1.8 | 1.9 | 2.0 |
| 1.6 | 1.4 | 1.4 | 1.6 | 1.7 | 1.8 | 2.0 | 2.0 |
| 1.7 | 1.5 | 1.5 | 1.7 | 1.8 | 1.9 | 2.0 | 2.1 |
| 1.8 | 1.6 | 1.6 | 1.7 | 1.9 | 2.0 | 2.1 | 2.2 |
| 1.9 | 1.7 | 1.7 | 1.8 | 1.9 | 2.0 | 2.1 | 2.2 |
| 2.0 | 1.8 | 1.8 | 1.9 | 2.0 | 2.1 | 2.2 | 2.2 |
| 2.1 | 1.9 | 1.9 | 2.0 | 2.1 | 2.2 | 2.2 | 2.3 |
| 2.2 | 2.0 | 2.0 | 2.1 | 2.2 | 2.2 | 2.3 | 2.3 |
|  |  |  | **GIRLS** |  |  |  |  |
| **BSA (m2)** | **5th centile** | **10th centile** | **25th centile** | **50th centile** | **75th centile** | **90th centile** | **95th centile** |
| 0.8 | 1.0 | 1.1 | 1.1 | 1.2 | 1.2 | 1.3 | 1.3 |
| 0.9 | 1.0 | 1.1 | 1.1 | 1.2 | 1.3 | 1.3 | 1.4 |
| 1.0 | 1.1 | 1.1 | 1.2 | 1.2 | 1.3 | 1.4 | 1.4 |
| 1.1 | 1.1 | 1.1 | 1.2 | 1.3 | 1.4 | 1.5 | 1.5 |
| 1.2 | 1.1 | 1.2 | 1.3 | 1.4 | 1.5 | 1.5 | 1.6 |
| 1.3 | 1.2 | 1.2 | 1.3 | 1.4 | 1.5 | 1.6 | 1.7 |
| 1.4 | 1.2 | 1.3 | 1.4 | 1.5 | 1.6 | 1.7 | 1.8 |
| 1.5 | 1.3 | 1.4 | 1.5 | 1.6 | 1.7 | 1.8 | 1.9 |
| 1.6 | 1.4 | 1.5 | 1.6 | 1.7 | 1.8 | 1.9 | 2.0 |
| 1.7 | 1.5 | 1.6 | 1.7 | 1.8 | 1.9 | 2.0 | 2.1 |
| 1.8 | 1.6 | 1.7 | 1.8 | 1.9 | 2.0 | 2.1 | 2.2 |
| 1.9 | 1.7 | 1.8 | 1.9 | 2.0 | 2.1 | 2.2 | 2.3 |
| 2.0 | 1.8 | 1.9 | 2.0 | 2.1 | 2.2 | 2.3 | 2.4 |
| 2.1 | 1.9 | 2.0 | 2.1 | 2.2 | 2.3 | 2.4 | 2.5 |
| 2.2 | 2.0 | 2.1 | 2.2 | 2.3 | 2.4 | 2.5 | 2.5 |

**Table S34.** Centiles of the myocardial thickness (presented in mm) of the RV basal inferior segment in boys and girls by BSA.

|  |  |  | **BOYS** |  |  |  |  |
| --- | --- | --- | --- | --- | --- | --- | --- |
| **BSA (m2)** | **5th centile** | **10th centile** | **25th centile** | **50th centile** | **75th centile** | **90th centile** | **95th centile** |
| 0.8 | 1.0 | 1.0 | 1.0 | 1.1 | 1.2 | 1.2 | 1.3 |
| 0.9 | 1.0 | 1.0 | 1.1 | 1.1 | 1.2 | 1.3 | 1.3 |
| 1.0 | 1.0 | 1.1 | 1.1 | 1.2 | 1.3 | 1.4 | 1.4 |
| 1.1 | 1.1 | 1.1 | 1.2 | 1.3 | 1.4 | 1.5 | 1.5 |
| 1.2 | 1.1 | 1.2 | 1.2 | 1.3 | 1.4 | 1.6 | 1.6 |
| 1.3 | 1.2 | 1.2 | 1.3 | 1.4 | 1.5 | 1.7 | 1.7 |
| 1.4 | 1.2 | 1.3 | 1.4 | 1.5 | 1.6 | 1.8 | 1.9 |
| 1.5 | 1.3 | 1.3 | 1.4 | 1.5 | 1.7 | 1.9 | 2.0 |
| 1.6 | 1.3 | 1.4 | 1.5 | 1.6 | 1.8 | 1.9 | 2.1 |
| 1.7 | 1.4 | 1.5 | 1.6 | 1.7 | 1.9 | 2.0 | 2.1 |
| 1.8 | 1.5 | 1.6 | 1.7 | 1.8 | 2.0 | 2.1 | 2.2 |
| 1.9 | 1.6 | 1.7 | 1.8 | 1.9 | 2.0 | 2.2 | 2.3 |
| 2.0 | 1.7 | 1.8 | 1.9 | 2.0 | 2.1 | 2.2 | 2.3 |
| 2.1 | 1.9 | 1.9 | 2.0 | 2.1 | 2.2 | 2.3 | 2.4 |
| 2.2 | 2.0 | 2.0 | 2.1 | 2.2 | 2.3 | 2.4 | 2.5 |
|  |  |  | **GIRLS** |  |  |  |  |
| **BSA (m2)** | **5th centile** | **10th centile** | **25th centile** | **50th centile** | **75th centile** | **90th centile** | **95th centile** |
| 0.8 | 1.0 | 1.0 | 1.1 | 1.2 | 1.3 | 1.4 | 1.4 |
| 0.9 | 1.0 | 1.1 | 1.1 | 1.2 | 1.3 | 1.4 | 1.5 |
| 1.0 | 1.0 | 1.1 | 1.2 | 1.3 | 1.4 | 1.5 | 1.5 |
| 1.1 | 1.1 | 1.1 | 1.2 | 1.3 | 1.4 | 1.5 | 1.6 |
| 1.2 | 1.1 | 1.1 | 1.3 | 1.4 | 1.5 | 1.6 | 1.6 |
| 1.3 | 1.1 | 1.2 | 1.3 | 1.4 | 1.5 | 1.6 | 1.7 |
| 1.4 | 1.2 | 1.3 | 1.4 | 1.5 | 1.6 | 1.7 | 1.8 |
| 1.5 | 1.2 | 1.3 | 1.4 | 1.6 | 1.7 | 1.8 | 1.9 |
| 1.6 | 1.3 | 1.4 | 1.5 | 1.6 | 1.8 | 1.9 | 2.0 |
| 1.7 | 1.4 | 1.4 | 1.6 | 1.7 | 1.8 | 2.0 | 2.0 |
| 1.8 | 1.4 | 1.5 | 1.6 | 1.8 | 1.9 | 2.1 | 2.1 |
| 1.9 | 1.5 | 1.6 | 1.7 | 1.9 | 2.0 | 2.1 | 2.2 |
| 2.0 | 1.6 | 1.7 | 1.8 | 1.9 | 2.1 | 2.2 | 2.3 |
| 2.1 | 1.6 | 1.7 | 1.9 | 2.0 | 2.2 | 2.3 | 2.4 |
| 2.2 | 1.7 | 1.8 | 1.9 | 2.1 | 2.3 | 2.4 | 2.5 |

**Table S35.** Centiles of the myocardial thickness (presented in mm) of the RV midventricular lateral segment in boys and girls by BSA.

|  |  |  | **BOYS** |  |  |  |  |
| --- | --- | --- | --- | --- | --- | --- | --- |
| **BSA (m2)** | **5th centile** | **10th centile** | **25th centile** | **50th centile** | **75th centile** | **90th centile** | **95th centile** |
| 0.8 | 1.0 | 1.1 | 1.1 | 1.2 | 1.2 | 1.3 | 1.3 |
| 0.9 | 1.1 | 1.1 | 1.2 | 1.2 | 1.3 | 1.4 | 1.4 |
| 1.0 | 1.1 | 1.1 | 1.2 | 1.3 | 1.4 | 1.4 | 1.5 |
| 1.1 | 1.2 | 1.2 | 1.3 | 1.3 | 1.4 | 1.5 | 1.6 |
| 1.2 | 1.2 | 1.2 | 1.3 | 1.4 | 1.5 | 1.6 | 1.6 |
| 1.3 | 1.2 | 1.3 | 1.4 | 1.5 | 1.6 | 1.7 | 1.7 |
| 1.4 | 1.3 | 1.4 | 1.4 | 1.5 | 1.7 | 1.8 | 1.8 |
| 1.5 | 1.3 | 1.4 | 1.5 | 1.6 | 1.7 | 1.8 | 1.9 |
| 1.6 | 1.4 | 1.5 | 1.6 | 1.7 | 1.8 | 1.9 | 2.0 |
| 1.7 | 1.5 | 1.5 | 1.6 | 1.8 | 1.9 | 2.0 | 2.1 |
| 1.8 | 1.5 | 1.6 | 1.7 | 1.9 | 2.0 | 2.1 | 2.2 |
| 1.9 | 1.6 | 1.6 | 1.8 | 1.9 | 2.1 | 2.3 | 2.3 |
| 2.0 | 1.6 | 1.7 | 1.9 | 2.0 | 2.2 | 2.4 | 2.5 |
| 2.1 | 1.7 | 1.8 | 1.9 | 2.1 | 2.3 | 2.5 | 2.6 |
| 2.2 | 1.8 | 1.9 | 2.0 | 2.2 | 2.4 | 2.6 | 2.7 |
|  |  |  | **GIRLS** |  |  |  |  |
| **BSA (m2)** | **5th centile** | **10th centile** | **25th centile** | **50th centile** | **75th centile** | **90th centile** | **95th centile** |
| 0.8 | 1.0 | 1.1 | 1.2 | 1.2 | 1.3 | 1.4 | 1.5 |
| 0.9 | 1.0 | 1.1 | 1.2 | 1.3 | 1.4 | 1.4 | 1.5 |
| 1.0 | 1.0 | 1.1 | 1.2 | 1.3 | 1.4 | 1.5 | 1.5 |
| 1.1 | 1.1 | 1.1 | 1.2 | 1.3 | 1.4 | 1.5 | 1.5 |
| 1.2 | 1.1 | 1.1 | 1.2 | 1.3 | 1.4 | 1.5 | 1.6 |
| 1.3 | 1.1 | 1.2 | 1.3 | 1.4 | 1.5 | 1.6 | 1.7 |
| 1.4 | 1.2 | 1.3 | 1.4 | 1.5 | 1.6 | 1.7 | 1.8 |
| 1.5 | 1.3 | 1.4 | 1.5 | 1.6 | 1.7 | 1.8 | 1.9 |
| 1.6 | 1.4 | 1.5 | 1.6 | 1.7 | 1.8 | 1.9 | 2.0 |
| 1.7 | 1.5 | 1.6 | 1.7 | 1.8 | 1.9 | 2.0 | 2.1 |
| 1.8 | 1.6 | 1.6 | 1.7 | 1.9 | 2.0 | 2.1 | 2.2 |
| 1.9 | 1.6 | 1.7 | 1.8 | 2.0 | 2.1 | 2.2 | 2.3 |
| 2.0 | 1.7 | 1.8 | 1.9 | 2.0 | 2.2 | 2.3 | 2.4 |
| 2.1 | 1.8 | 1.9 | 2.0 | 2.1 | 2.3 | 2.4 | 2.5 |
| 2.2 | 1.9 | 1.9 | 2.1 | 2.2 | 2.4 | 2.5 | 2.6 |

**Table S36.** Centiles of the myocardial thickness (presented in mm) of the RV midventricular inferior segment in boys and girls by BSA.

|  |  |  | **BOYS** |  |  |  |  |
| --- | --- | --- | --- | --- | --- | --- | --- |
| **BSA (m2)** | **5th centile** | **10th centile** | **25th centile** | **50th centile** | **75th centile** | **90th centile** | **95th centile** |
| 0.8 | 1.0 | 1.1 | 1.1 | 1.1 | 1.1 | 1.2 | 1.2 |
| 0.9 | 1.1 | 1.1 | 1.1 | 1.2 | 1.2 | 1.3 | 1.3 |
| 1.0 | 1.1 | 1.1 | 1.2 | 1.2 | 1.3 | 1.3 | 1.4 |
| 1.1 | 1.1 | 1.2 | 1.2 | 1.3 | 1.4 | 1.5 | 1.5 |
| 1.2 | 1.1 | 1.2 | 1.3 | 1.4 | 1.5 | 1.6 | 1.6 |
| 1.3 | 1.2 | 1.2 | 1.3 | 1.4 | 1.6 | 1.7 | 1.7 |
| 1.4 | 1.2 | 1.3 | 1.4 | 1.5 | 1.6 | 1.8 | 1.8 |
| 1.5 | 1.3 | 1.3 | 1.5 | 1.6 | 1.7 | 1.8 | 1.9 |
| 1.6 | 1.3 | 1.4 | 1.5 | 1.7 | 1.8 | 1.9 | 2.0 |
| 1.7 | 1.4 | 1.5 | 1.6 | 1.7 | 1.9 | 2.0 | 2.1 |
| 1.8 | 1.5 | 1.5 | 1.7 | 1.8 | 1.9 | 2.1 | 2.1 |
| 1.9 | 1.6 | 1.6 | 1.7 | 1.9 | 2.0 | 2.1 | 2.2 |
| 2.0 | 1.6 | 1.7 | 1.8 | 1.9 | 2.0 | 2.1 | 2.2 |
| 2.1 | 1.7 | 1.8 | 1.9 | 2.0 | 2.1 | 2.2 | 2.2 |
| 2.2 | 1.8 | 1.9 | 1.9 | 2.0 | 2.1 | 2.2 | 2.2 |
|  |  |  | **GIRLS** |  |  |  |  |
| **BSA (m2)** | **5th centile** | **10th centile** | **25th centile** | **50th centile** | **75th centile** | **90th centile** | **95th centile** |
| 0.8 | 1.0 | 1.0 | 1.1 | 1.2 | 1.3 | 1.4 | 1.4 |
| 0.9 | 1.0 | 1.1 | 1.1 | 1.2 | 1.3 | 1.4 | 1.5 |
| 1.0 | 1.0 | 1.1 | 1.2 | 1.3 | 1.4 | 1.5 | 1.5 |
| 1.1 | 1.1 | 1.1 | 1.2 | 1.3 | 1.4 | 1.5 | 1.6 |
| 1.2 | 1.1 | 1.1 | 1.3 | 1.4 | 1.5 | 1.6 | 1.6 |
| 1.3 | 1.1 | 1.2 | 1.3 | 1.4 | 1.5 | 1.6 | 1.7 |
| 1.4 | 1.2 | 1.3 | 1.4 | 1.5 | 1.6 | 1.7 | 1.8 |
| 1.5 | 1.2 | 1.3 | 1.4 | 1.6 | 1.7 | 1.8 | 1.9 |
| 1.6 | 1.3 | 1.4 | 1.5 | 1.6 | 1.8 | 1.9 | 2.0 |
| 1.7 | 1.4 | 1.4 | 1.6 | 1.7 | 1.8 | 2.0 | 2.0 |
| 1.8 | 1.4 | 1.5 | 1.6 | 1.8 | 1.9 | 2.1 | 2.1 |
| 1.9 | 1.5 | 1.6 | 1.7 | 1.9 | 2.0 | 2.1 | 2.2 |
| 2.0 | 1.6 | 1.7 | 1.8 | 1.9 | 2.1 | 2.2 | 2.3 |
| 2.1 | 1.6 | 1.7 | 1.9 | 2.0 | 2.2 | 2.3 | 2.4 |
| 2.2 | 1.7 | 1.8 | 1.9 | 2.1 | 2.3 | 2.4 | 2.5 |

**Table S37.** Centiles of the myocardial thickness (presented in mm) of the RV apical lateral segment in boys and girls by BSA.

|  |  |  | **BOYS** |  |  |  |  |
| --- | --- | --- | --- | --- | --- | --- | --- |
| **BSA (m2)** | **5th centile** | **10th centile** | **25th centile** | **50th centile** | **75th centile** | **90th centile** | **95th centile** |
| 0.8 | 1.0 | 1.0 | 1.0 | 1.1 | 1.1 | 1.2 | 1.2 |
| 0.9 | 1.0 | 1.0 | 1.1 | 1.1 | 1.2 | 1.2 | 1.3 |
| 1.0 | 1.0 | 1.1 | 1.1 | 1.2 | 1.2 | 1.3 | 1.3 |
| 1.1 | 1.1 | 1.1 | 1.2 | 1.2 | 1.3 | 1.4 | 1.4 |
| 1.2 | 1.1 | 1.2 | 1.2 | 1.3 | 1.4 | 1.4 | 1.5 |
| 1.3 | 1.2 | 1.2 | 1.3 | 1.4 | 1.4 | 1.5 | 1.6 |
| 1.4 | 1.2 | 1.2 | 1.3 | 1.4 | 1.5 | 1.6 | 1.6 |
| 1.5 | 1.2 | 1.3 | 1.4 | 1.5 | 1.6 | 1.7 | 1.7 |
| 1.6 | 1.3 | 1.3 | 1.4 | 1.5 | 1.7 | 1.7 | 1.8 |
| 1.7 | 1.3 | 1.4 | 1.5 | 1.6 | 1.7 | 1.8 | 1.9 |
| 1.8 | 1.3 | 1.4 | 1.6 | 1.7 | 1.8 | 1.9 | 2.0 |
| 1.9 | 1.3 | 1.4 | 1.6 | 1.8 | 1.9 | 2.0 | 2.1 |
| 2.0 | 1.3 | 1.5 | 1.7 | 1.8 | 2.0 | 2.1 | 2.2 |
| 2.1 | 1.4 | 1.5 | 1.7 | 1.9 | 2.1 | 2.2 | 2.3 |
| 2.2 | 1.4 | 1.6 | 1.8 | 2.0 | 2.2 | 2.3 | 2.4 |
|  |  |  | **GIRLS** |  |  |  |  |
| **BSA (m2)** | **5th centile** | **10th centile** | **25th centile** | **50th centile** | **75th centile** | **90th centile** | **95th centile** |
| 0.8 | 1.0 | 1.0 | 1.1 | 1.1 | 1.2 | 1.2 | 1.2 |
| 0.9 | 1.0 | 1.0 | 1.1 | 1.1 | 1.2 | 1.3 | 1.3 |
| 1.0 | 1.0 | 1.1 | 1.1 | 1.2 | 1.2 | 1.3 | 1.3 |
| 1.1 | 1.1 | 1.1 | 1.2 | 1.2 | 1.3 | 1.4 | 1.4 |
| 1.2 | 1.1 | 1.1 | 1.2 | 1.3 | 1.3 | 1.4 | 1.5 |
| 1.3 | 1.1 | 1.1 | 1.2 | 1.3 | 1.4 | 1.5 | 1.5 |
| 1.4 | 1.1 | 1.2 | 1.3 | 1.4 | 1.5 | 1.5 | 1.6 |
| 1.5 | 1.2 | 1.2 | 1.3 | 1.4 | 1.5 | 1.6 | 1.7 |
| 1.6 | 1.2 | 1.3 | 1.4 | 1.5 | 1.6 | 1.7 | 1.8 |
| 1.7 | 1.2 | 1.3 | 1.4 | 1.5 | 1.7 | 1.8 | 1.9 |
| 1.8 | 1.2 | 1.3 | 1.5 | 1.6 | 1.8 | 1.9 | 2.0 |
| 1.9 | 1.3 | 1.4 | 1.5 | 1.7 | 1.9 | 2.0 | 2.1 |
| 2.0 | 1.3 | 1.4 | 1.6 | 1.8 | 1.9 | 2.1 | 2.2 |
| 2.1 | 1.3 | 1.5 | 1.6 | 1.9 | 2.1 | 2.2 | 2.3 |
| 2.2 | 1.3 | 1.5 | 1.7 | 1.9 | 2.2 | 2.4 | 2.5 |

**Table S38.** Centiles of the myocardial thickness (presented in mm) of the RV apical inferior segment in boys and girls by BSA.

|  |  |  | **BOYS** |  |  |  |  |
| --- | --- | --- | --- | --- | --- | --- | --- |
| **BSA (m2)** | **5th centile** | **10th centile** | **25th centile** | **50th centile** | **75th centile** | **90th centile** | **95th centile** |
| 0.8 | 0.9 | 0.9 | 1.0 | 1.0 | 1.1 | 1.2 | 1.3 |
| 0.9 | 1.0 | 1.0 | 1.0 | 1.1 | 1.2 | 1.3 | 1.4 |
| 1.0 | 1.0 | 1.0 | 1.1 | 1.1 | 1.2 | 1.3 | 1.4 |
| 1.1 | 1.1 | 1.1 | 1.1 | 1.2 | 1.2 | 1.3 | 1.4 |
| 1.2 | 1.1 | 1.1 | 1.2 | 1.2 | 1.3 | 1.4 | 1.4 |
| 1.3 | 1.1 | 1.2 | 1.2 | 1.3 | 1.4 | 1.4 | 1.5 |
| 1.4 | 1.2 | 1.2 | 1.3 | 1.3 | 1.4 | 1.5 | 1.5 |
| 1.5 | 1.2 | 1.2 | 1.3 | 1.4 | 1.5 | 1.6 | 1.6 |
| 1.6 | 1.2 | 1.3 | 1.4 | 1.5 | 1.6 | 1.7 | 1.8 |
| 1.7 | 1.2 | 1.3 | 1.4 | 1.5 | 1.7 | 1.8 | 1.9 |
| 1.8 | 1.3 | 1.3 | 1.5 | 1.6 | 1.8 | 1.9 | 2.0 |
| 1.9 | 1.4 | 1.4 | 1.6 | 1.7 | 1.8 | 1.9 | 2.0 |
| 2.0 | 1.5 | 1.6 | 1.7 | 1.8 | 1.9 | 2.0 | 2.0 |
| 2.1 | 1.7 | 1.7 | 1.8 | 1.8 | 1.9 | 2.0 | 2.0 |
| 2.2 | 1.8 | 1.8 | 1.9 | 1.9 | 2.0 | 2.0 | 2.0 |
|  |  |  | **GIRLS** |  |  |  |  |
| **BSA (m2)** | **5th centile** | **10th centile** | **25th centile** | **50th centile** | **75th centile** | **90th centile** | **95th centile** |
| 0.8 | 1.0 | 1.0 | 1.0 | 1.1 | 1.1 | 1.2 | 1.2 |
| 0.9 | 1.0 | 1.0 | 1.1 | 1.1 | 1.2 | 1.2 | 1.3 |
| 1.0 | 1.0 | 1.1 | 1.1 | 1.1 | 1.2 | 1.3 | 1.3 |
| 1.1 | 1.1 | 1.1 | 1.1 | 1.2 | 1.3 | 1.3 | 1.4 |
| 1.2 | 1.1 | 1.1 | 1.2 | 1.2 | 1.3 | 1.4 | 1.5 |
| 1.3 | 1.1 | 1.2 | 1.2 | 1.3 | 1.4 | 1.5 | 1.5 |
| 1.4 | 1.2 | 1.2 | 1.2 | 1.3 | 1.4 | 1.5 | 1.6 |
| 1.5 | 1.2 | 1.2 | 1.3 | 1.4 | 1.5 | 1.6 | 1.7 |
| 1.6 | 1.2 | 1.3 | 1.3 | 1.4 | 1.5 | 1.7 | 1.8 |
| 1.7 | 1.3 | 1.3 | 1.4 | 1.5 | 1.6 | 1.7 | 1.8 |
| 1.8 | 1.3 | 1.3 | 1.4 | 1.5 | 1.7 | 1.8 | 1.9 |
| 1.9 | 1.3 | 1.4 | 1.5 | 1.6 | 1.7 | 1.9 | 2.0 |
| 2.0 | 1.3 | 1.4 | 1.5 | 1.7 | 1.8 | 2.0 | 2.1 |
| 2.1 | 1.3 | 1.4 | 1.6 | 1.7 | 1.9 | 2.1 | 2.2 |
| 2.2 | 1.3 | 1.4 | 1.6 | 1.8 | 2.0 | 2.1 | 2.2 |

**Table S39.** Centiles of the myocardial thickness of the RV basal lateral segment in boys and girls age 6-18 years (presented in mm).

|  |  |  | **BOYS** |  |  |  |  |
| --- | --- | --- | --- | --- | --- | --- | --- |
| **Age (years)** | **5th centile** | **10th centile** | **25th centile** | **50th centile** | **75th centile** | **90th centile** | **95th centile** |
| 6 | 1.27 | 1.28 | 1.31 | 1.35 | 1.41 | 1.48 | 1.55 |
| 7 | 1.22 | 1.23 | 1.27 | 1.32 | 1.40 | 1.50 | 1.59 |
| 8 | 1.17 | 1.19 | 1.23 | 1.29 | 1.39 | 1.52 | 1.64 |
| 9 | 1.13 | 1.16 | 1.21 | 1.28 | 1.39 | 1.56 | 1.71 |
| 10 | 1.12 | 1.15 | 1.21 | 1.30 | 1.43 | 1.61 | 1.78 |
| 11 | 1.15 | 1.19 | 1.26 | 1.35 | 1.48 | 1.63 | 1.76 |
| 12 | 1.24 | 1.28 | 1.36 | 1.45 | 1.56 | 1.68 | 1.76 |
| 13 | 1.38 | 1.42 | 1.50 | 1.60 | 1.70 | 1.79 | 1.85 |
| 14 | 1.51 | 1.56 | 1.65 | 1.75 | 1.85 | 1.94 | 1.99 |
| 15 | 1.57 | 1.64 | 1.75 | 1.87 | 1.97 | 2.06 | 2.11 |
| 16 | 1.51 | 1.63 | 1.78 | 1.92 | 2.03 | 2.12 | 2.17 |
| 17 | 1.44 | 1.59 | 1.79 | 1.95 | 2.08 | 2.17 | 2.22 |
| 18 | 1.47 | 1.63 | 1.84 | 2.02 | 2.15 | 2.24 | 2.29 |
|  |  |  | **GIRLS** |  |  |  |  |
| **Age (years)** | **5th centile** | **10th centile** | **25th centile** | **50th centile** | **75th centile** | **90th centile** | **95th centile** |
| 6 | 1.02 | 1.04 | 1.07 | 1.11 | 1.18 | 1.27 | 1.35 |
| 7 | 1.06 | 1.08 | 1.11 | 1.16 | 1.24 | 1.34 | 1.43 |
| 8 | 1.10 | 1.11 | 1.15 | 1.21 | 1.30 | 1.43 | 1.53 |
| 9 | 1.13 | 1.15 | 1.20 | 1.27 | 1.37 | 1.52 | 1.65 |
| 10 | 1.17 | 1.20 | 1.25 | 1.33 | 1.45 | 1.63 | 1.79 |
| 11 | 1.21 | 1.24 | 1.30 | 1.39 | 1.54 | 1.76 | 1.96 |
| 12 | 1.25 | 1.29 | 1.36 | 1.46 | 1.64 | 1.90 | 2.15 |
| 13 | 1.29 | 1.33 | 1.41 | 1.54 | 1.73 | 2.02 | 2.30 |
| 14 | 1.33 | 1.38 | 1.47 | 1.61 | 1.79 | 2.04 | 2.26 |
| 15 | 1.36 | 1.42 | 1.53 | 1.67 | 1.84 | 2.03 | 2.17 |
| 16 | 1.40 | 1.46 | 1.59 | 1.73 | 1.89 | 2.05 | 2.15 |
| 17 | 1.42 | 1.51 | 1.65 | 1.80 | 1.95 | 2.08 | 2.16 |
| 18 | 1.43 | 1.55 | 1.71 | 1.87 | 2.01 | 2.13 | 2.20 |

**Table S40.** Centiles of the myocardial thickness of the RV basal inferior segment in boys and girls age 6-18 years (presented in mm).

|  |  |  | **BOYS** |  |  |  |  |
| --- | --- | --- | --- | --- | --- | --- | --- |
| **Age (years)** | **5th centile** | **10th centile** | **25th centile** | **50th centile** | **75th centile** | **90th centile** | **95th centile** |
| 6 | 0.99 | 1.00 | 1.03 | 1.06 | 1.10 | 1.13 | 1.16 |
| 7 | 1.02 | 1.04 | 1.08 | 1.12 | 1.18 | 1.23 | 1.27 |
| 8 | 1.05 | 1.07 | 1.12 | 1.19 | 1.26 | 1.34 | 1.39 |
| 9 | 1.07 | 1.10 | 1.17 | 1.25 | 1.35 | 1.45 | 1.52 |
| 10 | 1.08 | 1.13 | 1.21 | 1.32 | 1.45 | 1.57 | 1.65 |
| 11 | 1.09 | 1.15 | 1.27 | 1.40 | 1.54 | 1.68 | 1.77 |
| 12 | 1.13 | 1.21 | 1.33 | 1.48 | 1.63 | 1.78 | 1.88 |
| 13 | 1.22 | 1.29 | 1.41 | 1.56 | 1.72 | 1.89 | 1.99 |
| 14 | 1.33 | 1.40 | 1.51 | 1.65 | 1.82 | 1.99 | 2.11 |
| 15 | 1.44 | 1.50 | 1.61 | 1.74 | 1.91 | 2.08 | 2.20 |
| 16 | 1.52 | 1.58 | 1.70 | 1.84 | 1.99 | 2.14 | 2.23 |
| 17 | 1.55 | 1.65 | 1.80 | 1.95 | 2.08 | 2.19 | 2.25 |
| 18 | 1.59 | 1.73 | 1.91 | 2.06 | 2.18 | 2.27 | 2.31 |
|  |  |  | **GIRLS** |  |  |  |  |
| **Age (years)** | **5th centile** | **10th centile** | **25th centile** | **50th centile** | **75th centile** | **90th centile** | **95th centile** |
| 6 | 0.96 | 1.02 | 1.11 | 1.22 | 1.33 | 1.43 | 1.49 |
| 7 | 0.99 | 1.05 | 1.15 | 1.27 | 1.38 | 1.49 | 1.55 |
| 8 | 1.02 | 1.09 | 1.19 | 1.31 | 1.43 | 1.54 | 1.60 |
| 9 | 1.06 | 1.12 | 1.23 | 1.36 | 1.48 | 1.59 | 1.66 |
| 10 | 1.09 | 1.16 | 1.27 | 1.40 | 1.53 | 1.65 | 1.72 |
| 11 | 1.12 | 1.19 | 1.31 | 1.45 | 1.58 | 1.70 | 1.77 |
| 12 | 1.15 | 1.23 | 1.35 | 1.49 | 1.63 | 1.76 | 1.83 |
| 13 | 1.18 | 1.26 | 1.39 | 1.54 | 1.68 | 1.81 | 1.89 |
| 14 | 1.21 | 1.29 | 1.43 | 1.58 | 1.73 | 1.87 | 1.95 |
| 15 | 1.24 | 1.33 | 1.47 | 1.63 | 1.78 | 1.93 | 2.01 |
| 16 | 1.27 | 1.36 | 1.51 | 1.67 | 1.84 | 1.98 | 2.07 |
| 17 | 1.30 | 1.39 | 1.55 | 1.72 | 1.89 | 2.04 | 2.13 |
| 18 | 1.33 | 1.42 | 1.58 | 1.76 | 1.94 | 2.10 | 2.20 |

**Table S41.** Centiles of the myocardial thickness of the RV midventricular lateral segment in boys and girls age 6-18 years (presented in mm).

|  |  |  | **BOYS** |  |  |  |  |
| --- | --- | --- | --- | --- | --- | --- | --- |
| **Age (years)** | **5th centile** | **10th centile** | **25th centile** | **50th centile** | **75th centile** | **90th centile** | **95th centile** |
| 6.00 | 0.95 | 0.98 | 1.04 | 1.12 | 1.22 | 1.33 | 1.41 |
| 7.00 | 1.01 | 1.04 | 1.10 | 1.19 | 1.29 | 1.39 | 1.47 |
| 8.00 | 1.06 | 1.10 | 1.17 | 1.25 | 1.35 | 1.46 | 1.53 |
| 9.00 | 1.12 | 1.16 | 1.23 | 1.32 | 1.42 | 1.53 | 1.60 |
| 10.00 | 1.18 | 1.22 | 1.30 | 1.39 | 1.50 | 1.60 | 1.67 |
| 11.00 | 1.24 | 1.28 | 1.37 | 1.47 | 1.58 | 1.68 | 1.75 |
| 12.00 | 1.30 | 1.35 | 1.44 | 1.54 | 1.66 | 1.77 | 1.84 |
| 13.00 | 1.35 | 1.41 | 1.51 | 1.62 | 1.75 | 1.87 | 1.94 |
| 14.00 | 1.40 | 1.47 | 1.58 | 1.71 | 1.84 | 1.97 | 2.05 |
| 15.00 | 1.44 | 1.52 | 1.64 | 1.79 | 1.94 | 2.08 | 2.17 |
| 16.00 | 1.47 | 1.56 | 1.71 | 1.88 | 2.05 | 2.21 | 2.30 |
| 17.00 | 1.48 | 1.59 | 1.77 | 1.97 | 2.17 | 2.34 | 2.45 |
| 18.00 | 1.47 | 1.60 | 1.82 | 2.06 | 2.29 | 2.49 | 2.61 |
|  |  |  | **GIRLS** |  |  |  |  |
| **Age (years)** | **5th centile** | **10th centile** | **25th centile** | **50th centile** | **75th centile** | **90th centile** | **95th centile** |
| 6.00 | 0.95 | 1.00 | 1.09 | 1.19 | 1.29 | 1.38 | 1.43 |
| 7.00 | 0.99 | 1.05 | 1.14 | 1.25 | 1.35 | 1.44 | 1.50 |
| 8.00 | 1.03 | 1.09 | 1.19 | 1.30 | 1.41 | 1.51 | 1.57 |
| 9.00 | 1.07 | 1.13 | 1.24 | 1.35 | 1.47 | 1.57 | 1.63 |
| 10.00 | 1.11 | 1.18 | 1.29 | 1.41 | 1.53 | 1.64 | 1.70 |
| 11.00 | 1.15 | 1.22 | 1.33 | 1.46 | 1.59 | 1.70 | 1.77 |
| 12.00 | 1.19 | 1.26 | 1.38 | 1.52 | 1.65 | 1.77 | 1.84 |
| 13.00 | 1.22 | 1.30 | 1.43 | 1.57 | 1.71 | 1.84 | 1.91 |
| 14.00 | 1.26 | 1.34 | 1.47 | 1.62 | 1.77 | 1.90 | 1.98 |
| 15.00 | 1.30 | 1.38 | 1.52 | 1.68 | 1.83 | 1.97 | 2.06 |
| 16.00 | 1.33 | 1.42 | 1.57 | 1.73 | 1.89 | 2.04 | 2.13 |
| 17.00 | 1.36 | 1.46 | 1.61 | 1.78 | 1.96 | 2.11 | 2.21 |
| 18.00 | 1.40 | 1.49 | 1.66 | 1.84 | 2.02 | 2.18 | 2.28 |

**Table S42.** Centiles of the myocardial thickness of the RV midventricular inferior segment in boys and girls age 6-18 years (presented in mm).

|  |  |  | **BOYS** |  |  |  |  |
| --- | --- | --- | --- | --- | --- | --- | --- |
| **Age (years)** | **5th centile** | **10th centile** | **25th centile** | **50th centile** | **75th centile** | **90th centile** | **95th centile** |
| 6.00 | 0.82 | 0.88 | 0.97 | 1.08 | 1.19 | 1.29 | 1.35 |
| 7.00 | 0.88 | 0.94 | 1.04 | 1.15 | 1.27 | 1.37 | 1.43 |
| 8.00 | 0.95 | 1.01 | 1.11 | 1.23 | 1.34 | 1.45 | 1.51 |
| 9.00 | 1.01 | 1.07 | 1.18 | 1.30 | 1.42 | 1.53 | 1.59 |
| 10.00 | 1.07 | 1.14 | 1.25 | 1.37 | 1.49 | 1.61 | 1.67 |
| 11.00 | 1.14 | 1.20 | 1.32 | 1.44 | 1.57 | 1.68 | 1.75 |
| 12.00 | 1.20 | 1.27 | 1.39 | 1.52 | 1.65 | 1.76 | 1.83 |
| 13.00 | 1.26 | 1.33 | 1.45 | 1.59 | 1.72 | 1.84 | 1.92 |
| 14.00 | 1.32 | 1.40 | 1.52 | 1.66 | 1.80 | 1.93 | 2.00 |
| 15.00 | 1.38 | 1.46 | 1.59 | 1.73 | 1.88 | 2.01 | 2.08 |
| 16.00 | 1.45 | 1.53 | 1.66 | 1.81 | 1.95 | 2.09 | 2.17 |
| 17.00 | 1.51 | 1.59 | 1.73 | 1.88 | 2.03 | 2.17 | 2.25 |
| 18.00 | 1.57 | 1.65 | 1.79 | 1.95 | 2.11 | 2.25 | 2.33 |
|  |  |  | **GIRLS** |  |  |  |  |
| **Age (years)** | **5th centile** | **10th centile** | **25th centile** | **50th centile** | **75th centile** | **90th centile** | **95th centile** |
| 6.00 | 0.96 | 1.02 | 1.11 | 1.22 | 1.33 | 1.43 | 1.49 |
| 7.00 | 0.99 | 1.05 | 1.15 | 1.27 | 1.38 | 1.49 | 1.55 |
| 8.00 | 1.02 | 1.09 | 1.19 | 1.31 | 1.43 | 1.54 | 1.60 |
| 9.00 | 1.06 | 1.12 | 1.23 | 1.36 | 1.48 | 1.59 | 1.66 |
| 10.00 | 1.09 | 1.16 | 1.27 | 1.40 | 1.53 | 1.65 | 1.72 |
| 11.00 | 1.12 | 1.19 | 1.31 | 1.45 | 1.58 | 1.70 | 1.77 |
| 12.00 | 1.15 | 1.23 | 1.35 | 1.49 | 1.63 | 1.76 | 1.83 |
| 13.00 | 1.18 | 1.26 | 1.39 | 1.54 | 1.68 | 1.81 | 1.89 |
| 14.00 | 1.21 | 1.29 | 1.43 | 1.58 | 1.73 | 1.87 | 1.95 |
| 15.00 | 1.24 | 1.33 | 1.47 | 1.63 | 1.78 | 1.93 | 2.01 |
| 16.00 | 1.27 | 1.36 | 1.51 | 1.67 | 1.84 | 1.98 | 2.07 |
| 17.00 | 1.30 | 1.39 | 1.55 | 1.72 | 1.89 | 2.04 | 2.13 |
| 18.00 | 1.33 | 1.42 | 1.58 | 1.76 | 1.94 | 2.10 | 2.20 |

**Table S43.** Centiles of the myocardial thickness of the RV apical lateral segment in boys and girls age 6-18 years (presented in mm).

|  |  |  | **BOYS** |  |  |  |  |
| --- | --- | --- | --- | --- | --- | --- | --- |
| **Age (years)** | **5th centile** | **10th centile** | **25th centile** | **50th centile** | **75th centile** | **90th centile** | **95th centile** |
| 6.00 | 0.97 | 1.01 | 1.05 | 1.09 | 1.11 | 1.13 | 1.14 |
| 7.00 | 0.99 | 1.03 | 1.09 | 1.14 | 1.18 | 1.20 | 1.21 |
| 8.00 | 0.98 | 1.04 | 1.12 | 1.19 | 1.24 | 1.27 | 1.29 |
| 9.00 | 0.93 | 1.02 | 1.13 | 1.23 | 1.30 | 1.35 | 1.38 |
| 10.00 | 1.01 | 1.07 | 1.17 | 1.28 | 1.37 | 1.45 | 1.49 |
| 11.00 | 1.11 | 1.16 | 1.24 | 1.34 | 1.45 | 1.56 | 1.63 |
| 12.00 | 1.18 | 1.22 | 1.30 | 1.40 | 1.53 | 1.66 | 1.76 |
| 13.00 | 1.23 | 1.28 | 1.36 | 1.47 | 1.60 | 1.75 | 1.86 |
| 14.00 | 1.28 | 1.33 | 1.42 | 1.54 | 1.68 | 1.83 | 1.93 |
| 15.00 | 1.31 | 1.37 | 1.48 | 1.61 | 1.76 | 1.90 | 1.99 |
| 16.00 | 1.33 | 1.41 | 1.54 | 1.69 | 1.84 | 1.98 | 2.06 |
| 17.00 | 1.32 | 1.43 | 1.60 | 1.77 | 1.93 | 2.06 | 2.13 |
| 18.00 | 1.29 | 1.46 | 1.67 | 1.86 | 2.02 | 2.13 | 2.20 |
|  |  |  | **GIRLS** |  |  |  |  |
| **Age (years)** | **5th centile** | **10th centile** | **25th centile** | **50th centile** | **75th centile** | **90th centile** | **95th centile** |
| 6.00 | 1.00 | 1.03 | 1.08 | 1.14 | 1.20 | 1.26 | 1.30 |
| 7.00 | 1.02 | 1.05 | 1.10 | 1.17 | 1.24 | 1.31 | 1.35 |
| 8.00 | 1.03 | 1.07 | 1.13 | 1.20 | 1.28 | 1.36 | 1.41 |
| 9.00 | 1.05 | 1.09 | 1.16 | 1.24 | 1.32 | 1.41 | 1.46 |
| 10.00 | 1.07 | 1.11 | 1.18 | 1.27 | 1.37 | 1.46 | 1.52 |
| 11.00 | 1.09 | 1.13 | 1.21 | 1.31 | 1.41 | 1.52 | 1.58 |
| 12.00 | 1.11 | 1.15 | 1.24 | 1.34 | 1.46 | 1.58 | 1.65 |
| 13.00 | 1.13 | 1.18 | 1.27 | 1.38 | 1.51 | 1.64 | 1.73 |
| 14.00 | 1.14 | 1.20 | 1.29 | 1.42 | 1.56 | 1.71 | 1.81 |
| 15.00 | 1.16 | 1.22 | 1.32 | 1.46 | 1.62 | 1.78 | 1.89 |
| 16.00 | 1.18 | 1.24 | 1.35 | 1.50 | 1.67 | 1.86 | 1.98 |
| 17.00 | 1.19 | 1.26 | 1.38 | 1.54 | 1.73 | 1.94 | 2.09 |
| 18.00 | 1.21 | 1.28 | 1.41 | 1.58 | 1.80 | 2.03 | 2.20 |

**Table S44.** Centiles of the myocardial thickness of the RV apical inferior segment in boys and girls age 6-18 years (presented in mm).

|  |  |  | **BOYS** |  |  |  |  |
| --- | --- | --- | --- | --- | --- | --- | --- |
| **Age (years)** | **5th centile** | **10th centile** | **25th centile** | **50th centile** | **75th centile** | **90th centile** | **95th centile** |
| 6.00 | 0.89 | 0.91 | 0.95 | 1.00 | 1.08 | 1.17 | 1.25 |
| 7.00 | 0.93 | 0.95 | 1.00 | 1.06 | 1.13 | 1.23 | 1.31 |
| 8.00 | 0.97 | 0.99 | 1.04 | 1.11 | 1.19 | 1.30 | 1.38 |
| 9.00 | 1.01 | 1.04 | 1.09 | 1.16 | 1.25 | 1.36 | 1.44 |
| 10.00 | 1.05 | 1.08 | 1.14 | 1.22 | 1.31 | 1.43 | 1.51 |
| 11.00 | 1.09 | 1.13 | 1.19 | 1.28 | 1.38 | 1.50 | 1.58 |
| 12.00 | 1.13 | 1.17 | 1.24 | 1.34 | 1.45 | 1.57 | 1.66 |
| 13.00 | 1.17 | 1.22 | 1.30 | 1.40 | 1.52 | 1.65 | 1.73 |
| 14.00 | 1.21 | 1.27 | 1.36 | 1.47 | 1.60 | 1.73 | 1.81 |
| 15.00 | 1.25 | 1.31 | 1.42 | 1.54 | 1.68 | 1.81 | 1.90 |
| 16.00 | 1.29 | 1.36 | 1.48 | 1.62 | 1.76 | 1.90 | 1.99 |
| 17.00 | 1.33 | 1.41 | 1.54 | 1.70 | 1.85 | 1.99 | 2.08 |
| 18.00 | 1.36 | 1.45 | 1.61 | 1.78 | 1.94 | 2.09 | 2.18 |
|  |  |  | **GIRLS** |  |  |  |  |
| **Age (years)** | **5th centile** | **10th centile** | **25th centile** | **50th centile** | **75th centile** | **90th centile** | **95th centile** |
| 6.00 | 0.99 | 1.01 | 1.04 | 1.09 | 1.14 | 1.20 | 1.23 |
| 7.00 | 1.02 | 1.04 | 1.07 | 1.12 | 1.18 | 1.24 | 1.28 |
| 8.00 | 1.04 | 1.06 | 1.10 | 1.15 | 1.22 | 1.29 | 1.34 |
| 9.00 | 1.06 | 1.09 | 1.13 | 1.19 | 1.26 | 1.34 | 1.39 |
| 10.00 | 1.09 | 1.11 | 1.16 | 1.22 | 1.30 | 1.39 | 1.45 |
| 11.00 | 1.11 | 1.14 | 1.19 | 1.26 | 1.35 | 1.44 | 1.52 |
| 12.00 | 1.13 | 1.16 | 1.22 | 1.30 | 1.39 | 1.50 | 1.58 |
| 13.00 | 1.16 | 1.19 | 1.25 | 1.34 | 1.44 | 1.57 | 1.66 |
| 14.00 | 1.18 | 1.22 | 1.28 | 1.38 | 1.49 | 1.63 | 1.74 |
| 15.00 | 1.20 | 1.24 | 1.32 | 1.42 | 1.55 | 1.70 | 1.82 |
| 16.00 | 1.22 | 1.27 | 1.35 | 1.46 | 1.60 | 1.78 | 1.92 |
| 17.00 | 1.25 | 1.29 | 1.38 | 1.50 | 1.66 | 1.86 | 2.02 |
| 18.00 | 1.27 | 1.32 | 1.41 | 1.55 | 1.73 | 1.95 | 2.13 |

**Table S45.** Centiles of the myocardial mass of the LV by age and gender.

|  |  |  | **BOYS** |  |  |  |  |
| --- | --- | --- | --- | --- | --- | --- | --- |
| **Age (years)** | **5th centile** | **10th centile** | **25th centile** | **50th centile** | **75th centile** | **90th centile** | **95th centile** |
| 6.00 | 1974 | 23.12 | 28.77 | 35.04 | 41.31 | 46.96 | 50.34 |
| 7.00 | 22.88 | 26.50 | 32.53 | 39,24 | 45.95 | 51.99 | 55.60 |
| 8.00 | 26.06 | 29.91 | 36.35 | 43.50 | 50.65 | 57.08 | 60.93 |
| 9.00 | 29.55 | 33.65 | 40.51 | 48.13 | 55.75 | 62.61 | 66.71 |
| 10.00 | 33.76 | 38.13 | 45.44 | 53.56 | 61.68 | 68.98 | 73.36 |
| 11.00 | 39.31 | 43.97 | 51.76 | 60.41 | 69.06 | 76.85 | 81.51 |
| 12.00 | 46.69 | 51.66 | 59.96 | 69.17 | 78.39 | 86.69 | 91.66 |
| 13.00 | 55.49 | 60.78 | 69.62 | 79.44 | 89.27 | 98.11 | 103.40 |
| 14.00 | 64.36 | 70.00 | 79.42 | 89.89 | 100.35 | 109.78 | 115.41 |
| 15.00 | 7226 | 78.26 | 88.30 | 99.46 | 110.61 | 120.65 | 126.66 |
| 16.00 | 78,84 | 85.24 | 95.94 | 107.82 | 119.71 | 130.41 | 136.81 |
| 17.00 | 84.24 | 91.06 | 102.46 | 115.12 | 127.79 | 139.18 | 146.01 |
| 18.00 | 88.82 | 96.09 | 108.23 | 121.73 | 135.22 | 147.37 | 154.64 |
|  |  |  | **GIRLS** |  |  |  |  |
| **Age (years)** | **5th centile** | **10th centile** | **25th centile** | **50th centile** | **75th centile** | **90th centile** | **95th centile** |
| 6.00 | 23.79 | 26.14 | 30.18 | 34.83 | 39.65 | 44.10 | 46.83 |
| 7.00 | 26.17 | 28.70 | 33.10 | 38.27 | 43.70 | 48.82 | 51.98 |
| 8.00 | 28.80 | 31.51 | 36.31 | 42.04 | 48.18 | 54.06 | 57.74 |
| 9.00 | 31.71 | 34.63 | 39.86 | 46.22 | 53.17 | 59.95 | 64.24 |
| 10.00 | 34.85 | 37.99 | 43.69 | 50.73 | 58.59 | 66.39 | 71.42 |
| 11.00 | 38.11 | 41.46 | 47.62 | 55.39 | 64.22 | 73.18 | 79.05 |
| 12.00 | 41.32 | 44.87 | 51.49 | 59.97 | 69.83 | 80.06 | 86.87 |
| 13.00 | 44.41 | 48.14 | 55.17 | 64.36 | 75.27 | 86.86 | 94.75 |
| 14.00 | 47.29 | 51.18 | 58.59 | 68.44 | 80.42 | 93.48 | 102.55 |
| 15.00 | 49.85 | 53.85 | 61.58 | 72.04 | 85.06 | 99.66 | 110.05 |
| 16.00 | 51.94 | 56.02 | 63.98 | 74.95 | 88.96 | 105.14 | 117.00 |
| 17.00 | 53.63 | 57.75 | 65.88 | 77.28 | 92.22 | 110.06 | 123.55 |
| 18.00 | 55.14 | 59.28 | 67.54 | 79.34 | 95.21 | 114.87 | 130.29 |

**Table S46.** Centiles of the myocardial mass of the RV by age and gender.

|  |  |  | **BOYS** |  |  |  |  |
| --- | --- | --- | --- | --- | --- | --- | --- |
| **Age (years)** | **5th centile** | **10th centile** | **25th centile** | **50th centile** | **75th centile** | **90th centile** | **95th centile** |
| 6.00 | 4.61 | 6.21 | 8.88 | 11.85 | 14.82 | 17.50 | 19.10 |
| 7.00 | 7.40 | 9.19 | 11.94 | 15.09 | 18.24 | 21.08 | 22.78 |
| 8.00 | 10.19 | 11.99 | 14.99 | 18.33 | 21.67 | 24.67 | 26.47 |
| 9.00 | 12.96 | 14.86 | 18.04 | 21.57 | 25.10 | 28.27 | 30.18 |
| 10.00 | 15.70 | 17.71 | 21.07 | 24.81 | 28.54 | 31.90 | 33.91 |
| 11.00 | 18.41 | 20.54 | 24.09 | 28.04 | 31.99 | 35.55 | 37.68 |
| 12.00 | 21.09 | 23.34 | 27.10 | 31.28 | 35.46 | 39.22 | 41.48 |
| 13.00 | 23.73 | 26.12 | 30.10 | 34.52 | 38.94 | 42.92 | 45.31 |
| 14.00 | 26.35 | 28.87 | 33.08 | 37.76 | 42.44 | 46.65 | 49.17 |
| 15.00 | 28.92 | 31.59 | 36.05 | 41.00 | 45.95 | 50.40 | 53.07 |
| 16.00 | 31.46 | 34.28 | 39.00 | 44.23 | 49.47 | 54.19 | 57.01 |
| 17.00 | 33.96 | 36.94 | 41.93 | 47.47 | 53.01 | 58.00 | 60.99 |
| 18.00 | 36.41 | 39.57 | 44.85 | 50.71 | 56.57 | 61.85 | 65.01 |
|  |  |  | **GIRLS** |  |  |  |  |
| **Age (years)** | **5th centile** | **10th centile** | **25th centile** | **50th centile** | **75th centile** | **90th centile** | **95th centile** |
| 6.00 | 12.77 | 13.15 | 13.91 | 15.08 | 16.87 | 19.64 | 22.34 |
| 7.00 | 14.00 | 14.44 | 15.33 | 16.69 | 18.78 | 21.99 | 25.14 |
| 8.00 | 15.33 | 15.85 | 16.90 | 18.49 | 20.91 | 24.59 | 28.22 |
| 9.00 | 16.77 | 17.38 | 18.62 | 20.48 | 23.26 | 27.42 | 31.48 |
| 10.00 | 18.25 | 18.97 | 20.42 | 22.58 | 25.74 | 30.32 | 34.69 |
| 11.00 | 19.69 | 20.53 | 22.23 | 24.71 | 28.24 | 33.13 | 37.56 |
| 12.00 | 21.01 | 22.00 | 23.96 | 26.78 | 30.66 | 35.73 | 40.02 |
| 13.00 | 22.15 | 23.30 | 25.56 | 28.73 | 32.93 | 38.09 | 42.16 |
| 14.00 | 23.03 | 24.37 | 26.96 | 30.49 | 34.98 | 40.18 | 44.02 |
| 15.00 | 23.57 | 25.12 | 28.06 | 31.96 | 36.69 | 41.87 | 45.50 |
| 16.00 | 23.68 | 25.48 | 28.80 | 33.05 | 37.98 | 43.08 | 46.48 |
| 17.00 | 23.39 | 25.47 | 29.22 | 33.82 | 38.88 | 43.87 | 47.05 |
| 18.00 | 22.74 | 25.18 | 29.43 | 34.39 | 39.56 | 44.40 | 47.38 |
