# Supplementary material for: Cardiovascular magnetic resonance normal values in children for biventricular wall thickness and mass
Source: J Cardiovasc Magn Reson. 2021 Jan 4;23:1. doi: 10.1186/s12968-020-00692-2 (PMC7780624; doi:10.1186/s12968-020-00692-2)

**Figure S1.** Graphs showing myocardial thickness in all 16 segments of the LV by BSA and gender. Of note, graphs for segments 6, 9, 10, 11 and 12 in female are missing as there was no suitable model to fit. However, the corresponding centile tables are provided in the Supplement Table section.


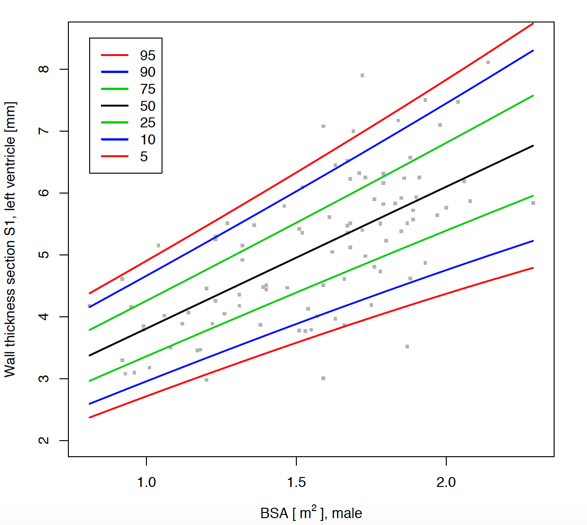

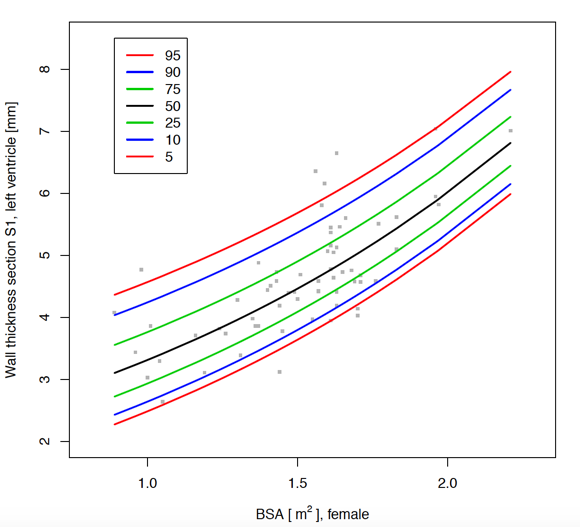

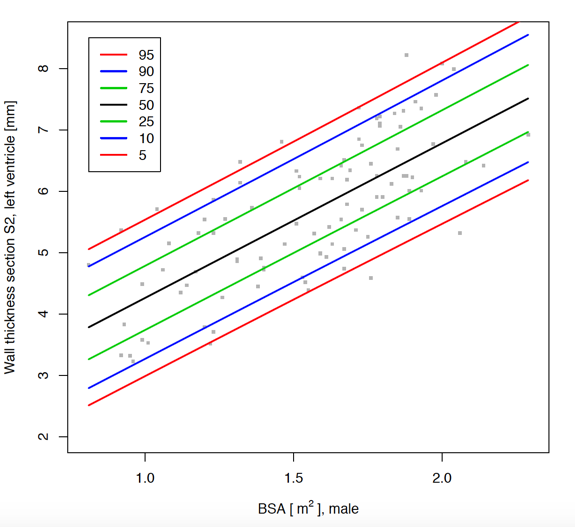

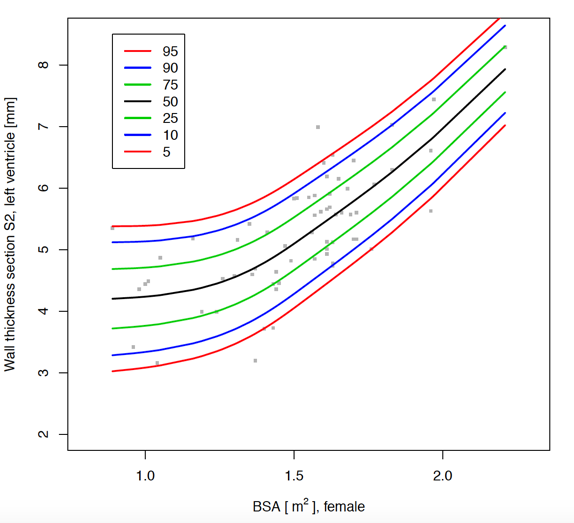


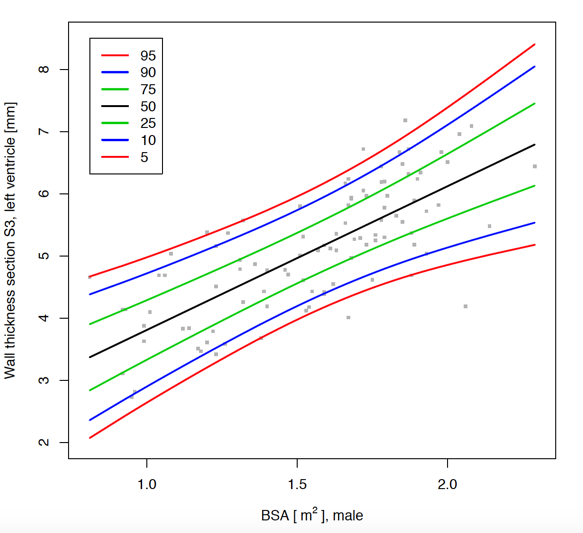

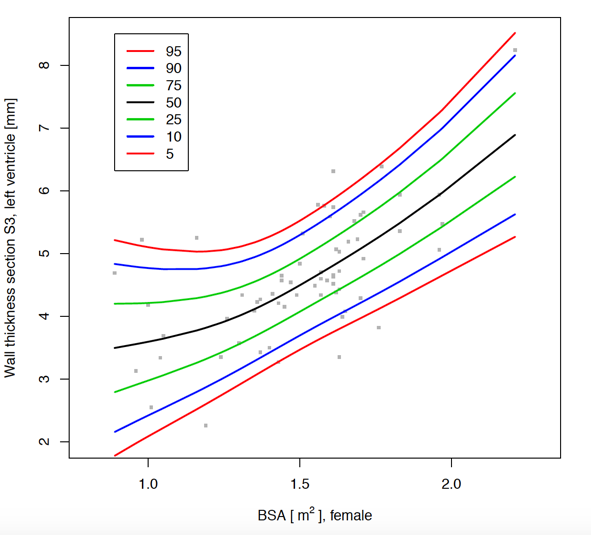


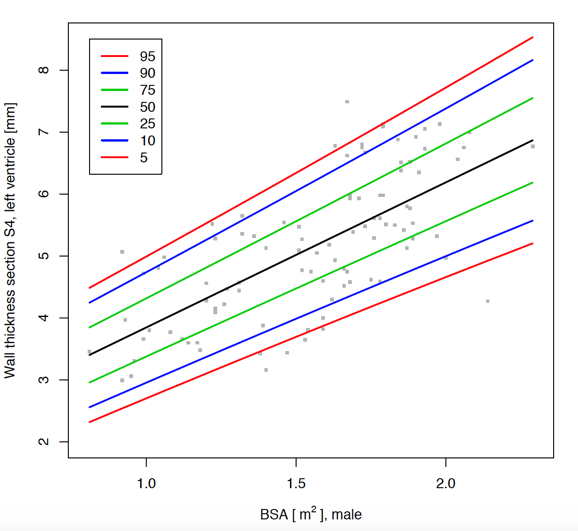

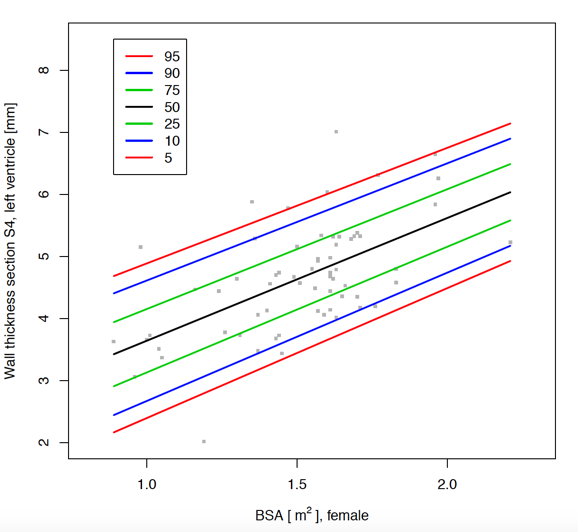


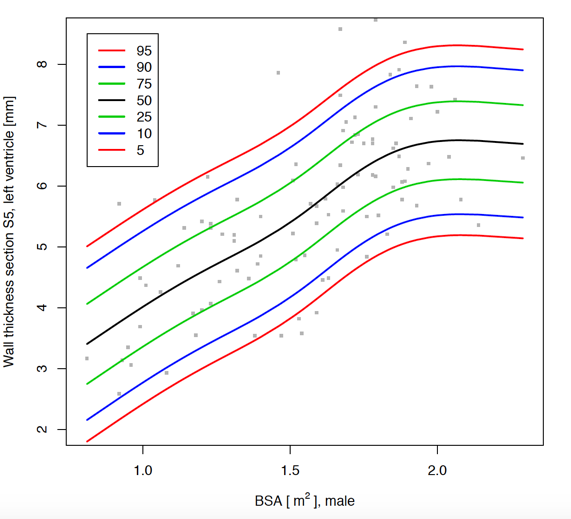

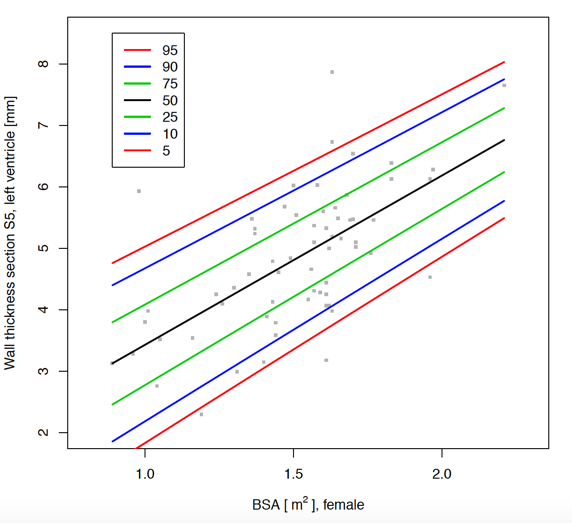


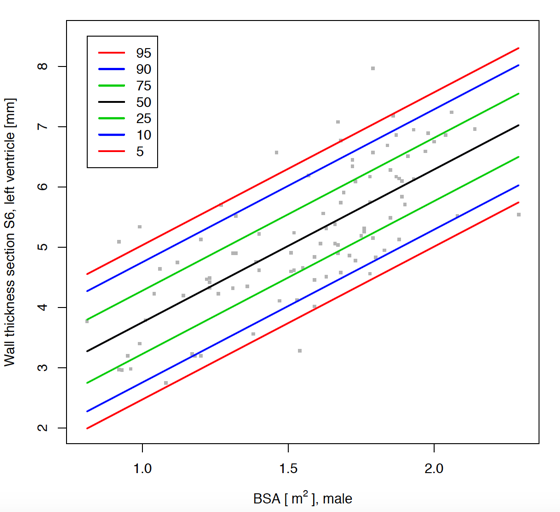


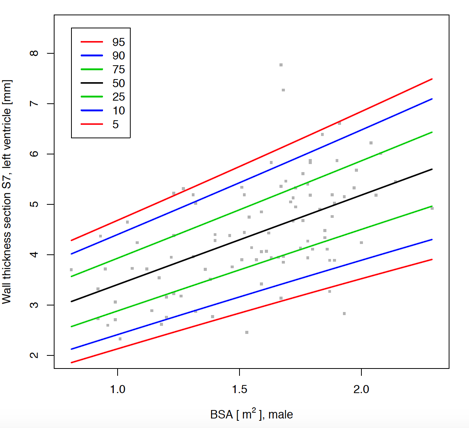

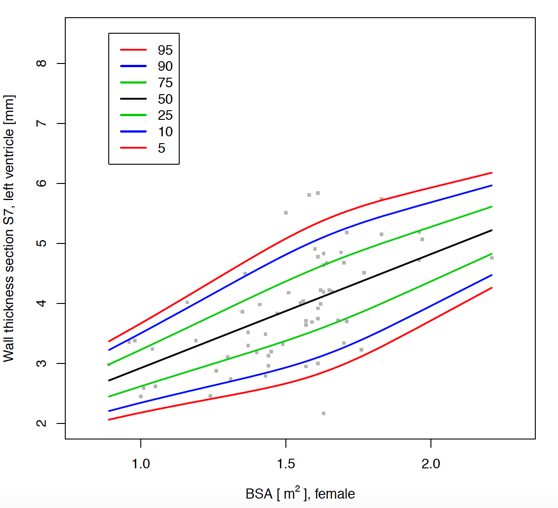


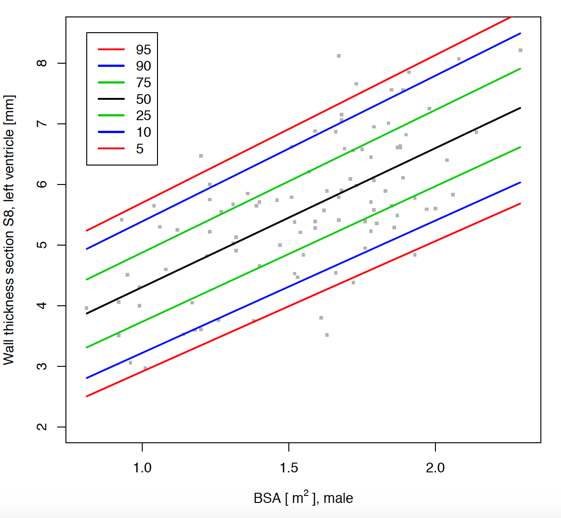

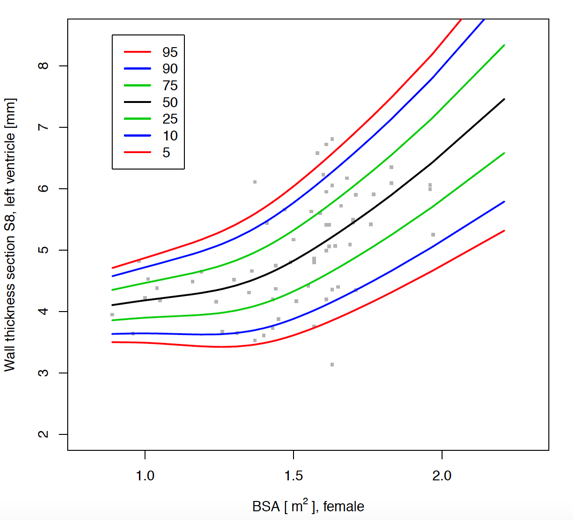


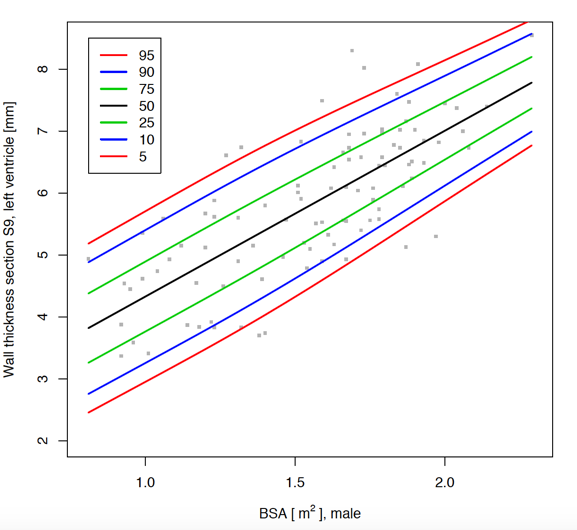

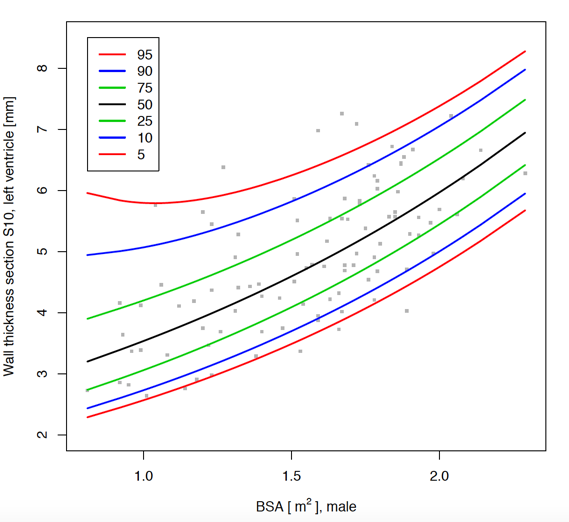


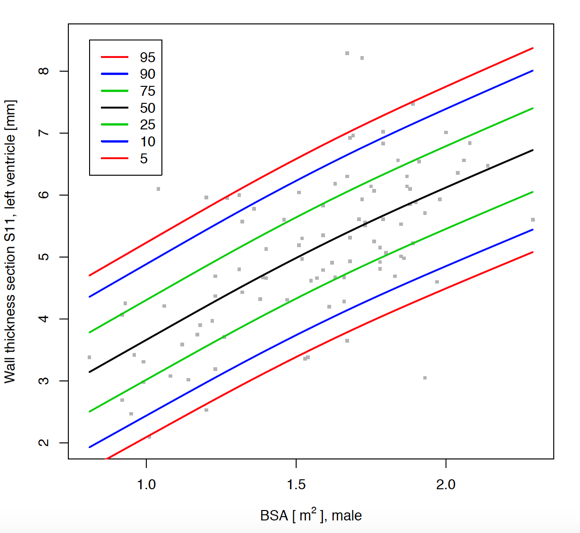

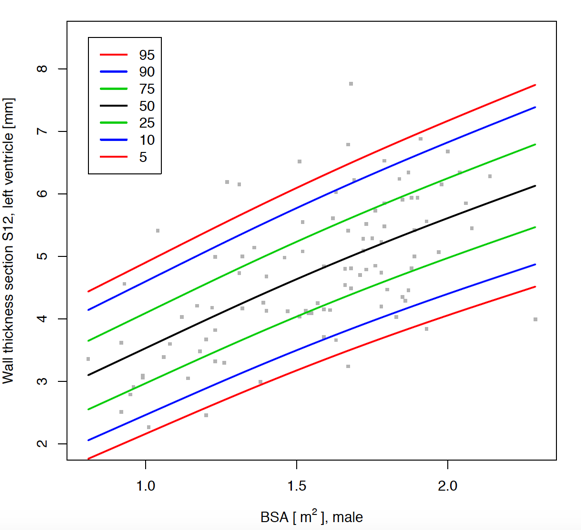


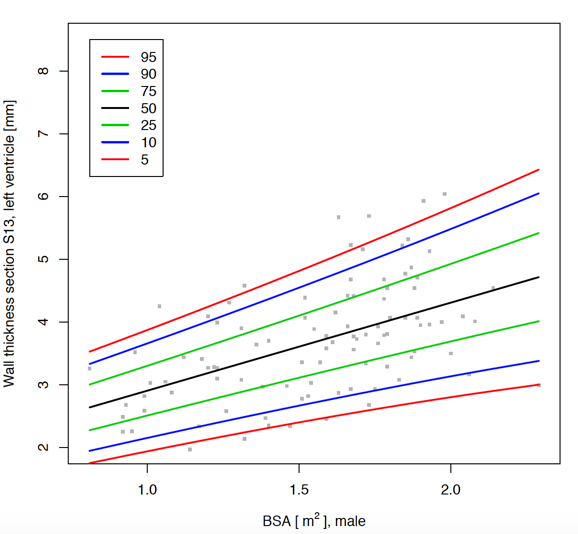

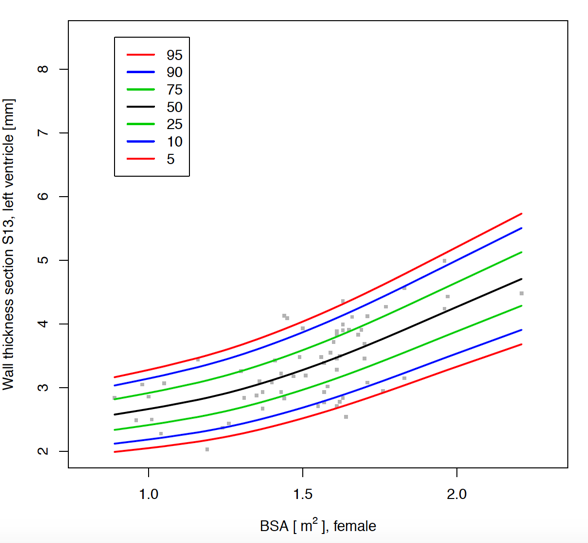


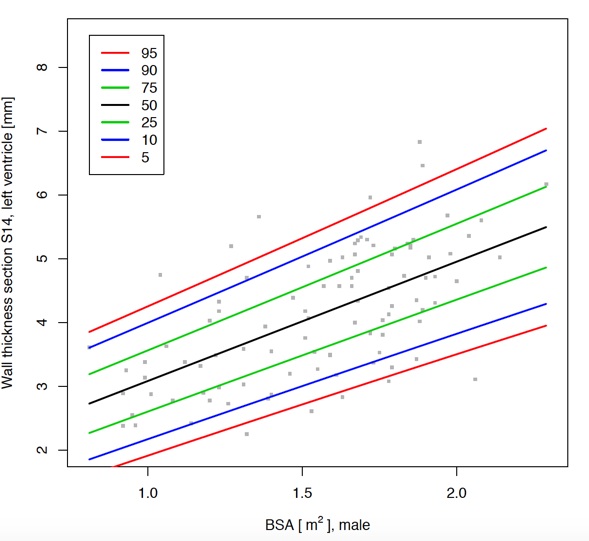

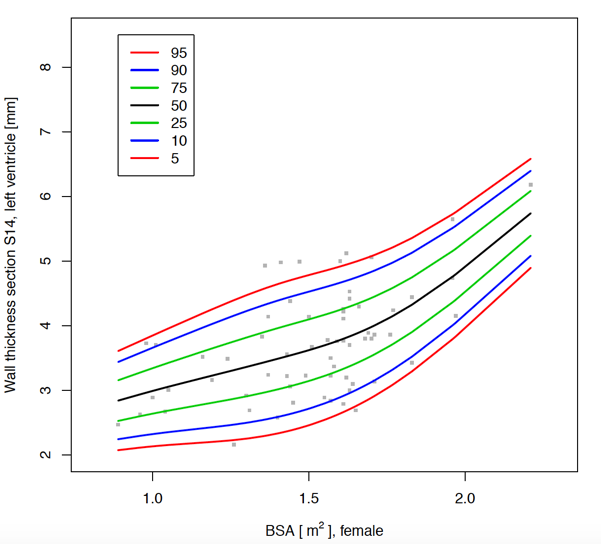


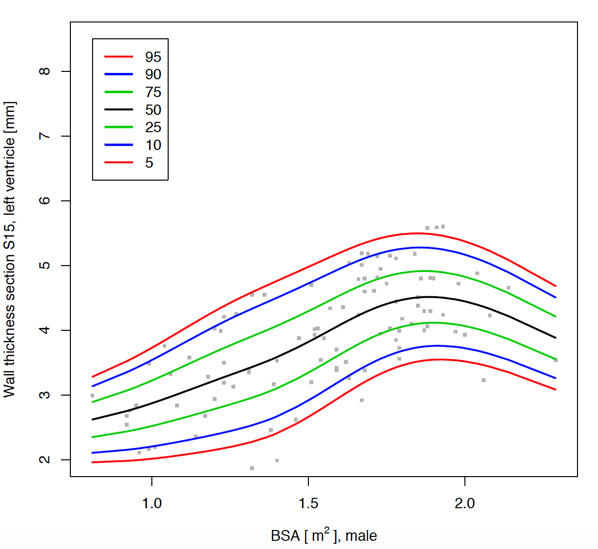

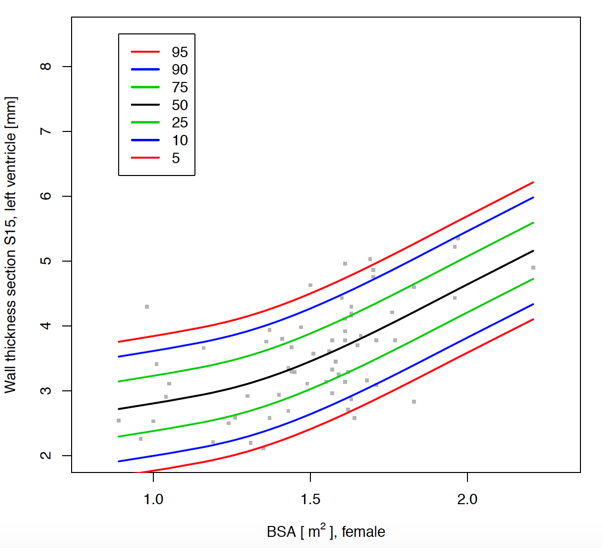


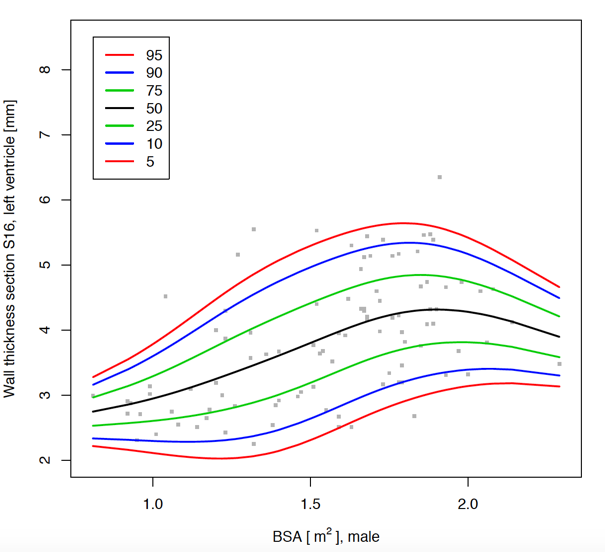

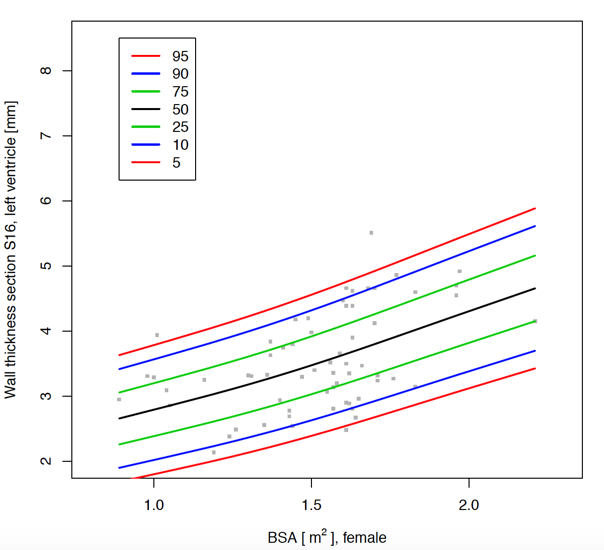


**Figure S2.** Graphs showing myocardial thickness in all 16 segments of the LV by age broken down into boys and girls.

**
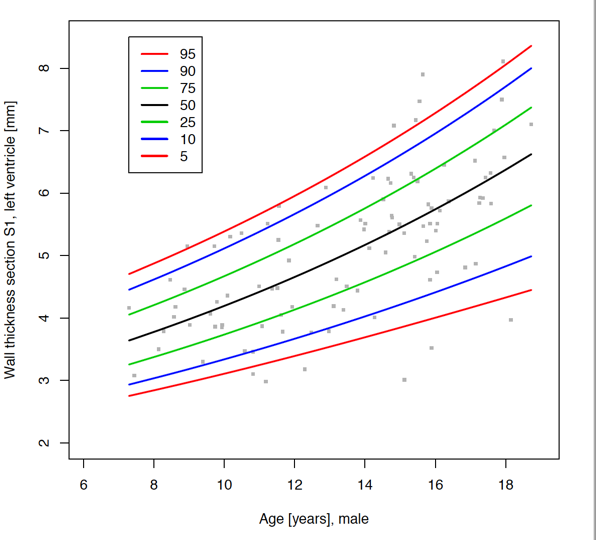

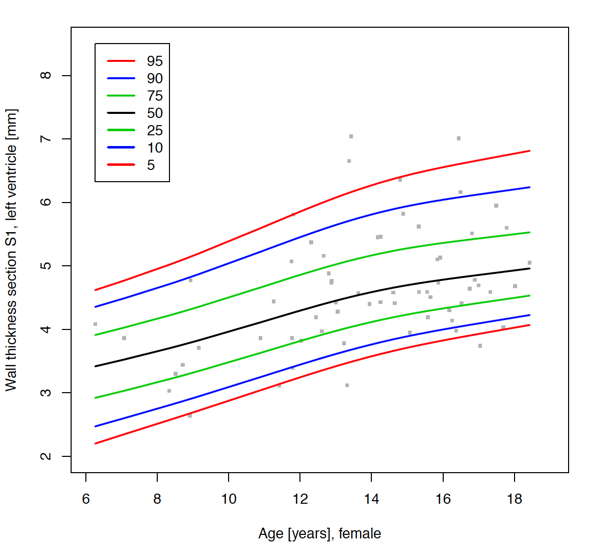

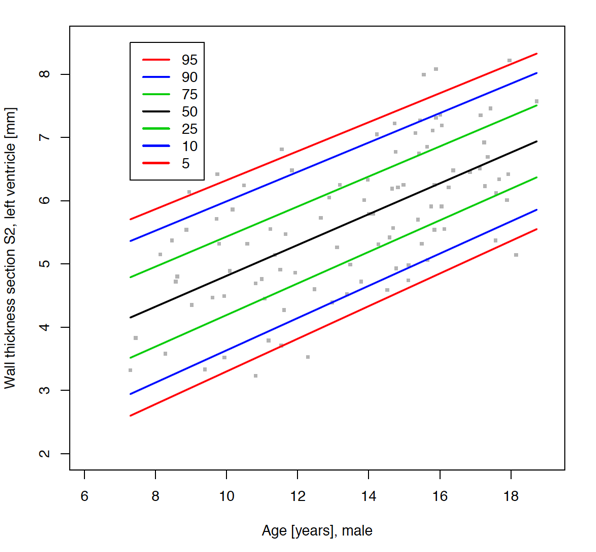

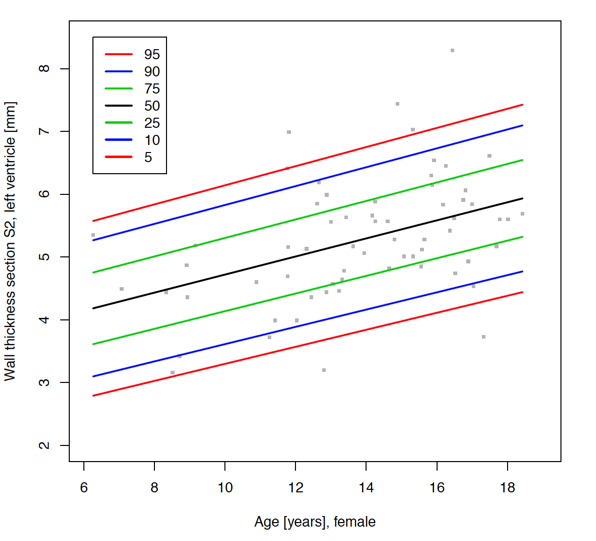

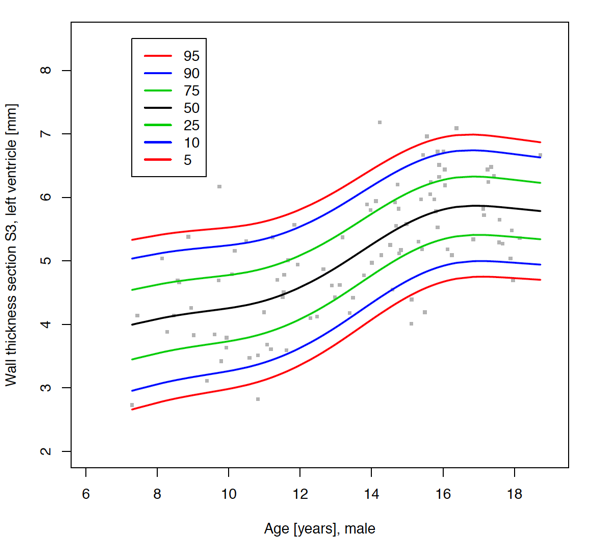

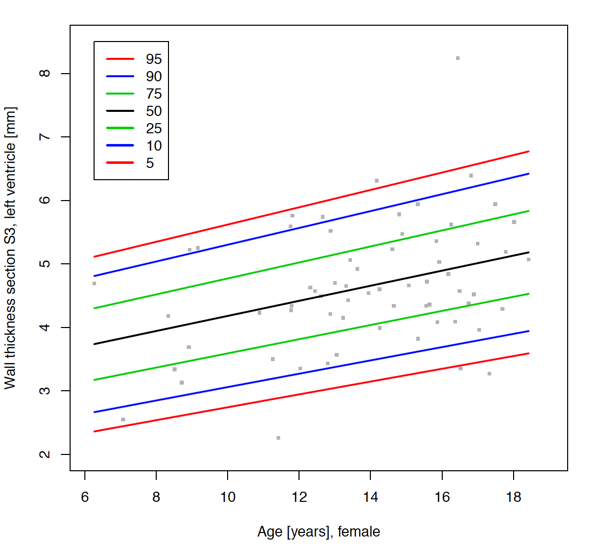

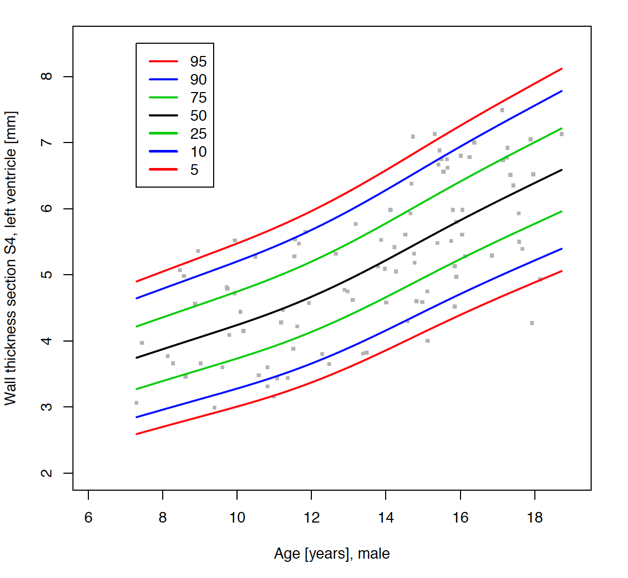

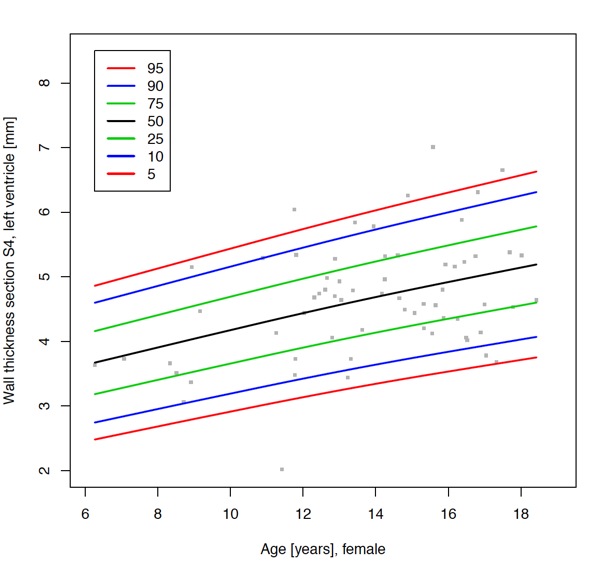

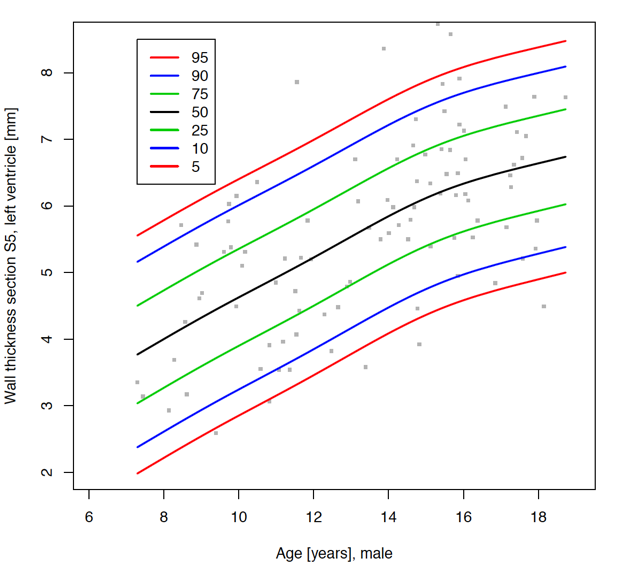

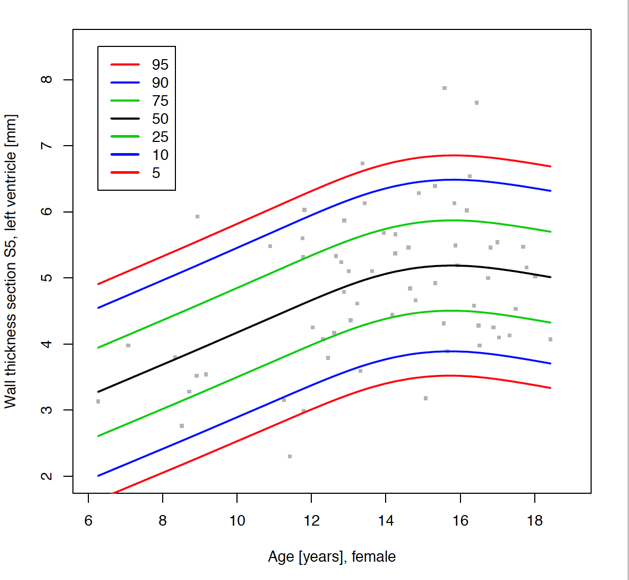

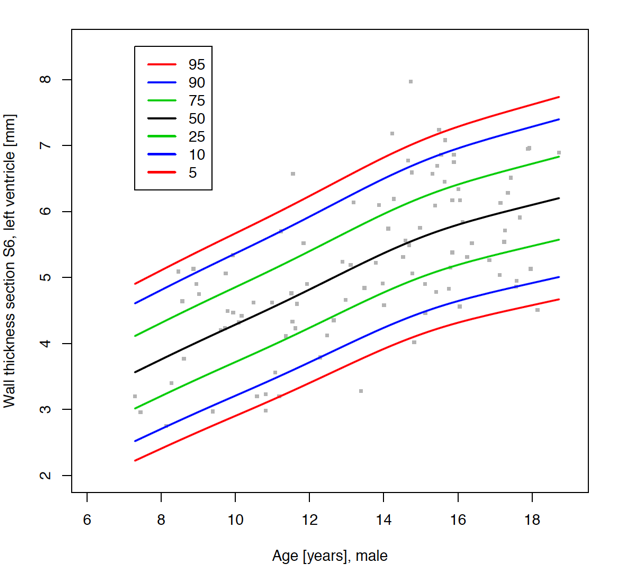

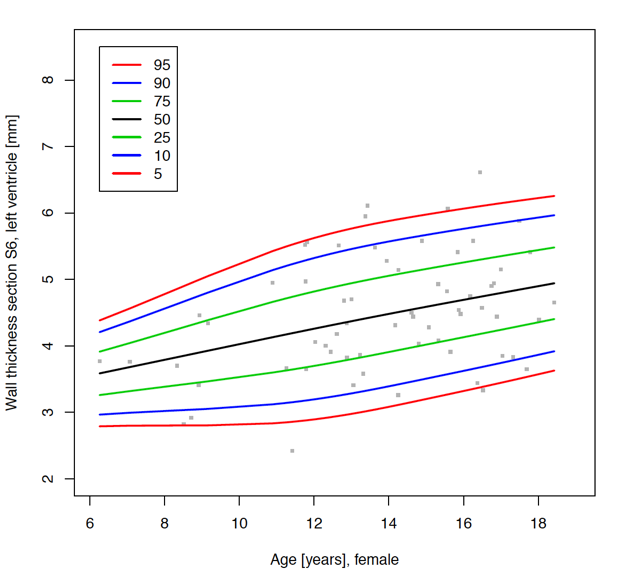

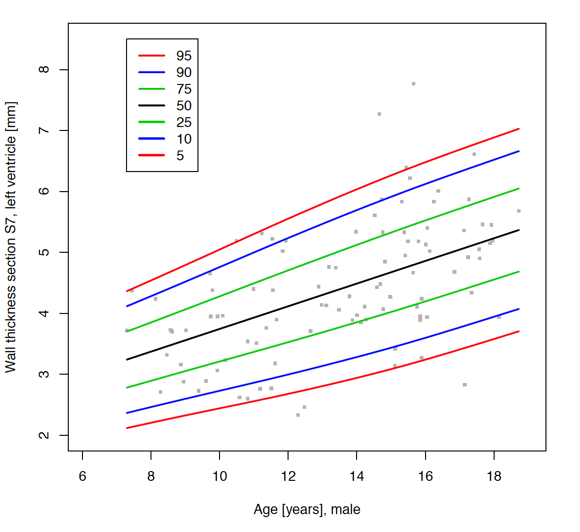

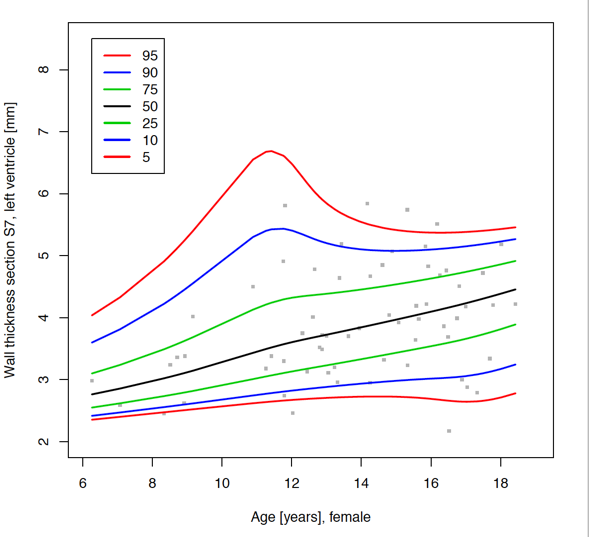

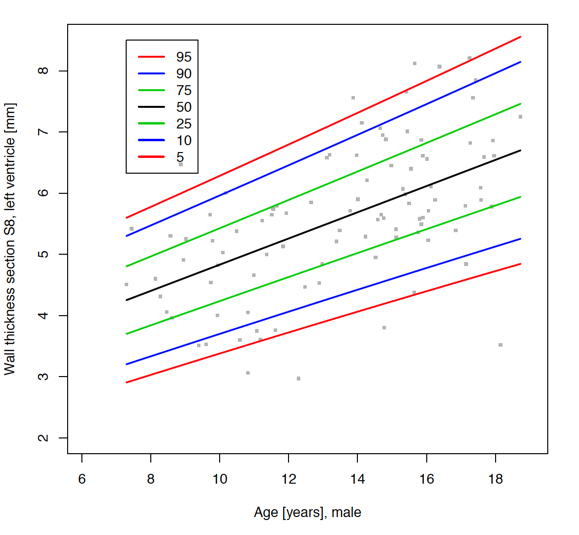

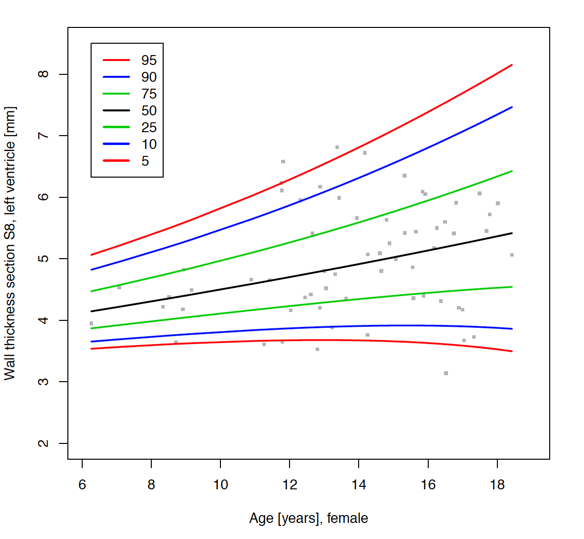

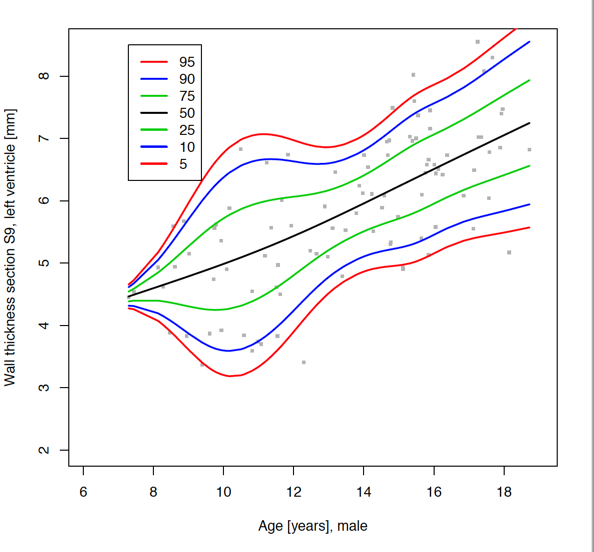

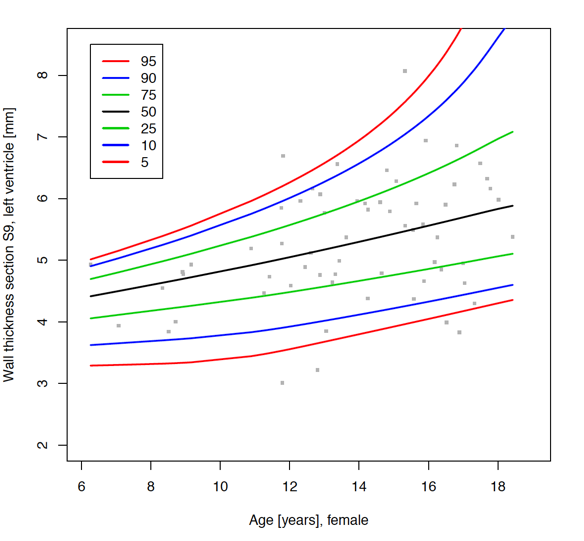

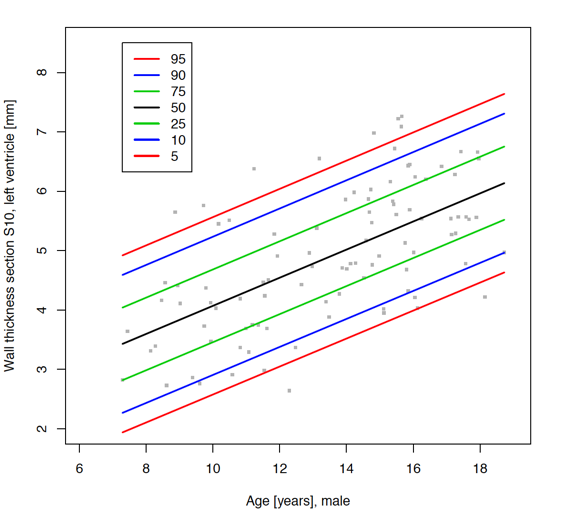

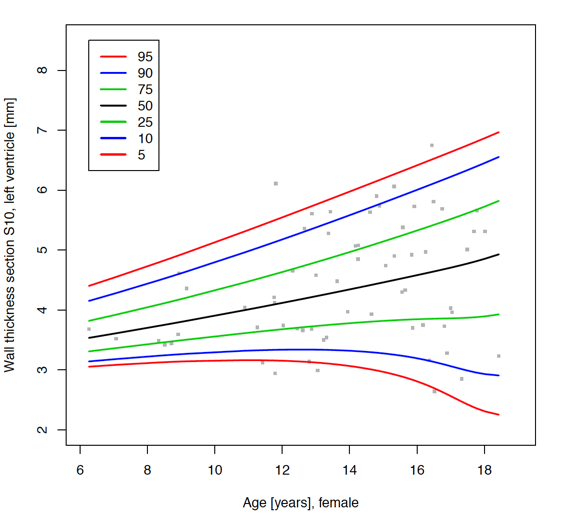

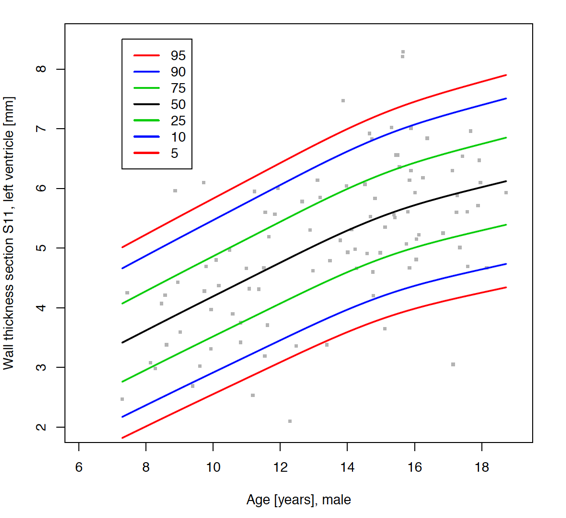

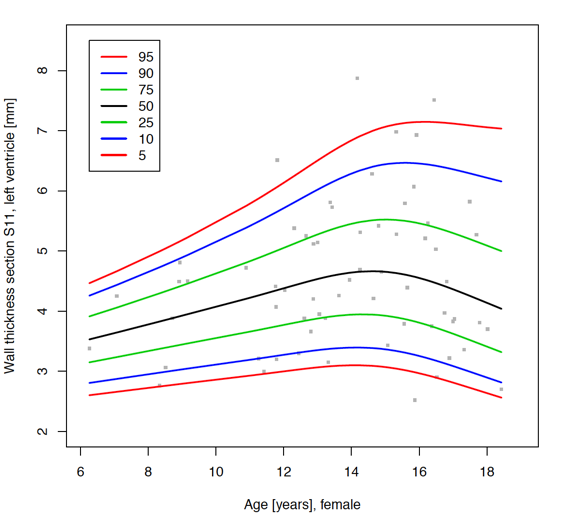

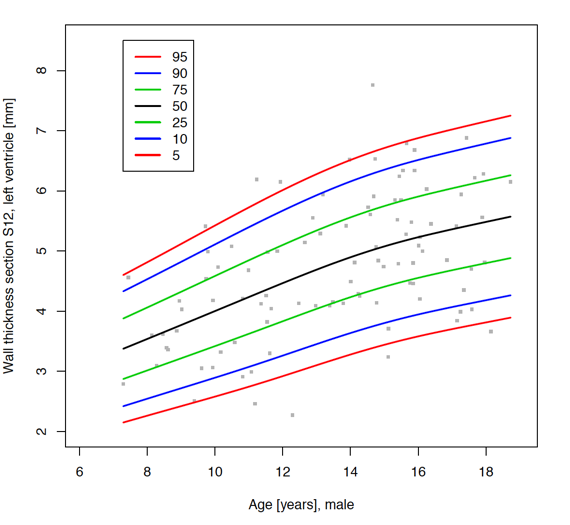

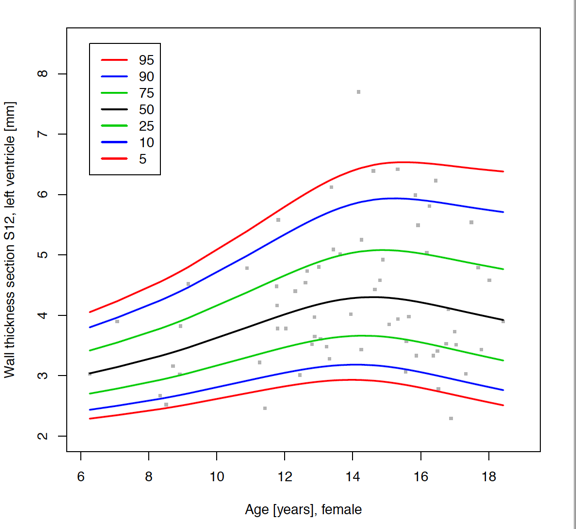

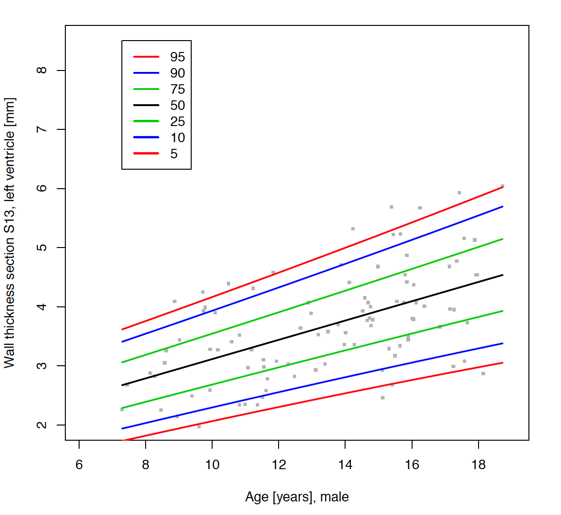

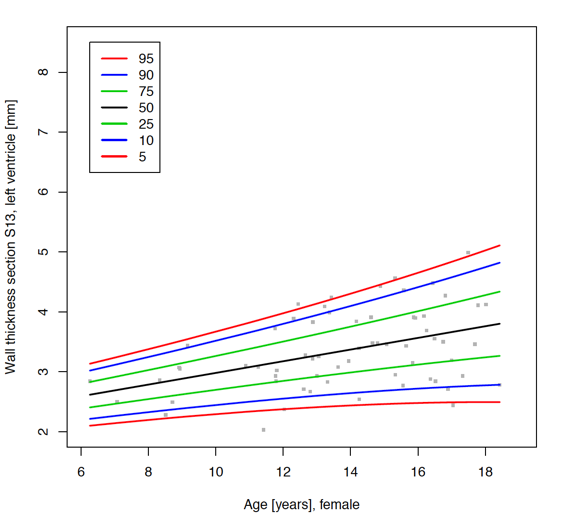

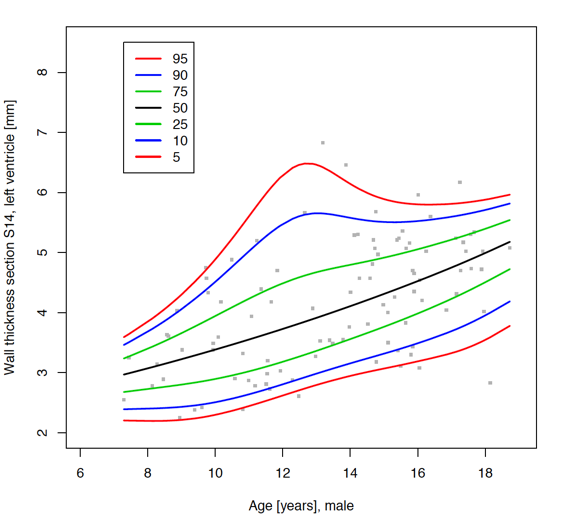

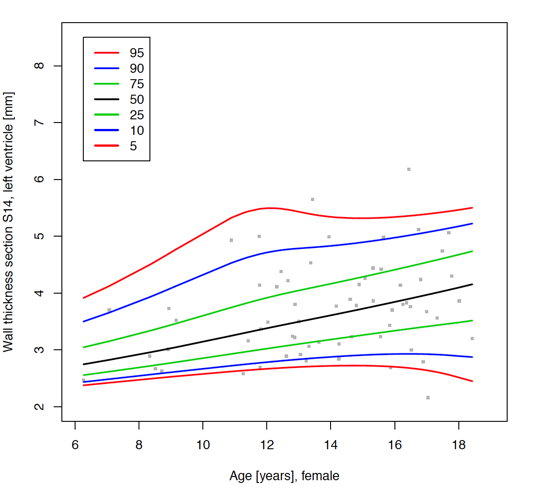

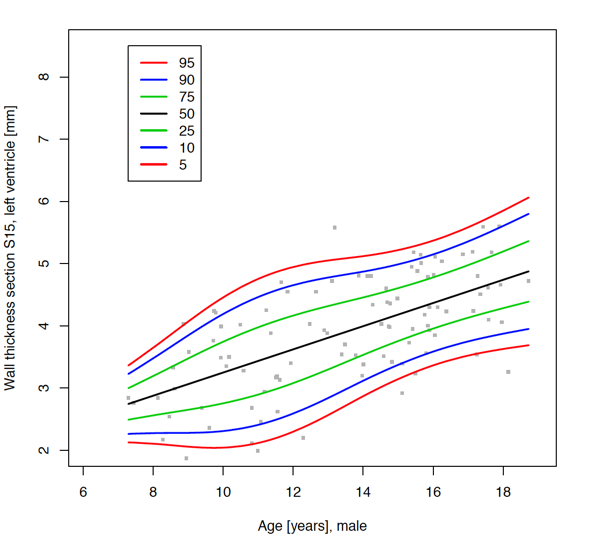

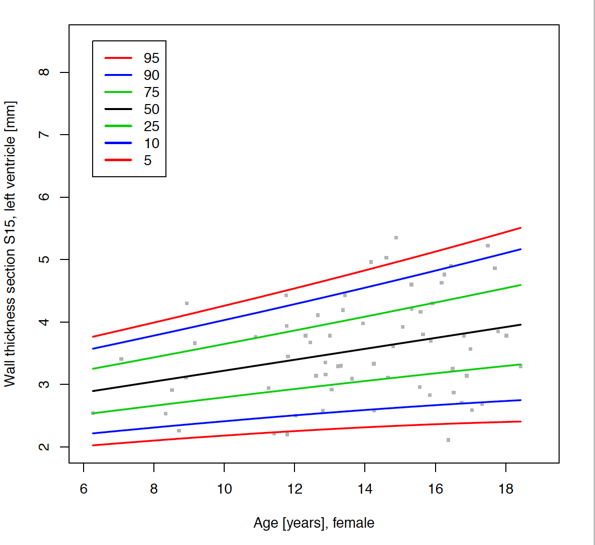

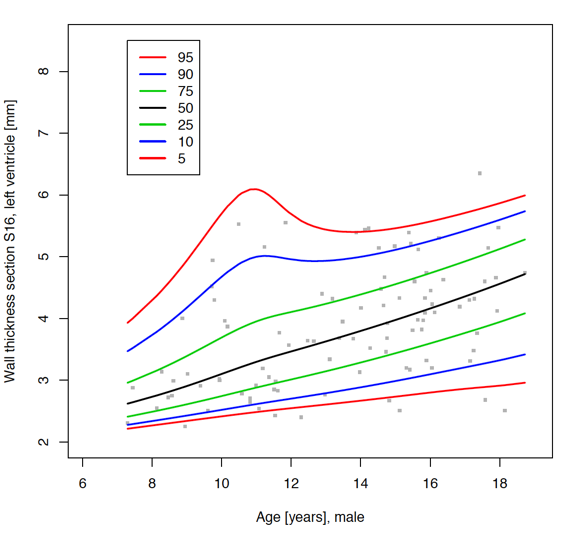

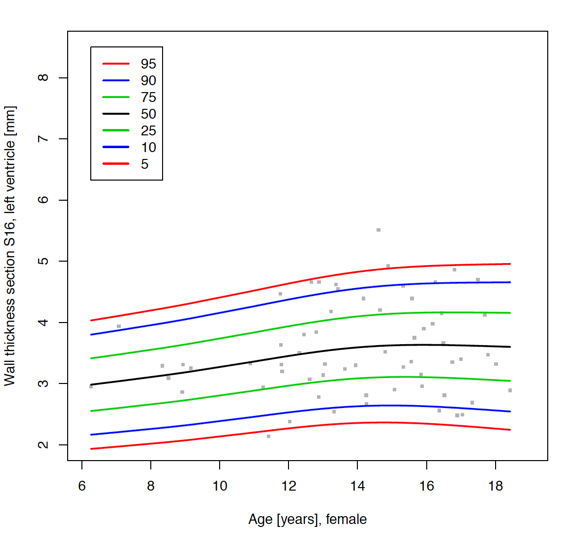
**

**Figure S3.** Graphs showing myocardial thickness in lateral and inferior segments at the basal, midventricular and apical level of the RV by BSA and gender.


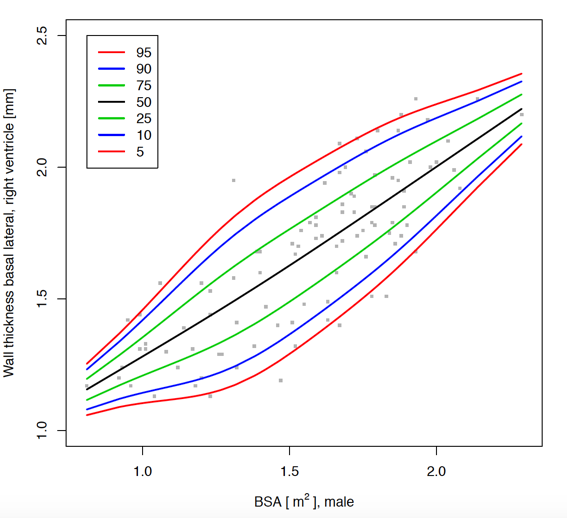

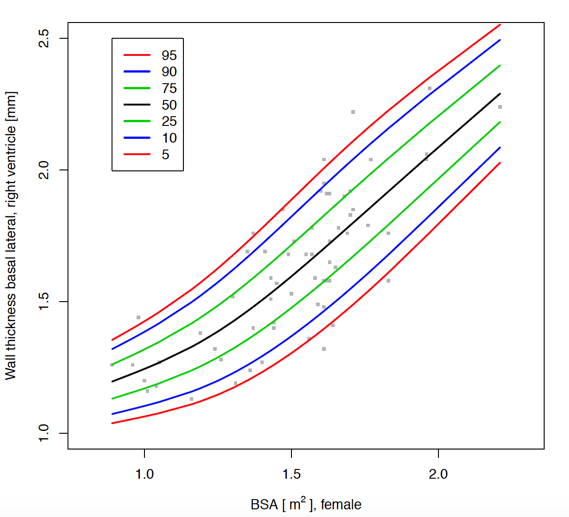


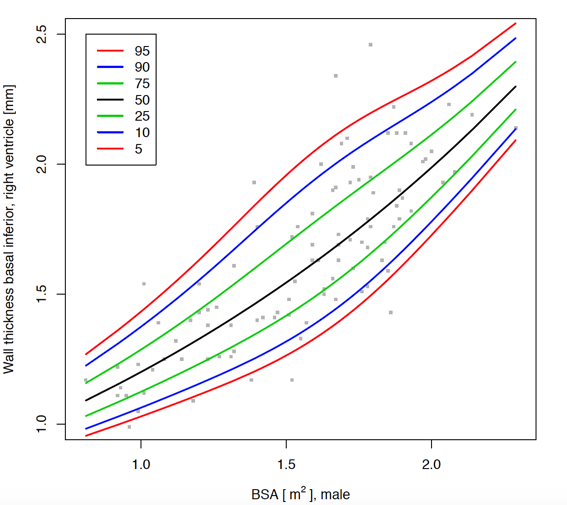

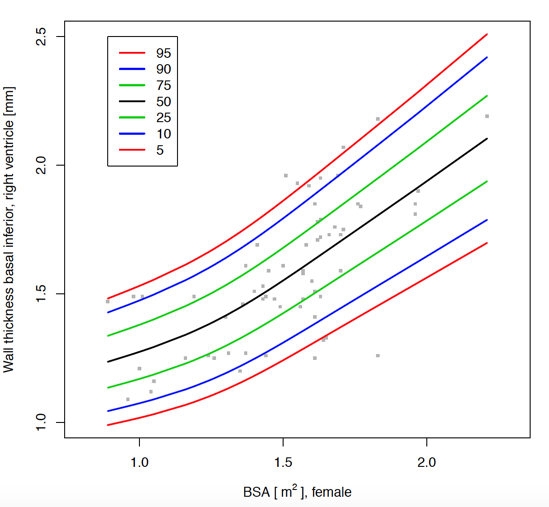


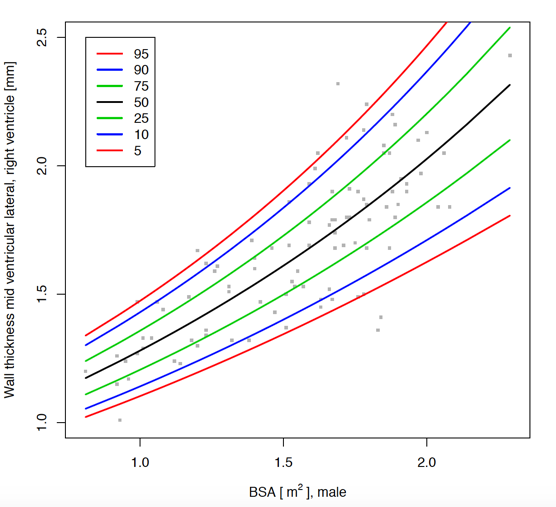

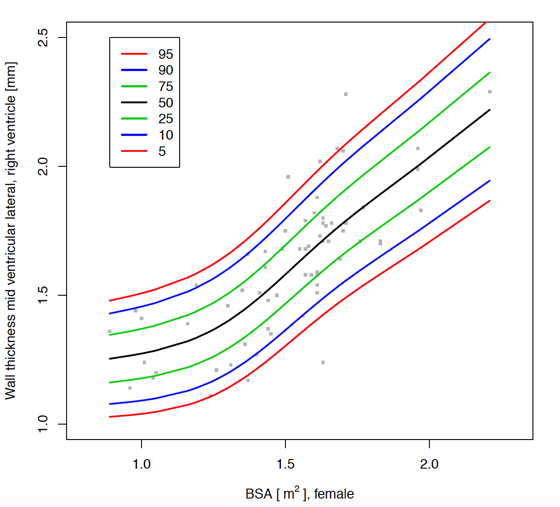

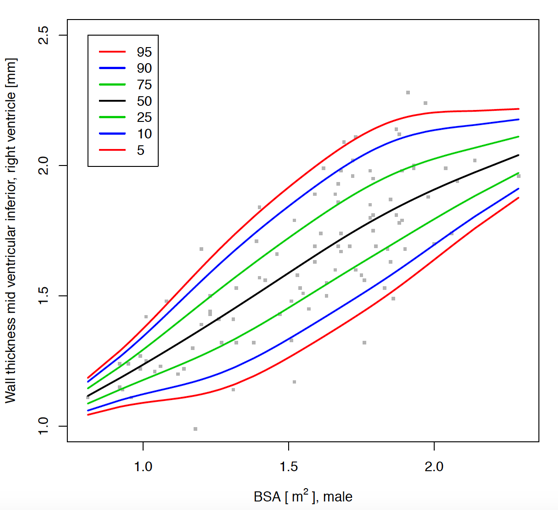

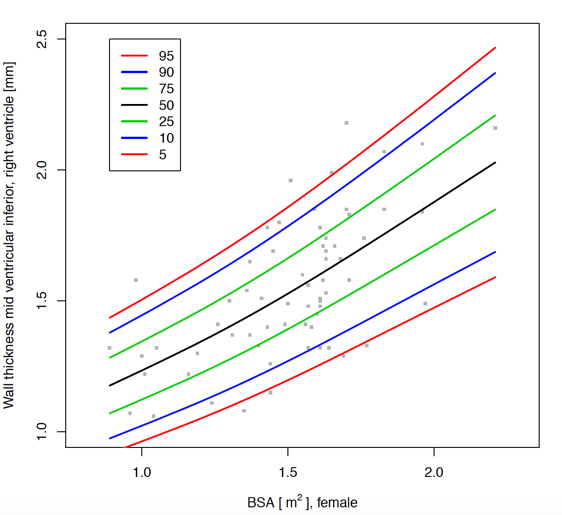


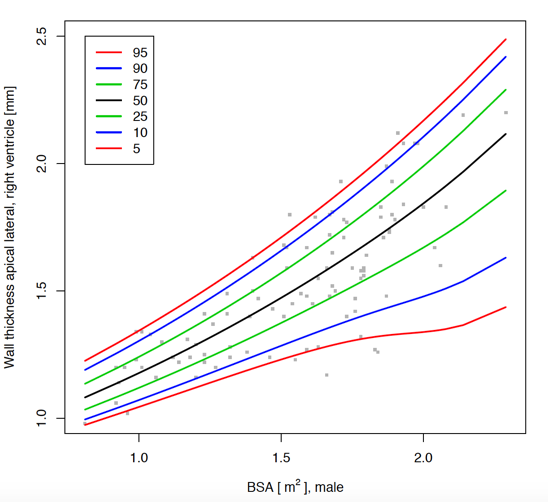

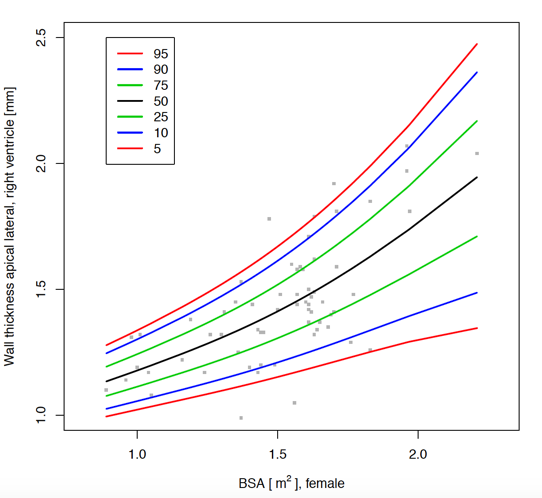

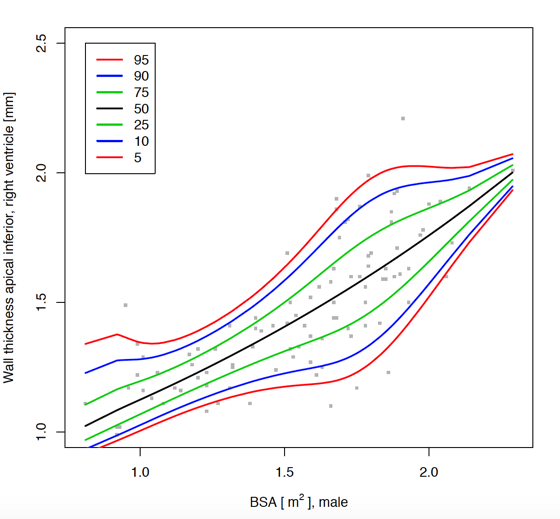

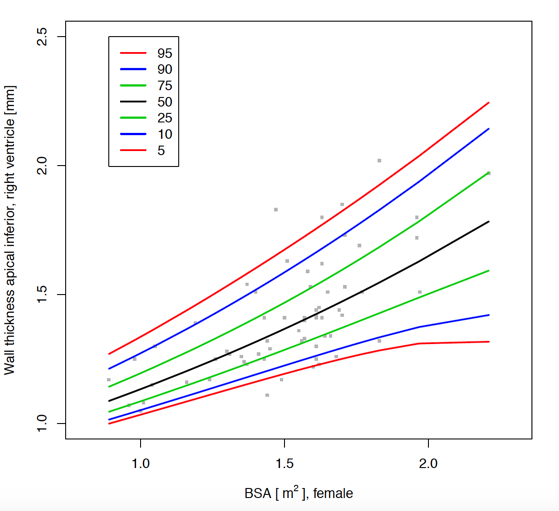


**Figure S4.** Graphs showing myocardial thickness in lateral and inferior segments at the basal, midventricular and apical level of the RV by age broken down into boys and girls.

**
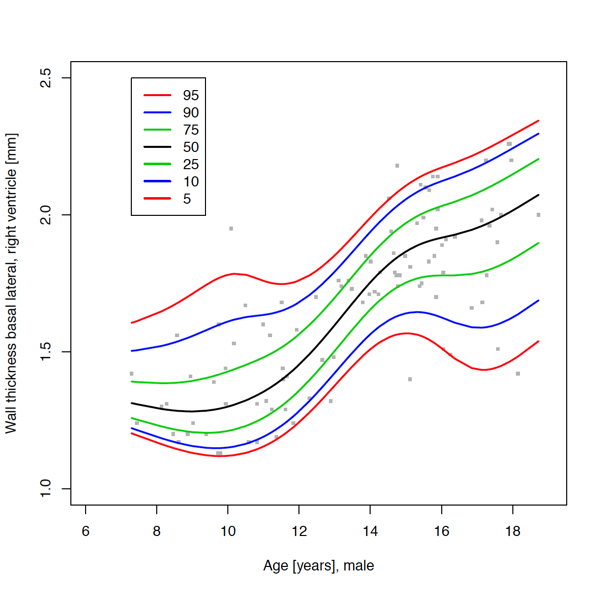

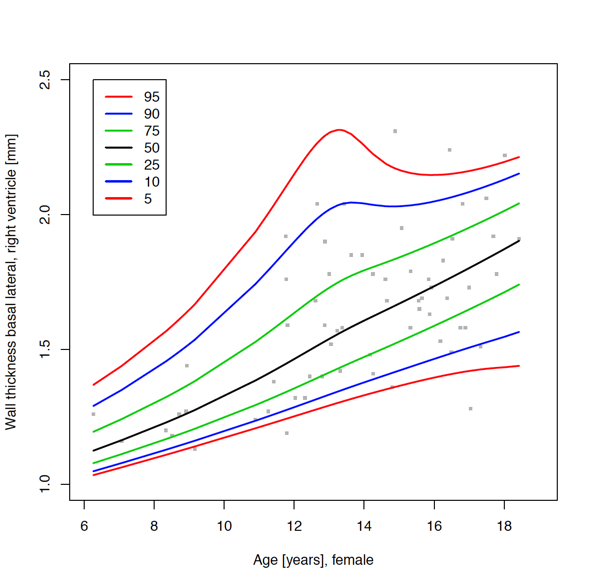

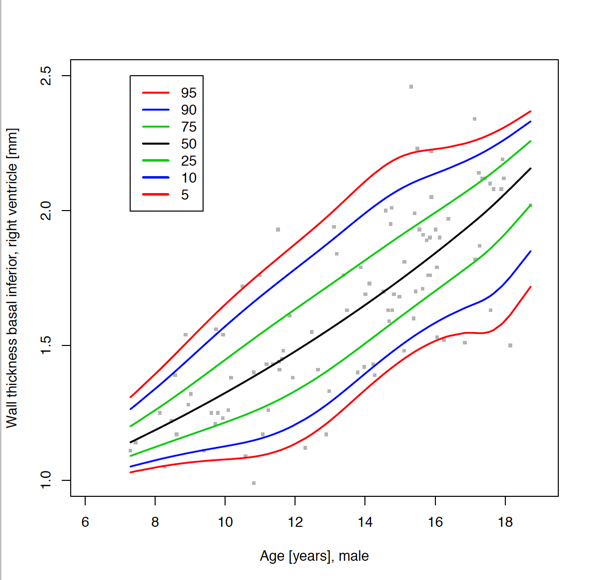

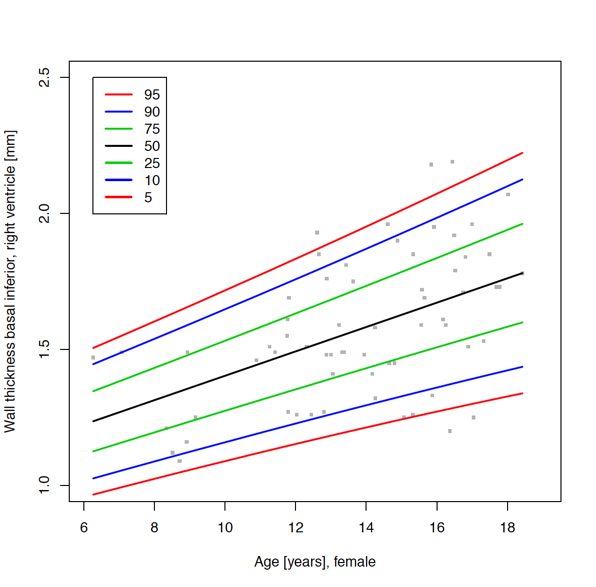
**


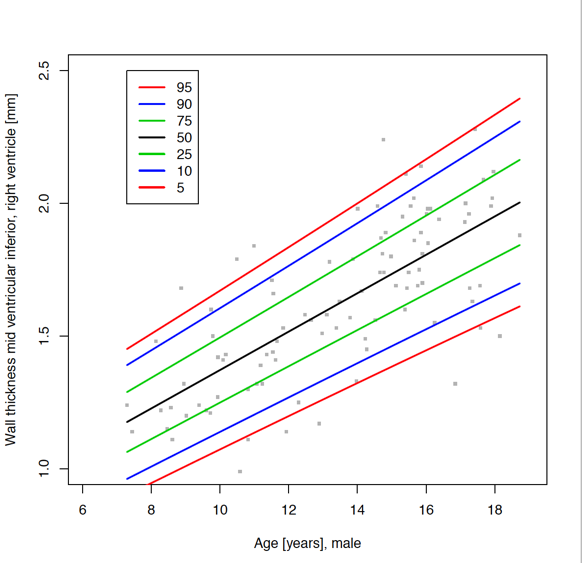

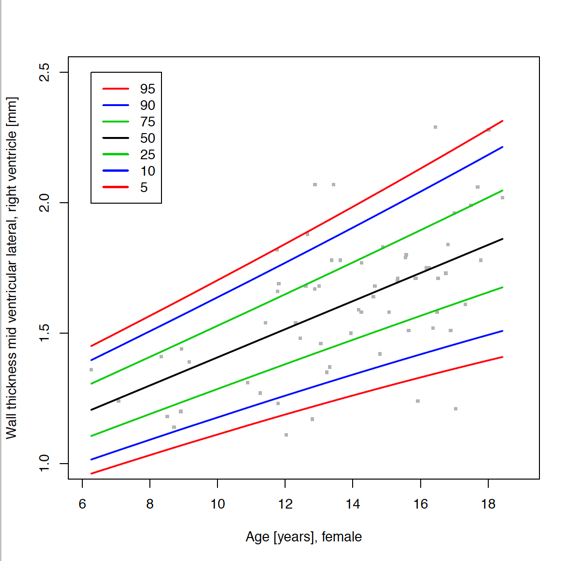

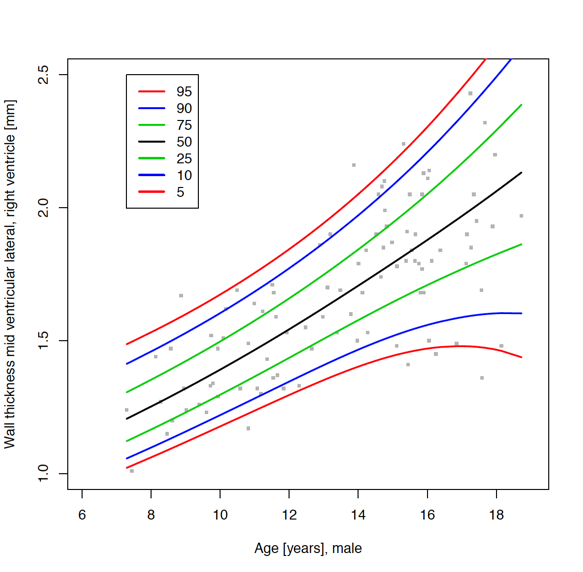

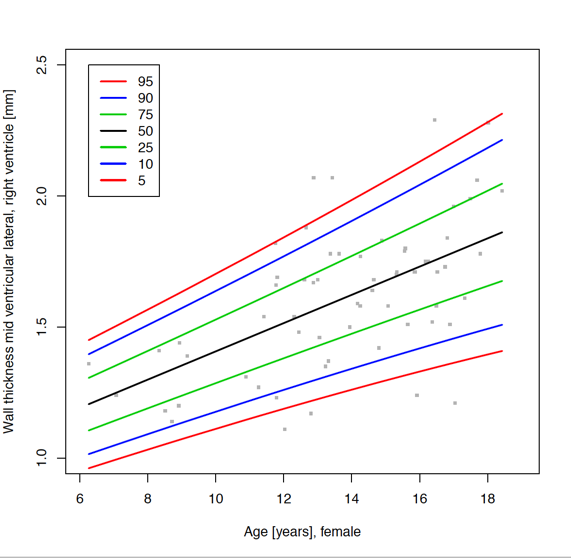


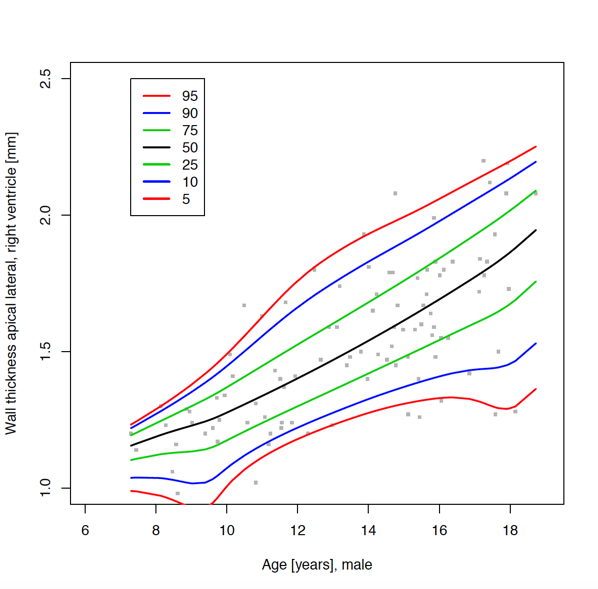

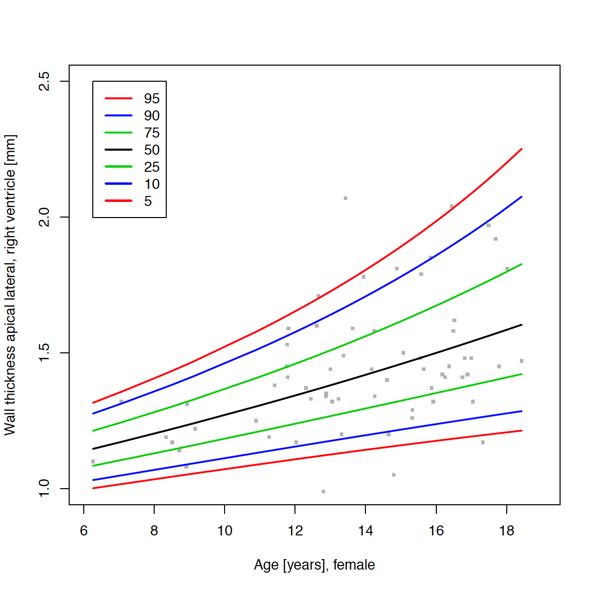

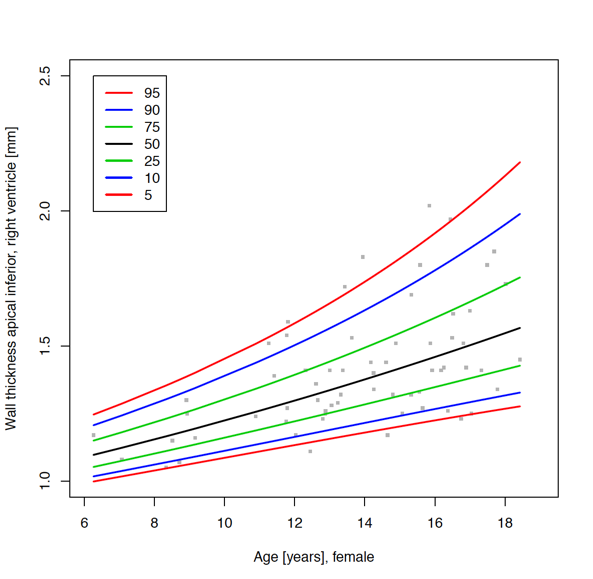

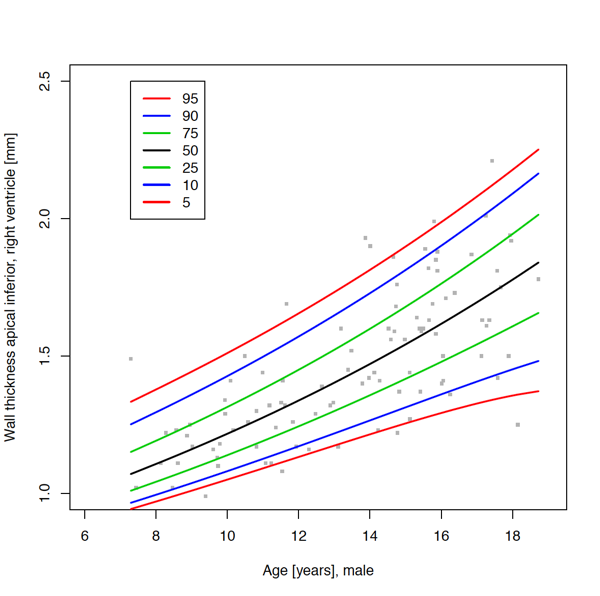

Supplement: Supplementary file 2 — Additional file 2. Additional figures. [file 12968_2020_692_MOESM2_ESM.docx]
